# Photoinduced Heterogeneous C−H Arylation by a Reusable Hybrid Copper Catalyst

**DOI:** 10.1002/chem.202000192

**Published:** 2020-02-25

**Authors:** Isaac Choi, Valentin Müller, Gaurav Lole, Robert Köhler, Volker Karius, Wolfgang Viöl, Christian Jooss, Lutz Ackermann

**Affiliations:** ^1^ Institut für Organische und Biomolekulare Chemie Georg-August-Universität Tammanstrasse 2 37077 Göttingen Germany; ^2^ Institut für Materialphysik Georg-August-Universität Friedrich-Hund-Platz 1 37077 Göttingen Germany; ^3^ University of Applied Sciences and Arts Laboratory of Laser and Plasma Technologies Von-Ossietzky-Strasse 99 37085 Göttingen Germany; ^4^ Geowissenschaftliches Zentrum Georg-August-Universität Goldschmidtstrasse 3 37077 Göttingen Germany; ^5^ Woehler Research Institute for Sustainable Chemistry (WISCh) Georg-August-Universität Göttingen Tammannstrasse 2 37077 Göttingen Germany

**Keywords:** C−H arylation, copper catalysis, heterogeneous catalysis, hybrid catalysis, photocatalysis

## Abstract

Heterogeneous copper catalysis enabled photoinduced C−H arylations under exceedingly mild conditions at room temperature. The versatile hybrid copper catalyst provided step‐economical access to arylated heteroarenes, terpenes and alkaloid natural products with various aryl halides. The hybrid copper catalyst could be reused without significant loss of catalytic efficacy. Detailed studies in terms of TEM, HRTEM and XPS analysis of the hybrid copper catalyst, among others, supported its outstanding stability and reusability.

## Graphical abstract

**Let there be light**: C−H arylations were realized at room temperature with a hybrid copper catalyst, enabling catalyst recycling and reuse.

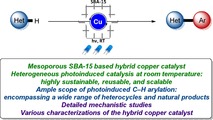

The activation of otherwise inert C−H bonds has emerged as a transformative tool for the step‐economical diversification in molecular sciences.^1^ Although C−H activation has to date predominantly exploited precious, toxic 4d transition metals, significant progress has been recently realized with the aid of earth‐abundant and cost effective 3d metal catalysts.^2^ Particularly, copper‐catalyzed^3^ C−H arylation has recently witnessed a considerable impetus, with notable contributions by Daugulis,^4^ Miura^5^ and Ackermann,^6^ among others.^3^ Despite these major advances, copper‐catalyzed C−H activation with aryl halides has been severely restricted by their harsh reaction conditions with reaction temperatures ranging from 120 to 160 °C.

In recent years, photocatalysis^7^ has been identified as an increasingly powerful approach towards various sustainable organic syntheses,^8^ such as C−N bond formations extensively elaborated by Fu,^9^ MacMillan,^10^ and Kobayashi.^11^ Thus, photoredox C−H functionalizations proved viable, although predominantly relying on precious transition metals, such as rhodium, palladium, and ruthenium complexes.^12^ In contrast, we have very recently devised photoinduced C−H arylations and chalcogenations by less toxic base metal catalysts (Figure 1 a).^13^ In spite of notable progress, photoinduced organometallic C−H activations were thus far limited to homogeneous catalysis, often leading to undesired trace metal impurities in the target products, and, more importantly, inherently preventing catalysts from reuse.^14^ Although selected silica‐supported catalysts^15^ with non‐excited‐state reactivity were elegantly developed by Jones/Davies^16^ and Sawamura,^17^ heterogeneous catalysis for photoinduced C−H activation has thus far unfortunately proven elusive.

**Figure 1:**
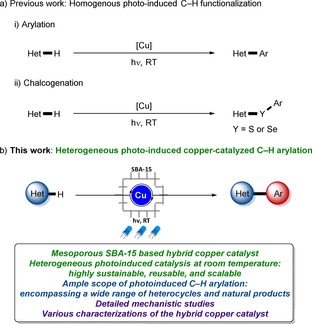
Photoinduced hybrid copper catalyzed C−H arylation.

Within our program on sustainable C−H activation,^18^ we have now unraveled the first photoinduced heterogeneous copper‐catalyzed C−H arylation, on which we report herein (Figure 1 b). Salient features of our strategy include a) photoinduced C−H arylation by hybrid copper catalyst under exceedingly mild conditions, b) broadly applicable and reusable hybrid copper catalyst for photoinduced C−H arylation, and c) detailed mechanistic studies for photoinduced heterogeneous catalysis and spectroscopic analysis of the reusable hybrid copper catalyst.

We initiated our studies by probing representative reaction conditions for the envisioned C−H arylation of heteroarenes by using tailor‐made, silica‐supported hybrid copper catalyst (Table 1).^19^ Thus, the desired C−H arylated product **3 aa** was obtained with the reusable hybrid copper catalyst under mild photoinduced conditions (entry 1). The sole use of copper iodide fell short in efficiently delivering the desired product (entry 2). Control experiments verified the essential role of the Hybrid‐Cu catalyst (entries 3–5).

**Table 1:** Establishing photoinduced C−H arylation by the hybrid copper catalyst.

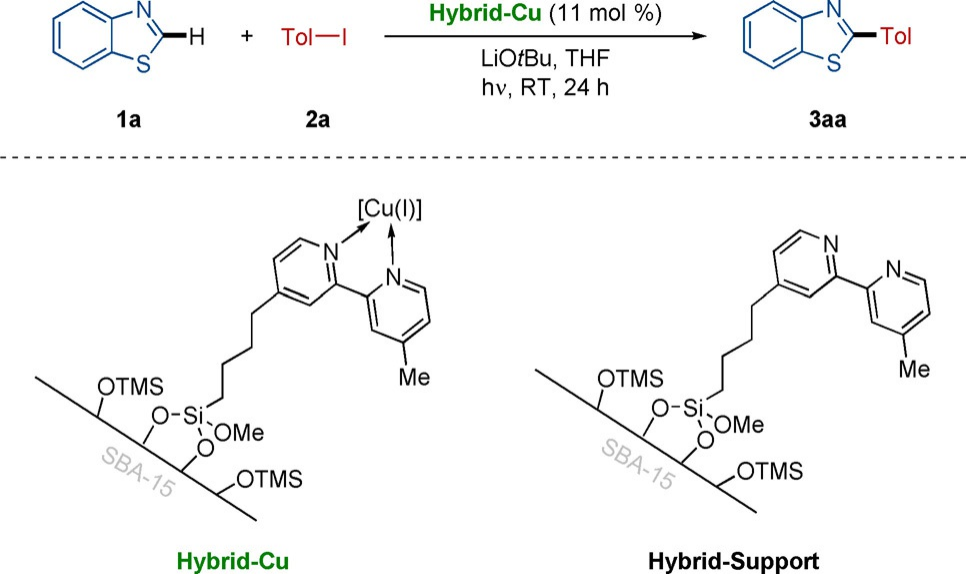		
Entry	Deviation from standard conditions	Yield [%]^[a]^
1	standard conditions	93 (85)^[b,c]^
2	CuI instead of Hybrid‐Cu	58
3	Hybrid‐Support instead of Hybrid‐Cu	traces
4	without Hybrid‐Cu	traces
5	reaction in the dark	traces

[a] Reaction conditions: **1 a** (0.25 mmol), **2 a** (1.25 mmol), Hybrid‐Cu (11 mol %), LiO*t*Bu (0.75 mmol), Et_2_O (0.5 mL), 254 nm, RT, 24 h, isolated yield. [b] Average yield of two runs. [c] The yield in parentheses is the result with the reused Hybrid‐Cu.

With the optimal reaction conditions in hand, we explored the versatility of the hybrid copper catalysis for the photoinduced C−H arylation of thiazoles **1** or oxazoles **4** with diversely substituted aryl iodides **2** (Scheme 1). Thus, the robust hybrid copper catalyst smoothly enabled the photoinduced C−H arylation with high functional group tolerance, featuring electron‐rich and electron‐deficient aryl halides **2**, including sensitive aryl chlorides and bromides. The photoinduced C−H arylation of azoles occurred with high levels of site selectivity, exclusively delivering C2 arylated products.

**Scheme 1:**
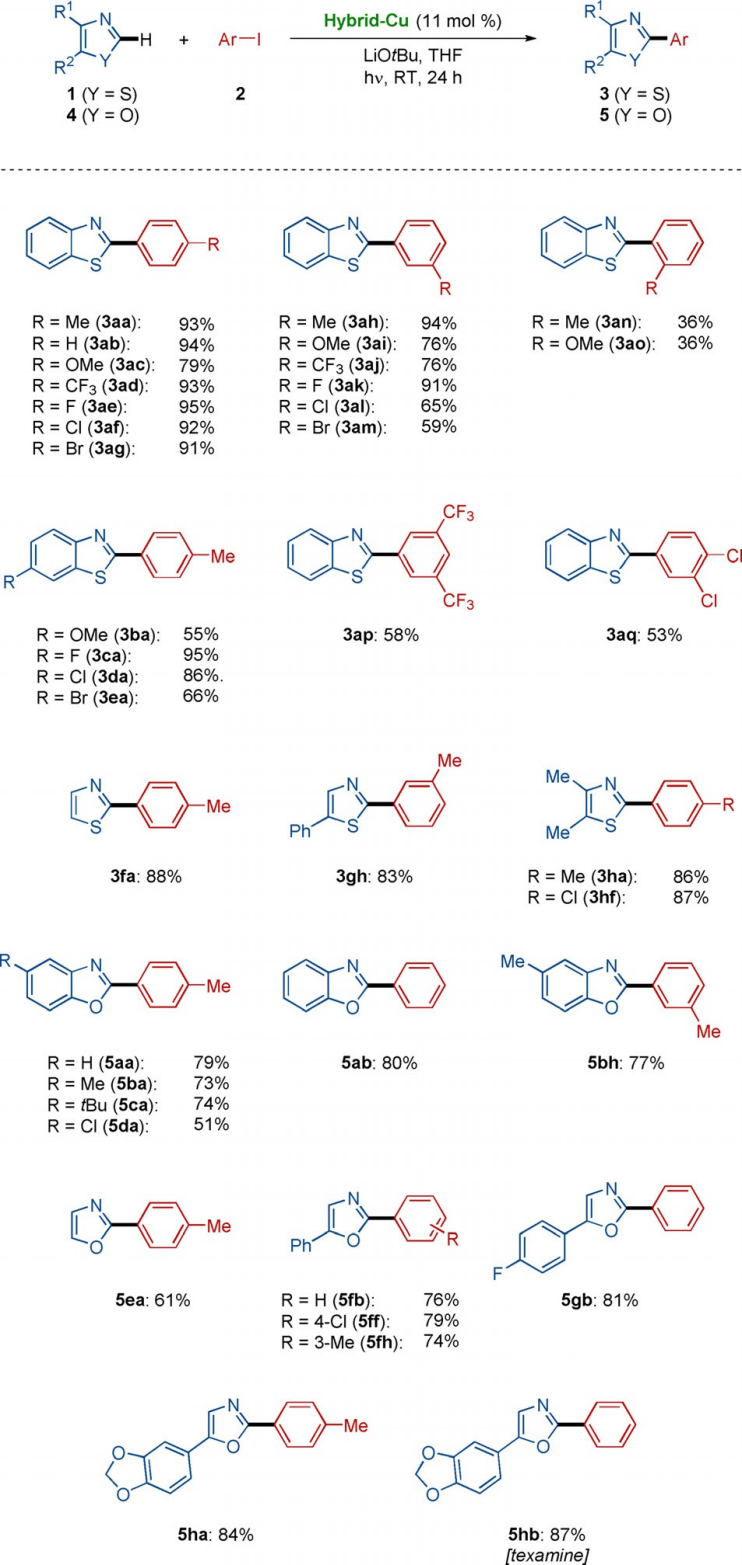
Photoinduced heterogeneous C−H arylation.

The versatile photoinduced heterogeneous C−H arylation was not limited to the heterocycles **1** and **4** with relatively acidic C−H bonds,^20^ but *N*‐methyl benzimidazoles **6** were also found to be viable substrate (Scheme 2).^19^ Indeed, the heterogeneous photocatalysis for C−H arylation of *N*‐methyl benzimidazoles **6** were likewise accomplished with a set of diversely decorated aryl iodides **2**. Valuable functionalities, featuring halides, ketones, and esters were fully accepted, whereas a steroid derivative was smoothly converted without racemization by the photoinduced heterogeneous copper‐catalyzed C−H arylation.

**Scheme 2:**
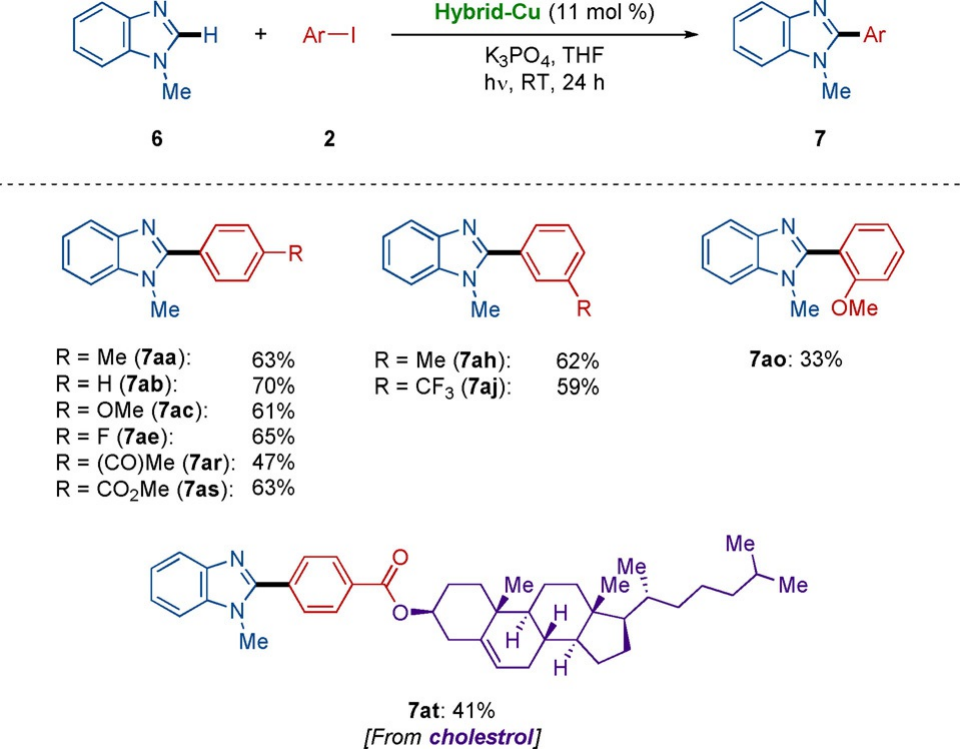
Photoinduced C−H arylation of imidazole derivatives **6** by using the hybrid copper catalyst.

Interestingly, the photoinduced heterogeneous C−H arylation manifold was not restricted to the functionalizations of aryl iodides **2**, but more cost‐effective aryl bromides **8** were also identified as suitable substrates under slightly modified conditions (Scheme 3).^19^ Thereby, aryl bromides **8** bearing electron‐rich and electron‐deficient functional groups were fully tolerated by the heterogeneous photocatalysis. The robustness of the photoinduced C−H arylation by the hybrid copper catalyst was reflected by mild and sustainable catalysis.

**Scheme 3:**
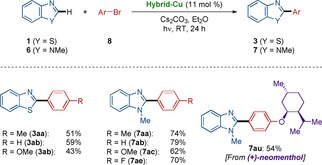
Photoinduced heterogeneous C−H arylation with aryl bromides **8**.

Thereafter, we performed mechanistic studies to rationalize the mode of action of the hybrid catalyst.^19^ To this end, an intermolecular competition experiment revealed that the electron‐deficient aryl iodide **2 e** underwent faster direct arylation, being suggestive of the oxidative addition the aryl halide onto the copper(I) intermediate to be rate‐determining (Scheme 4 a).^21^ Furthermore, we probed a SET‐type regime (SET=single‐electron transfer) by the representative radical scavenger 2,2,6,6‐tetramethylpiperidine *N*‐oxide (TEMPO), resulting in a significant inhibition of the photoinduced hybrid copper catalysis for C−H arylation (Scheme 4 b). A stoichiometric reaction with well‐defined copper(I) complex **8** further reflected the importance of the C−H arylation step in the photoinduced C−H arylation (Scheme 4 c).

**Scheme 4:**
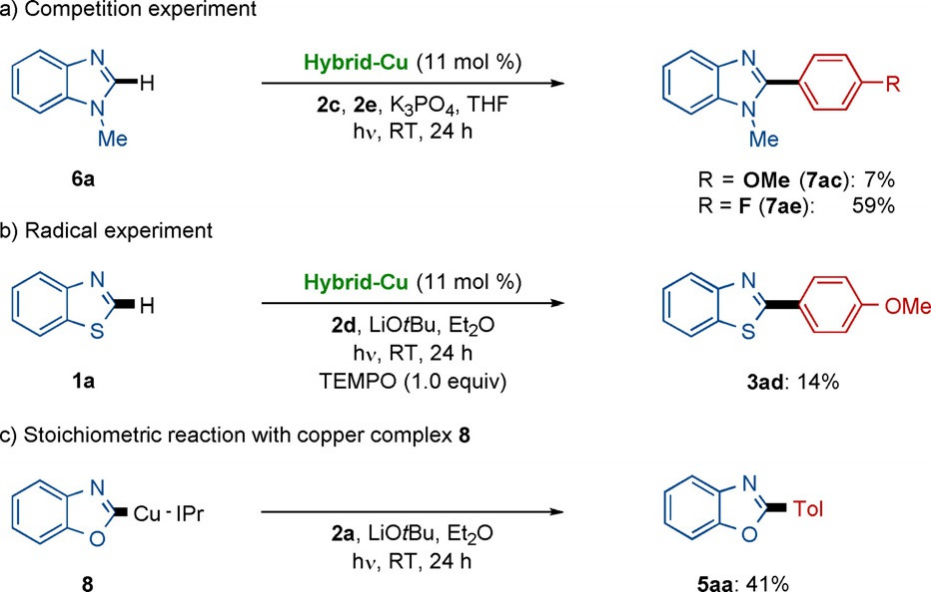
Key mechanistic studies. a) Competition experiment. b) Probing an SET‐type mechanism by TEMPO scavenger. c) Stoichiometric reaction with copper complex **8**.

Finally, we tested the photoinduced C−H arylation by on–off experiments, highlighting that the Hybrid‐Cu‐catalyzed C−H arylation is fully suppressed in the absence of light, and showing that constant irradiation is required for effective product formation (Scheme 5).^22^ A quantum yield of 12 % was determined, thus rendering a radical chain reaction unlikely to be of relevance.^19^ Additionally, we monitored the conversion profile of the photoinduced C−H arylation by Hybrid‐Cu catalyst.^19^

**Scheme 5:**
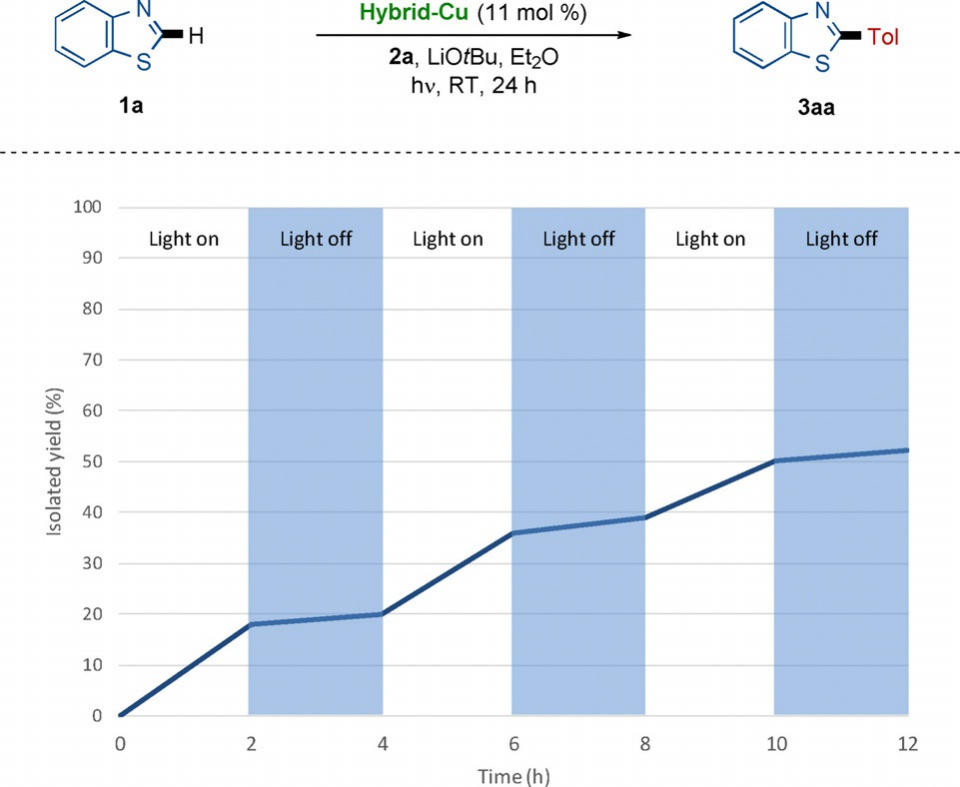
On–off light experiments for the photoinduced Hybrid‐Cu‐catalyzed C−H arylation.

Considering the efficacy of the versatile and robust photoinduced C−H arylation by the hybrid copper catalyst, we became intrigued to probe its potential reusable nature.^19^ We were hence delighted to observe that the Hybrid‐Cu featured reusability, providing facile access to the alkaloid natural product texamine **5 hb** (Scheme 6).^23^ It is worth noting that less than 4 ppm of copper was detected by detailed inductively coupled plasma optical emission spectrometry (ICP‐OES) analysis of the reaction mixture, reflecting negligible leaching of the transition metal.

**Scheme 6:**
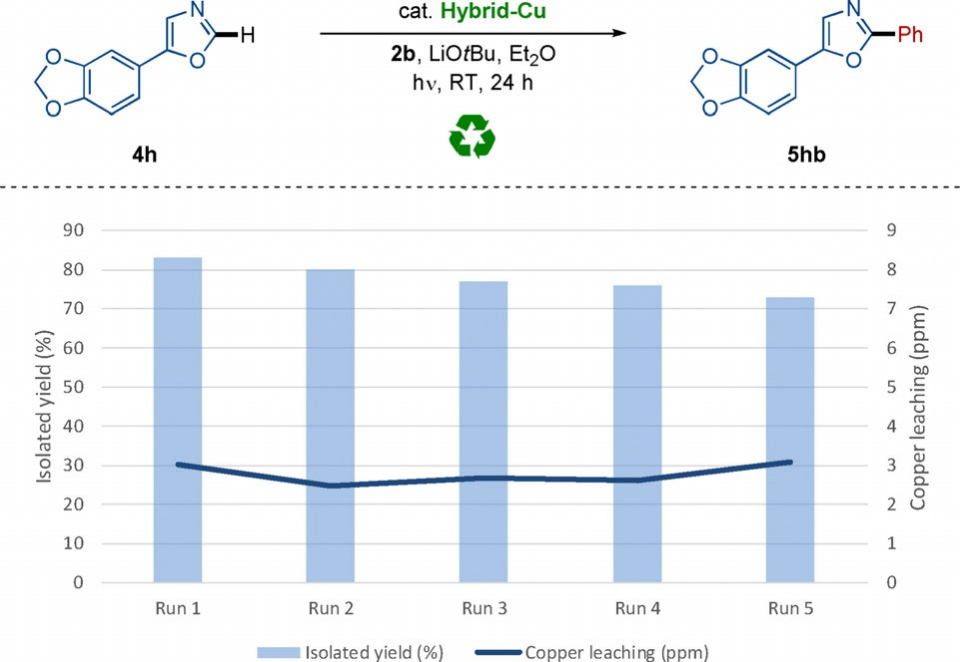
Reuse of the hybrid copper catalyst for photoinduced C−H arylation.

The robust reusability was probed in a gram‐scale reaction (Scheme 7 a). The heterogeneous nature of the hybrid photocatalyst was reflected by a filtration test and three‐phase reactions with immobilized substrates, rendering homogeneous catalysis highly unlikely to be operative (Scheme 7 b,c).

**Scheme 7:**
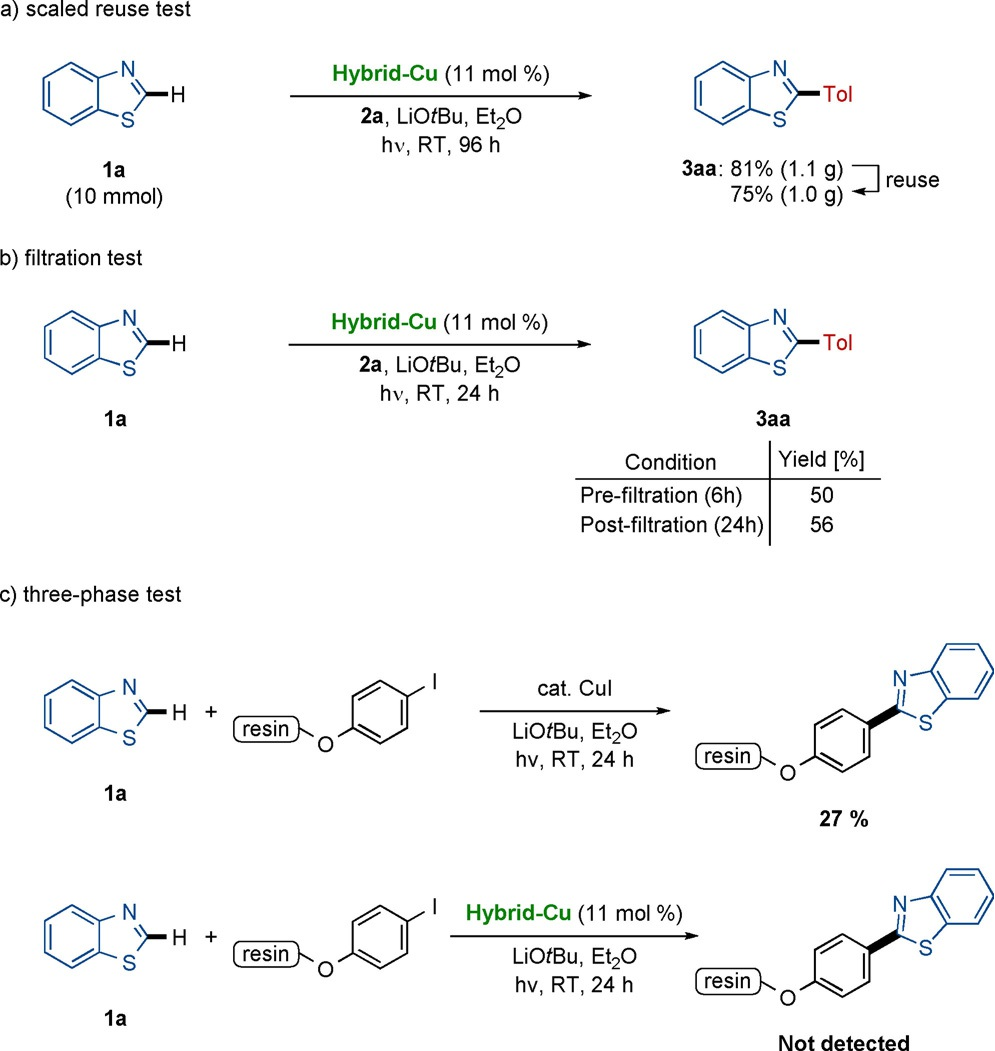
Heterogeneity tests. a) Scale‐up reuse test. b) Filtration test. c) Three‐phase test.

Given the unique features of the reusable hybrid copper catalyst, we intended to determine its morphological and atomic properties (Figure 2).^19^ To this end, we performed detailed transmission electron microscopy (TEM) and high‐resolution transmission electron microscopy (HRTEM) studies of SBA‐15, the Hybrid‐Support, Hybrid‐Cu and the reused Hybrid‐Cu. TEM images of the SBA‐15 and the Hybrid‐Support showed homogenously ordered mesoporous structures (Figure 2 a, I–VI). HRTEM and TEM images of Hybrid‐Cu and of the reused Hybrid‐Cu delineated highly ordered one‐dimensional mesoporous channels without morphological agglomeration (Figure 2 a, VII–XII),^24^ indicating outstanding reusability and stability of hybrid copper catalyst for photoinduced C−H arylation. Subsequently, X‐ray photoelectron spectroscopy (XPS) studies indicated that both Hybrid‐Cu and the reused Hybrid‐Cu are copper(I) species, based on Cu 2p_3/2_ and Cu LMM‐Auger peaks (Figure 2 b).^25^

**Figure 2:**
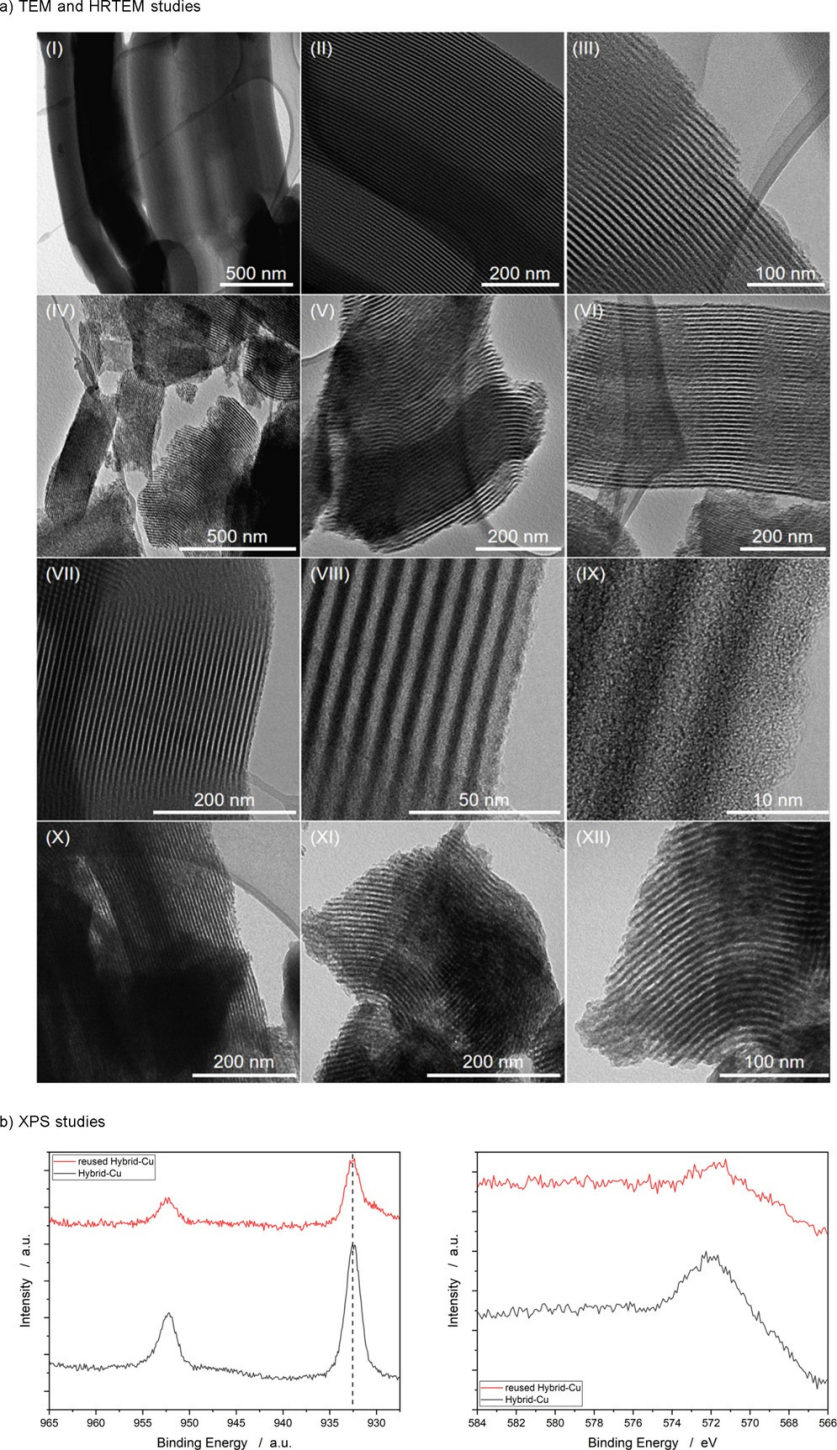
Physical and chemical analysis. a) TEM and HRTEM studies of SBA‐15 (I‐III), Hybrid‐Support (IV‐VI), Hybrid‐Cu (VII‐IX) and reused Hybrid‐Cu (X‐XII). All images are recorded with electron beam perpendicular to axis of periodic silica pores. b) XPS studies of Hybrid‐Cu and reused Hybrid‐Cu.

On the basis of our detailed mechanistic studies and the characterization of the hybrid copper catalyst, a plausible catalytic cycle for the photoinduced heterogeneous C−H arylation was proposed (Scheme 8). The mechanism rationale commences with hybrid copper(I) catalyst and benzothiazole, forming copper complex **A** by the aid of a base. Irradiation of copper complex **A** leads to a photoexcited state **B**, followed by a SET process involving aryl iodides **2** generating intermediate **C**. Finally, subsequent reductive elimination affords arylated product **3** and simultaneously regenerates hybrid copper(I) catalyst, as confirmed by XPS analysis.

**Scheme 8:**
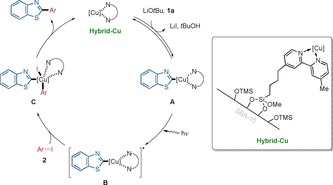
Proposed catalytic cycle.

In summary, we have reported a photoinduced C−H arylation by heterogeneous copper catalysis under exceedingly mild reaction conditions at room temperature.^26^ The modular hybrid copper catalyst featured remarkable catalytic power towards site‐selective C−H arylations with ample scope. The heterogeneous catalyst was reusable without significant loss of catalytic efficacy. Mechanistic studies showed strong evidence for photoinduced, excited‐state copper catalysis enabled by a reusable hybrid regime. Detailed microscopic and spectroscopic analysis illustrated excellent physical and chemical stability of the hybrid copper catalyst for photoinduced C−H arylation, providing a good agreement with experimental studies.

## Conflict of interest

The authors declare no conflict of interest.

## Supporting information

As a service to our authors and readers, this journal provides supporting information supplied by the authors. Such materials are peer reviewed and may be re‐organized for online delivery, but are not copy‐edited or typeset. Technical support issues arising from supporting information (other than missing files) should be addressed to the authors.

Supplementary
