# Supplementary material for: Photoinduced Heterogeneous C−H Arylation by a Reusable Hybrid Copper Catalyst
Source: Chemistry. 2020 Feb 25;26(16):3509–14. doi: 10.1002/chem.202000192 (PMC7155010; doi:10.1002/chem.202000192)

CHEMISTRY
A European Journal

Supporting Information

Photoinduced Heterogeneous C−H Arylation by a Reusable Hybrid Copper Catalyst

Isaac Choi,[a] Valentin Müller,[a] Gaurav Lole,[b] Robert Köhler,[c] Volker Karius,[d] Wolfgang Viöl,[c] Christian Jooss,[b] and Lutz Ackermann*[a, e]

chem_202000192_sm_miscellaneous_information.pdf

Table of Contents

General Remarks....................S1

General Procedures for the Preparation of Hybrid-Cu S2

General Procedures for Photo-induced Heterogeneous C–H Arylation S3

Optimization Studies....................S4

Determination of Catalyst Loading....................S7

Temperature Profile S7

Mechanistic Studies S8

Raw Data for Reaction Profile and On-Off Test....................S11

Experiments for the Proof of Heterogeneity....................S12

Microscopic Analysis....................S16

X-ray Photoelectron Spectroscopy S18

Characterization Data of Product **3**, **5**, and **7**....................S20

References....................S69

^{1}H NMR, ^{13}C NMR and ^{19}F NMR Spectra....................S71

General Remarks

Photo-induced heterogeneous C–H arylations by reusable hybrid-copper catalyst were performed under N_2 atmosphere in pre-dried quartz tubes using a Luzchem LZC-ICH2 photoreactor with 254 nm irradiation. Visible light-induced catalytic reactions were performed under N_2 atmosphere in pre-dried vials using blue LEDs (450-500 nm). The following starting materials were synthesized according to previously described methods: 5-arylthiazole **1g**[1] and 5-aryloxazoles **4f-4h**.[2] Other chemicals were obtained from commercial sources, and were used without further purification. Yields refer to isolated compounds, estimated to be > 95% pure as determined by ^{1}H NMR. TLC: Macherey-Nagel, TLC plates Alugram® Sil G/UV254. Detection under UV light at 254 nm. Chromatography: Separations were carried out on Merck Silica 60 (0.040-0.063 mm, 70-230 mesh ASTM). All IR spectra were recorded on a Bruker FT-IR Alpha device. MS: EI-MS: Finnigan MAT 95, 70 eV; DCI-MS: Finnigan MAT 95, 200 eV, reactant gas NH_3; ESI-MS: Finnigan LCQ. High resolution mass spectrometry (HR-MS): APEX IV 7T FTICR, Bruker Daltonic. M.p.: Stuart® SMP3 melting point apparatus, all values are uncorrected. ^{1}H, ^{13}C, and ^{19}F NMR spectra were recorded on Varian 300 MHz, Varian 500 MHz, and Bruker 400 MHz spectrometers in $CDCl_3$ solutions, chemical shifts (δ) are given in ppm. The chemical surface composition was investigated by X-ray photoelectron spectroscopy (XPS). The XPS data were recorded using a monochromatic Al-Kα radiation with a photon energy of 1486.6 eV (PHI 5000 Versa Probe II, ULVAC-PHI, Osaka, Japan). The detector resolution measured at the Ag 3d5/2 peak is 0.6 eV at a pass energy of 23.5 eV. Survey as well as carbon (C 1s), copper (Cu 2p3), copper auger (Cu LMM) and sulphur (S 2s) detail spectra of the samples were recorded in X-ray scanning mode. With a spot size of 100 µm, X-Ray power of 100 W continuously scanned over a length and wide of 1300 µm and 200 µm. The used pass energy for the detail spectra is 23.5 eV. To the identification of the peaks, the spectra were shifted to the main C 1s peak at 284.8 eV. To avoid charging effects, the measurements were carried out by neutralizing sample charging.

General Procedures for the Preparation of Hybrid-Cu

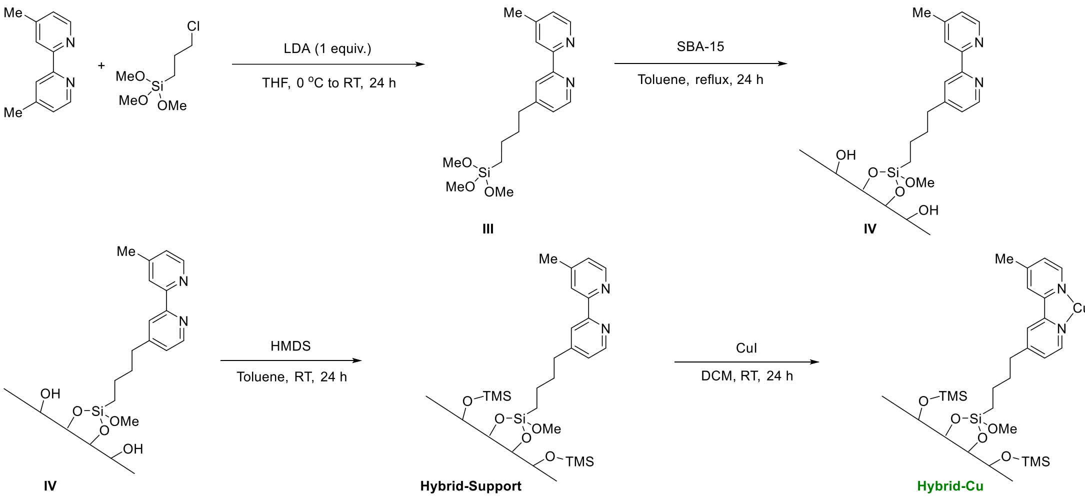

The Procedure for the synthesis of Hybrid-Cu is modified from a published paper.[3]

Linker modification: To a solution of DIPA (2.1 mL,15 mmol) in 40 mL of dried THF (50 mL) was added *n*-BuLi (2.1 M, 6.3 mL, 13.2 mmol) dropwise at 0 °C under N_2, *in-situ* generating LDA. After the reaction mixture was stirred for 1 h, a solution of 4,4-dimethyl-2,2′-bipyridine (13.2 mmol, 2.42 g) in dried THF (100 mL) was slowly added under N_2, then color was changed to dark brown. After stirring the reaction mixture for 1 h, a solution of (3-chloropropyl)trimethoxylsilane (13.2 mmol, 2.41 mL) in 10 mL of THF was slowly added at 0 °C under N_2. The solution was further stirred for an additional 2 h. The reaction mixture was warmed to room temperature and stirred for 24 h. After the reaction was quenched with two drops of acetone, the remaining solvent was removed and then dried *in vacuo* at room temperature for 24 h, affording **III**.

Grafting: A suspension of SBA-15 (5.0 g) and **III** in dried toluene were refluxed under N_2 for 24 h. The powder was filtered off, washed with toluene, *n*-hexane, and DCM (each 50 mL), and dried *in vacuo* at 100 °C for 24 h, yielding **IV**.

End-Capping: Hexamethyldisilazane (20 mL) and **IV** in dried toluene (50 mL) was stirred at room temperature for 24 h. The powder was filtered off, washed with toluene, *n*-hexane, and DCM (each 50 mL), and dried *in vacuo* at 100 °C for 24 h, providing **Hybrid-Support.**

Metallation: A suspension of CuI (1.2 g, 6.0 mmol) and **Hybrid-Support** were stirred in DCM at room temperature for 24 h. The powder filtered off, washed with DCM, MeCN, and MeOH (each 50 mL), and dried *in vacuo* at 100 °C for 24 h, finally affording **Hybrid-Cu**.

General Procedures for Photo-induced Heterogeneous C–H Arylation

1) General procedure 1 (**GP1**) for photo-induced C–H arylation of benzoazoles and azoles by Hybrid-Cu:

   To a pre-dried 10 mL quartz tube was added heterocycles **1** or **4** (0.25 mmol), aryl iodide **2** (1.25 mmol), Hybrid-Cu (30 mg, 11 mol %), LiO*t*Bu (60 mg, 0.75 mmol), and Et_2O (0.5 mL) under N_2 atmosphere. The tube was sealed and stirred under 254 nm irradiation in a Luzchem LZC-ICH2 photoreactor at ambient temperature for 24 h. The temperature was determined to be 30 °C in the reaction mixture. Afterwards, the solvent was removed under reduced pressure. Purification by column chromatography on silica gel (*n*-Hexane/EtOAc) afforded the desired products **3** or **5** respectively.

2) General procedure 2 (**GP2**) for photo-induced C–H arylation of *N*-methyl benzimidazole by Hybrid-Cu:

   To a pre-dried 10 mL quartz tube was added *N*-methyl benzimidazole **6a** (0.25 mmol), aryl iodide **2** (1.25 mmol), Hybrid-Cu (30 mg, 11 mol %), K_3PO_4 (159 mg, 0.75 mmol), and THF (0.5 mL) under N_2 atmosphere. The tube was sealed and stirred under 254 nm irradiation in a Luzchem LZC-ICH2 photoreactor at ambient temperature for 24 h. The temperature was determined to be 30 °C in the reaction mixture. Afterwards, the solvent was removed under reduced pressure. Purification by column chromatography on silica gel (*n*-Hexane/EtOAc) afforded the desired products **7.**

3) General procedure 3 (**GP3**) for photo-induced C–H arylation of heterocycles with aryl bromide by Hybrid-Cu:

   To a pre-dried 10 mL quartz tube was added heterocycles **1** or **6** (0.25 mmol), aryl bromide **8** (1.25 mmol), Hybrid-Cu (30 mg, 11 mol %), Cs_2CO_3 (244 mg, 0.75 mmol), and Et_2O (0.5 mL) under N_2 atmosphere. The tube was sealed and stirred under 254 nm irradiation in a Luzchem LZC-ICH2 photoreactor at ambient temperature for 24 h. The temperature was determined to be 30 °C in the reaction mixture. Afterwards, the solvent was removed under reduced pressure. Purification by column chromatography on silica gel (*n*-Hexane/EtOAc) afforded the desired products **3** or **7**.

Optimization Studies

Table S1. Optimization studies for the photo-induced heterogeneous C–H arylation by hybrid-copper catalyst.

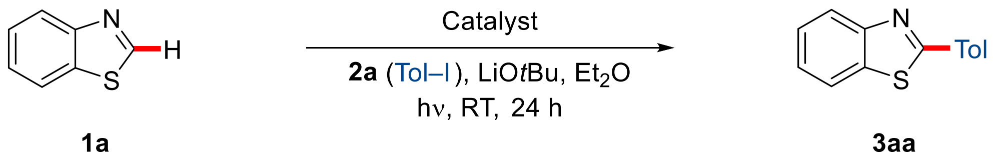

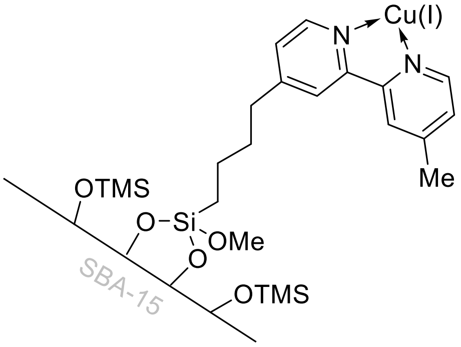

Hybrid-Cu

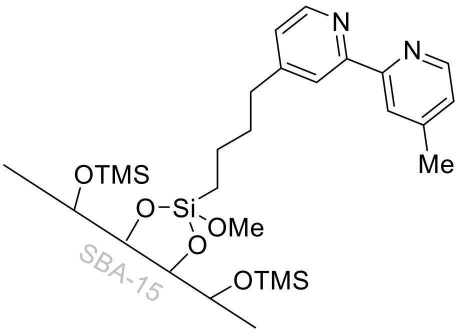

Hybrid-Support

Entry	Catalyst	Yield (%)[a]
1	Hybrid-Cu	93[b,c]
2	Reuse from entry **1**	85[b,c]
3	CuI	58[b]
4	Cu-DMBP	60[b]
5	CuI + DMBP	50[b]
6	CuI + Hybrid-support	36[b,d]
7	--	trace
8	Hybrid support	trace
9	Hybrid-Cu	trace[e]

[a] Reaction conditions: **1a** (0.25 mmol), **2a** (1.25 mmol), catalyst (11 mol %), LiO*t*Bu (0.75 mmol), Et_2O (1.0 mL), 254 nm, RT, 24 h, isolated yield. [b] Et_2O (0.50 mL). [c] Average yield over two runs. [d] 25 mg of Hybrid support **4**. [e] In the dark. DMBP = 4,4-Dimethyl-2,2-dipyridyl. For determination of catalyst loading and the measurement of reaction temperature, see S7.

Table S2. Screening for the photo-induced C–H arylation of *N*-methyl benzimidazoles by hybrid-copper catalyst.

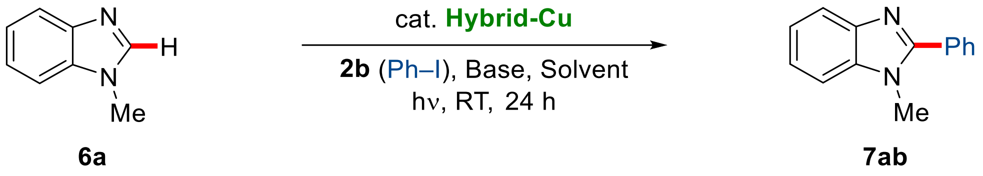

Entry	Base	Solvent	Yield (%)[a]
1	LiO*t*Bu	DMF	0
2	KO*t*Bu	DMF	0
3	K_2CO_3	DMF	5
4	K_3PO_4	DMF	12
5	K_3PO_4	THF	70 (61)[b,c]
6	K_3PO_4	MeCN	trace
7	K_3PO_4	Et_2O	trace
8	K_3PO_4	1,4-dioxane	trace

[a] Reaction conditions: **1a** (0.25 mmol), **2a** (1.25 mmol), catalyst (11 mol %), base (0.75 mmol), solvent (1.0 mL), 254 nm, RT, 24 h, isolated yield. [b] Average yield over two runs. [c] the yield in parenthesis is the result with reused Hybrid-Cu.

Table S3. Screening for the photo-induced C–H arylation with aryl bromides by hybrid-copper catalyst.

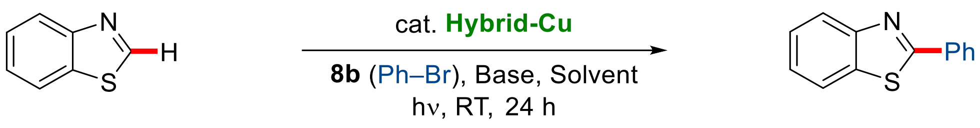

1a **3ab**

Entry	Base	Solvent	Yield (%)[a]
1	LiO*t*Bu	Et_2O	0
2	KO*t*Bu	Et_2O	0
3	Li_2CO_3	Et_2O	trace
4	Na_2CO_3	Et_2O	trace
5	K_2CO_3	Et_2O	21
6	Cs_2CO_3	Et_2O	59 (47)[b,c]

[a] Reaction conditions: **1a** (0.25 mmol), **2a** (1.25 mmol), catalyst (11 mol %), base (0.75 mmol), solvent (1.0 mL), 254 nm, RT, 24 h, isolated yield. [b] Average yield over two runs. [c] the yield in parenthesis is the result with reused Hybrid-Cu.

Determination of Catalyst Loading

Amount of Catalyst $\times$ ICP-OES result $\div$ Molcular weight of copper $\div$ amount of substrates $\times$ 100 mol %

$$= 30\text{ mg} \times \frac{58300\text{ mg}}{1{,}000{,}000\text{ mg}} \times \frac{1}{63.546\text{ mg/mmol}} \times \frac{1}{0.25\text{ mmol}} \times 100\text{ mol }\% = \mathbf{11\ mol}\ \%$$

Temperature Profile

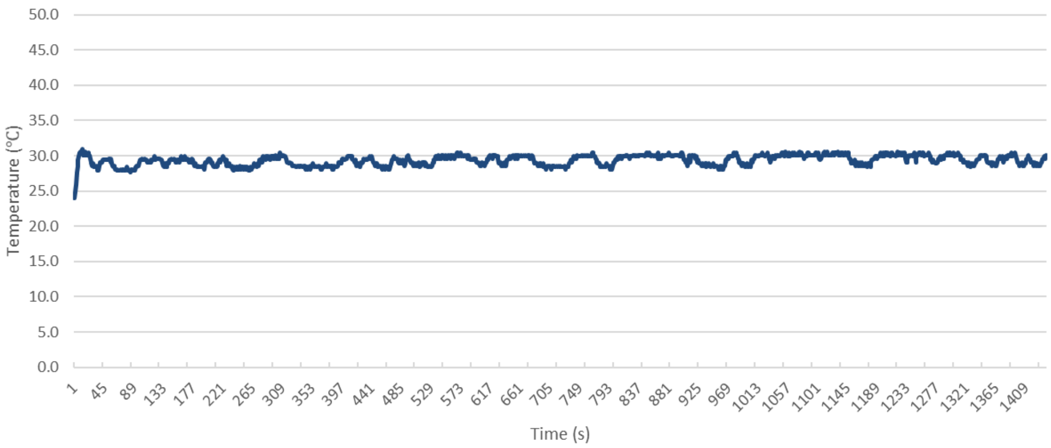

Mechanistic Studies

1) Competition experiment

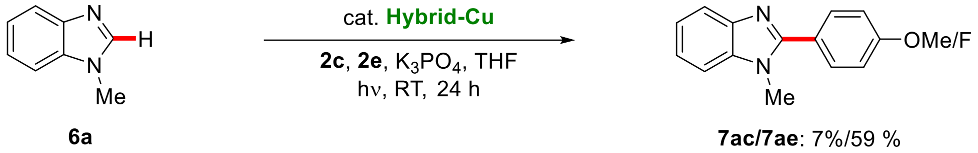

GP1 was followed using 1-methyl-1*H*-benzo[*d*]imidazole (**6a**) (33 mg, 0.25 mmol), 4-iodoanisole (**2c**) (293 mg, 1.25 mmol), 1-fluoro-4-iodobenzene (**2e**) (278 mg, 1.25 mmol), Hybrid-Cu (30 mg, 11 mol %) and K_3PO_4 (159 mg, 0.75 mmol) in THF (1.0 mL) under N_2. After 24 h the solvent was removed under reduced pressure. Purification by column chromatography on silica gel (*n*-Hexane/EtOAc = 6/1 to 1/1) afforded the desired products **7ac** (4.2 mg, 7%) and **7ae** (33.3 mg, 59%) respectively.

2) Radical experiments

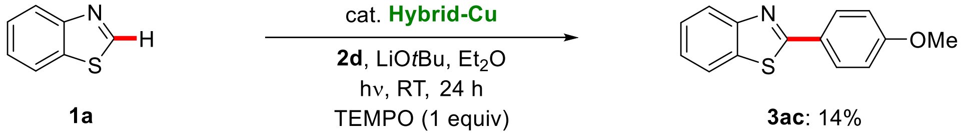

GP1 was followed using benzothiazole (**1a**) (34 mg, 0.25 mmol), 4-iodoanisole (**2c**) (293 mg, 1.25 mmol), Hybrid-Cu (30 mg, 11 mol %), LiO*t*Bu (60 mg, 0.75 mmol) and TEMPO (39 mg, 0.25 mmol) in Et_2O (0.5 mL). Purification by column chromatography on silica gel (*n*-Hexane/EtOAc: 70/1) yielded **3ac** (8.3 mg, 14%) as a light yellow solid.

3) Stoichiometric reaction with copper complex **8**

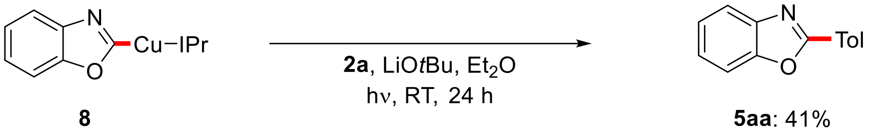

To a pre-dried 10 mL quartz tube was added copper complex **8** (142 mg, 0.25 mmol), 4-iodotoluene (**2a**) (273 mg, 1.25 mmol), and LiO*t*Bu (60 mg, 0.75 mmol) in Et_2O (0.5 mL) under N_2 atmosphere. Purification by column chromatography on silica gel (*n*-hexane/EtOAc: 70/1) yielded **5aa** (21.4 mg, 41%) as a light yellow solid.

4) H/D exchange experiments

a. Photo-excited condition

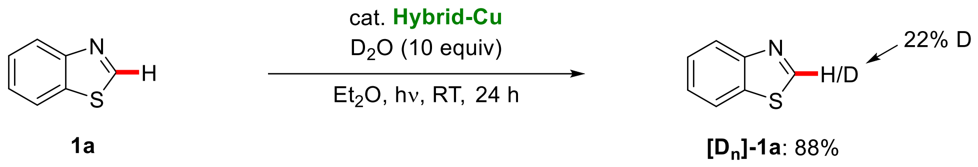

GP1 was followed using benzothiazole (**1a**) (34 mg, 0.25 mmol), Hybrid-Cu (30 mg, 11 mol %), D_2O (50 mg, 2.5 mmol) in Et_2O (0.5 mL). Purification by column chromatography on silica gel (*n*-Hexane/EtOAc: 10/1) yielded **[D_n]-1a**, determining H/D scrambling by ^{1}H NMR.

b. In dark condition

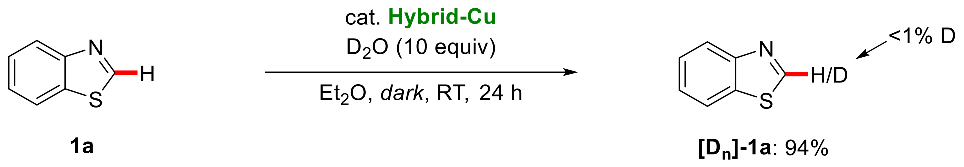

GP1 was followed using benzothiazole (**1a**) (34 mg, 0.25 mmol), Hybrid-Cu (30 mg, 11 mol %), D_2O (50 mg, 2.5 mmol) in Et_2O (0.5 mL). The tube was covered with aluminum foil and stirred under 254 nm irradiation in a Luzchem LZC-ICH2 photoreactor at ambient temperature for 24 h. Purification by column chromatography on silica gel (*n*-Hexane/EtOAc: 10/1) yielded **[D_n]-1a**, determining H/D scrambling by ^{1}H NMR.

5) Determination of quantum yield[5]

a. Preparation of potassium ferrioxalate solution

295 mg of solid potassium ferrioxalate, 140 μL H_2SO_4 were diluted with H_2O to a final volume of 50 mL.

b. Preparation of buffer solution

4.95 g NaOAc and 1 mL H_2SO_4 were diluted with H_2O to a final volume of 100 mL. Using the same setup as for catalytic reactions 2 mL of the potassium ferrioxalate solution was irradiated for 20 sec. The sample solution was added to 4 mL of the buffer solution containing 2 mg 1,10-phenanthroline. The solution was diluted with H_2O to a final volume of 10 mL. Subsequently, the absorbance of this solution was determined at 510

nm. The same procedure was followed for a nonirradiated sample.

c. Calculation number of photons

A: Abs of Fe^{2+} (at 510 nm) = 2.2385 (after irradiation of 20 sec)

A_0: Abs of Fe^{2+} (at 510 nm) = 0.0606 (no irradiation)

$$[\mathrm{Fe}^{2+}] = \frac{(\mathrm{A} - \mathrm{A}_0)}{\epsilon_{510\ \mathrm{nm}} \times \mathrm{l}}$$

$$[\mathrm{Fe}^{2+}] = \frac{(2.2385 - 0.0606)}{11100\ \mathrm{M}^{-1}\mathrm{cm}^{-1} \times 1\ \mathrm{cm}} = 1.9621 \cdot 10^{-4}\ \mathrm{M}$$

$$n_{(\mathrm{Fe}^{2+})} = 1.9621 \cdot 10^{-4}\ \mathrm{M}\ \times 0.010\ \mathrm{L} = 1.9621 \cdot 10^{-6}\ \mathrm{mol}$$

with quantum yield (Φ) of 1.38 for the absorption of Fe^{2+} at 254 nm:[6]

$$n_{(\mathrm{photons})} = \frac{n_{(\mathrm{Fe}^{2+})}}{\Phi} = \frac{1.9621 \cdot 10^{-6}\ \mathrm{mol}}{1.38} = 1.4218 \cdot 10^{-6}\ \mathrm{mol}$$

$$n_{(\mathrm{photons/s})} = \frac{n_{(\mathrm{photons})}}{t_{\mathrm{irradiation}}} = \frac{1.4218 \cdot 10^{-6}\ \mathrm{mol}}{20\ s} = 7.1090 \cdot 10^{-8}\ \mathrm{mol\ s}^{-1}$$

$$\text{Quantum Yield} = \frac{n_{\mathrm{product/s}}}{n_{(\mathrm{photons/s})}} = \frac{8.4604 \cdot 10^{-9}\ \mathrm{mol\ s}^{-1}}{7.1090 \cdot 10^{-8} \mathrm{mol\ s}^{-1}} = 0.12 = 12\%$$

d. Determination of the initial rate

Product formation was monitored by ^1H NMR using 1,3,5-Trimethoxy benzene as internal standard.

Reaction time (s)	**3aa** (%)	*n*(**3aa**) (mol)
1200	0.57	$1.425 \cdot 10^{-6}$
2400	1.67	$4.175 \cdot 10^{-6}$
3600	6.25	$1.563 \cdot 10^{-5}$
4800	9.13	$2.283 \cdot 10^{-5}$
6000	14.2	$3.550 \cdot 10^{-5}$
7200	18.0	$4.510 \cdot 10^{-5}$

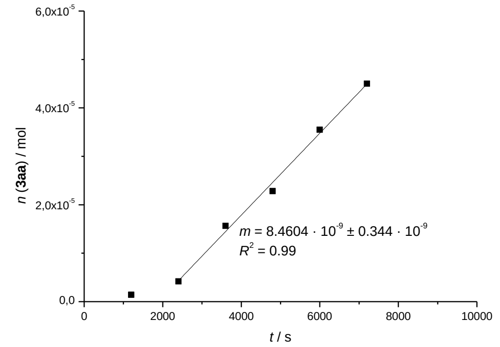

Raw Data for Reaction Profile and On-Off Test

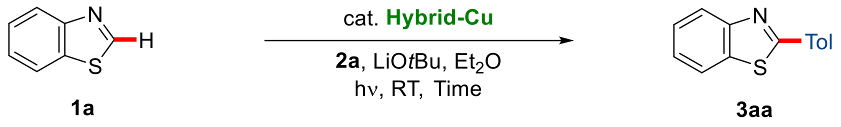

1) Reaction profile during the whole reaction time

Time (h)	0	2	4	6	8	10	12	14	16	18	20	22	24
Yield (%)[a]	0	18	37	50	61	73	80	87	89	91	92	93	93

[a] isolated yield

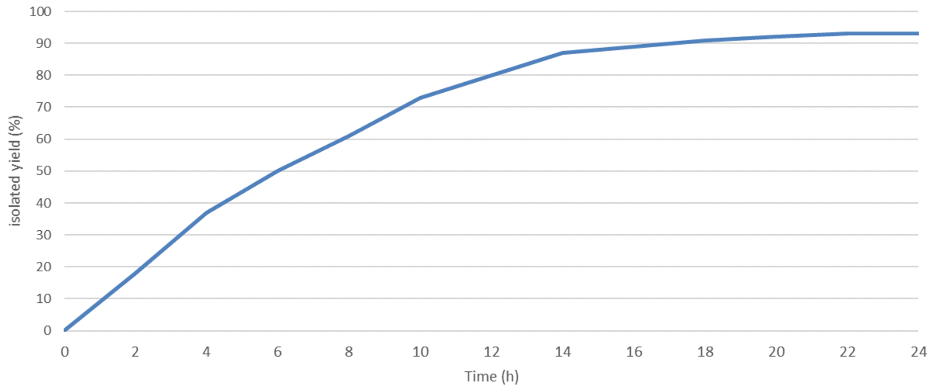

2) On-Off test for initial 12 h

Light	On	Off	On	Off	On	Off
Accumulated time (h)	2	4	6	8	10	12
Yield (%)[a]	18	20	36	39	50	52

[a] isolated yield

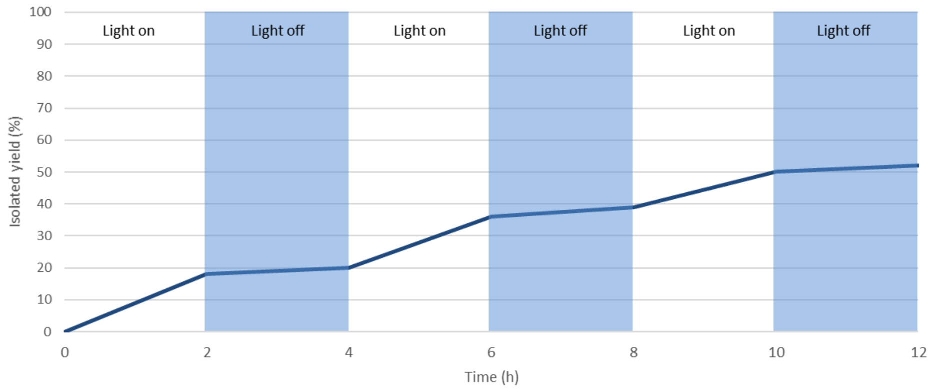

Experiments for the Proof of Heterogeneity

1) Reuse test

   GP1 was followed using 5-(benzo[*d*][1,3]dioxol-5-yl)oxazole **4h** (47 mg, 0.25 mmol), iodobenzene **2b** (255 mg, 1.25 mmol), Hybrid-Cu (60 mg, 22 mol %) and LiO*t*Bu (60 mg, 0.75 mmol) in Et_2O (1.0 mL). After 24 h the reaction tube was transferred to a glovebox. Hybrid-Cu was carefully filtered through branched filter (Por. 3) with Et_2O and DCM (each 10 mL). Filtered Hybrid-Cu was dried *in vacuo* and used for next run. Filtrate was used for either obtaining desired product **5hb** (texamine) by column chromatography on silica or measuring metal leaching by ICP-AES. The ICP-AES analysis data is shown in the table below.

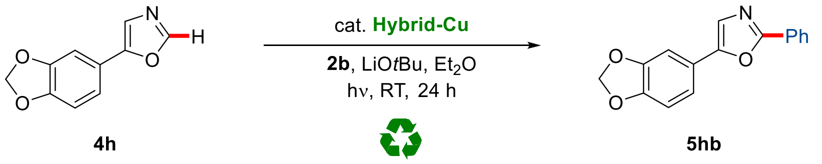

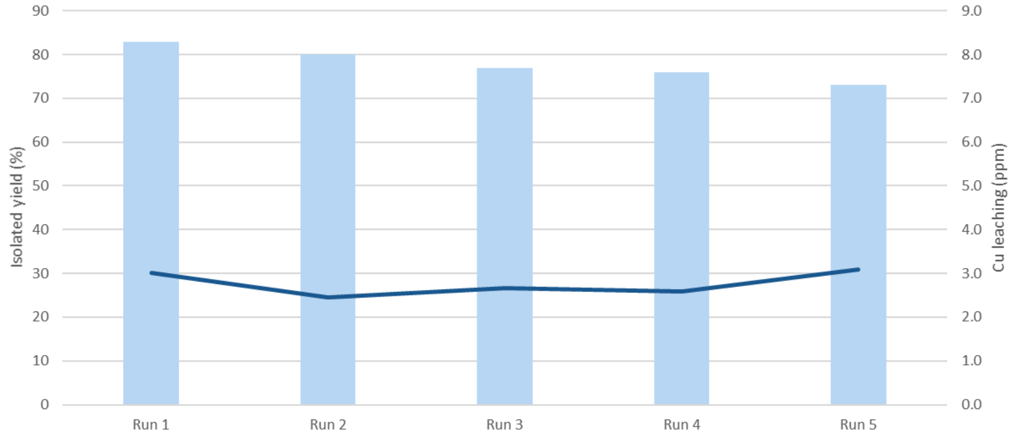

Run	Copper in solution (ppm)
1st Run	3.01
2nd Run	2.46
3rd Run	2.66
4th Run	2.59
5th Run	3.08

2) Scaled reuse test

GP1 was followed using benzothiazole (**1a**) (1.36 g, 10 mmol), 4-iodotoluene (**2a**) (10.92 g, 50 mmol), Hybrid-Cu (1.20 g, 11 mol %) and LiO*t*Bu (2.40 g, 30 mmol) in Et_2O (10 mL). After 24 h the reaction tube was transferred to a glovebox. Hybrid-Cu was carefully filtered through branched filter (Por. 3) with Et_2O and DCM (each 20 mL). Filtered Hybrid-Cu was dried *in vacuo* and used for the second run. The solvent of filtrate was removed under reduced pressure. Purification by column chromatography on silica gel (*n*-hexane/EtOAc: 70/1) yielded **3aa** (first run: 1.09 g, 81%, second run: 1.01 g, 75%) as a light yellow solid.

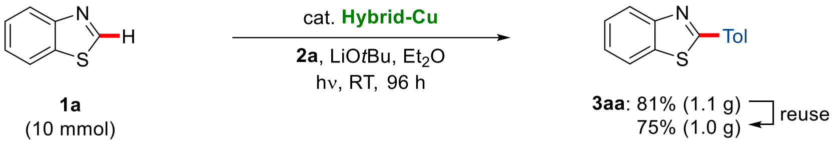

3) Filtration test

GP1 was followed using benzothiazole **1a** (34 mg, 0.25 mmol), 4-iodotoluene **2a** (273 mg, 1.25 mmol), Hybrid-Cu (30 mg, 11 mol %) and LiO*t*Bu (60 mg, 0.75 mmol) in Et_2O (0.5 mL). After 6 hours, reaction tube was transferred to the Glove box. Hybrid-Cu was carefully filtered over Celite® with a branched filter (Por. 3). Filtrate was directly collected to another pre-dried 10 mL quartz tube and then sealed with septum. The sealed tube was transferred to the Luzchem LZC-ICH2 photoreactor and the reaction continued for 18 h. Afterwards, the solvent was removed under reduced pressure. Purification by column chromatography on silica gel (*n*-Hexane/EtOAc) afforded the desired product **3aa**. The result shown below demonstrated that the reaction was stopped by removing Hybrid-Cu. The result data is shown below.

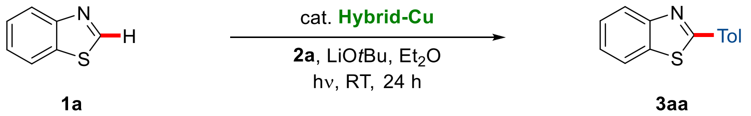

	Yield (%)[a] (6 h)	Yield (%)[a] (24 h)
Control reaction	50	93
Filtration (25 °C)	50	56

[a] isolated yield

4) Three-phase test

a. Synthesis of Resin and reaction procedure[4]

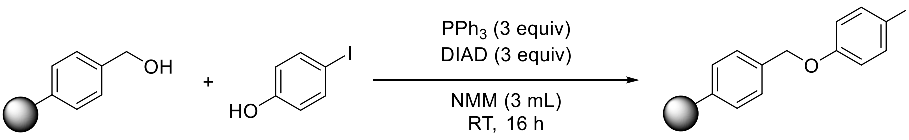

(1)

Resin **1**

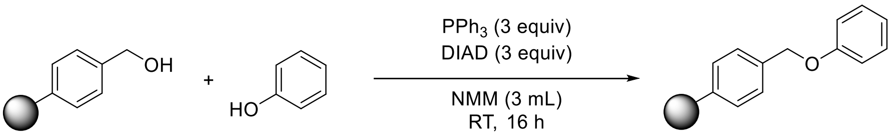

(2)

Resin **2**

Wang-resin (100-200 mesh, 1.0-1.5 mmol/g OH loading, 1% crosslinked with divinylbenzene) was washed with DCM and EtOH and dried *in vacuo* prior to use. To a suspension of Wang-resin (2.0 g, 2.0 mmol, 1.0 equiv), PPh_3 (1.6 g, 6 mmol, 3.0 equiv) and 4-iodophenol (1.3 g, 6.0 mmol, 3.0 equiv) or phenol (565 mg, 6.0 mmol, 3.0 equiv) in 4-Methylmorpholine (12 mL), diisopropyl azodicarboxylate (1.2 g, 6.0 mmol, 3.0 equiv) was added dropwise at room temperature. The reaction mixture was sonicated for 1 h prior to stirring at room temperature for 16 h. The reaction mixture was filtered, the residue was washed with THF, DCM, MeOH, H_2O, MeOH, and DCM (each 30 mL) and dried *in vacuo*. To determine the loading of Resin **1**, 50 mg of the Wang-resin was stirred in a TFA/DCM-mixture (4 mL, 3:1) at room temperature for 3 h. The reaction mixture was filtered, the residue washed with DCM (20 mL) and sat. aqueous $NaHCO_3$ (30 mL). The combined aqueous layer was extracted with DCM and the combined organic layer was dried over Na_2SO_4 and concentrated under reduced pressure. GC-MS was used for determination of loading with 1,3,5-trimethoxybenzene as an internal standard, providing the result; 0.5 mmol/g. **GP1** was followed using benzothiazole (**1a**) (34 mg, 0.25 mmol), 4-iodoanisole (**2c**) (292 mg, 1.25 mmol), Hybrid-Cu (30 mg, 11 mol %) and LiO*t*Bu (60 mg, 0.75 mmol) in Et_2O (2.0 mL), otherwise mentioned in the schemes below. The formation of product on resin was determined by GC-MS with 1,3,5-trimethoxybenzene as an internal standard.

b. Standard reaction in presence of Resin **2**

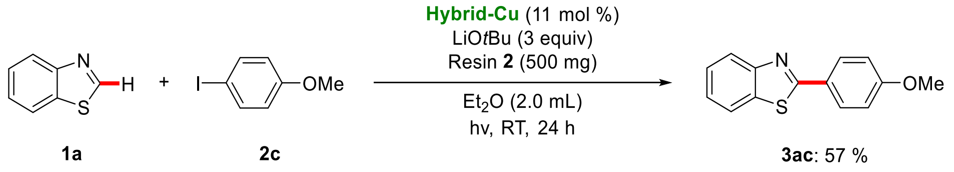

Result: The product is formed in a solution and the reaction was slightly interfered by the resin.

c. Reaction with Resin **1** instead of **2c**

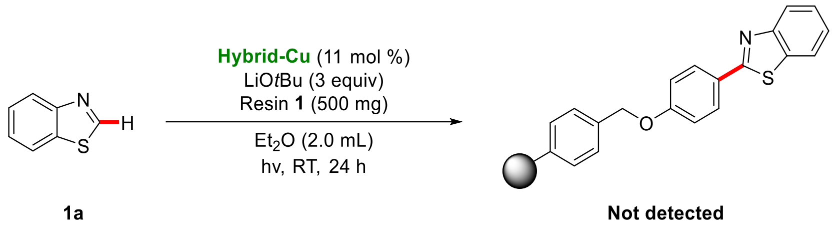

Result: Product formation on the resin was not observed.

d. Reaction with CuI instead of Hybrid-Cu

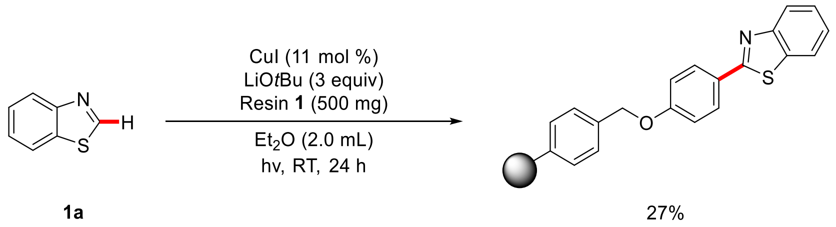

Result: Product formation on the resin was observed.

Microscopic Analysis

1) Materials and methods

Aqueous suspensions of the catalysts were prepared using THF solution and dried a small drop of the diluted suspension on a TEM copper grid coated with a carbon film (200 mesh). The HRTEM studies of SBA-15, Hybrid-Support and Hybrid-Cu have been investigated by using FEI/Philips CM-12T TEM and an aberration corrected Titan 80-300 environmental microscope (Thermo Scientific™, former FEI, Hillsboro, Oregon, USA) operated at 120 kV and 300 kV respectively.

2) Result and discussion

a. Transmission electron microscopy analysis of SBA-15 (I-III), Hybrid-Support (IV-VI). All images are recorded with electron beam perpendicular to axis of periodic silica pores.

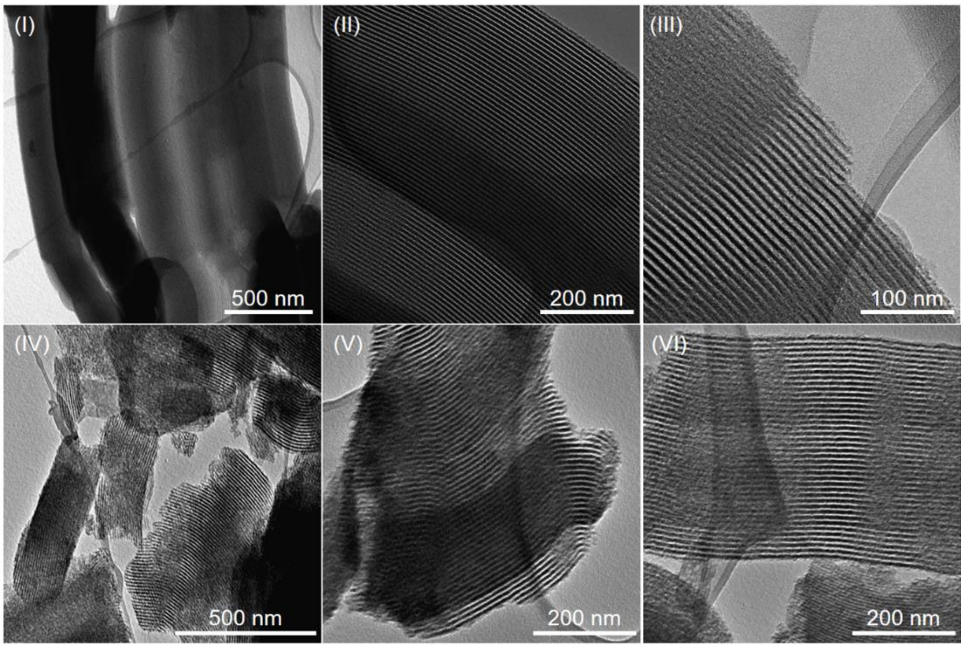

b. High-resolution transmission electron microscopy analysis of Hybrid-Cu (VII-IX) and reused Hybrid-Cu (X-XII). All images are recorded with electron beam perpendicular to axis of periodic silica pores.

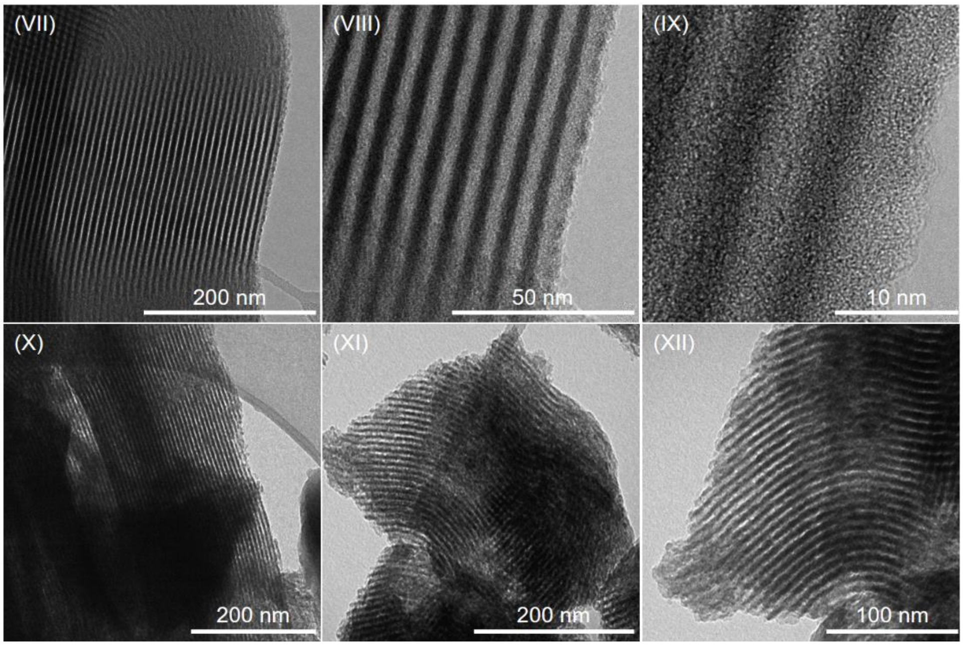

X-ray Photoelectron Spectroscopy

1) Material and methods

The chemical surface composition was investigated by X-ray photoelectron spectroscopy (XPS). The XPS data were recorded using a monochromatic Al-Kα radiation with a photon energy of 1486.6 eV (PHI 5000 Versa Probe II, ULVAC-PHI, Osaka, Japan). The detector resolution measured at the Ag 3d5/2 peak is 0.6 eV at a pass energy of 23.5 eV. Survey as well as carbon (C 1s), copper (Cu 2p3), copper auger (Cu LMM) and sulphur (S 2s) detail spectra of the samples were recorded in X-ray scanning mode. With a spot size of 100 µm, a X-Ray power of 100 W was continuously scanning over a length and wide of 1300 µm and 200 µm. The used pass energy for the detail spectra is 23.5 eV. For the identification of the peaks, the spectra were shifted to the main C 1s peak at 284.8 eV. To avoid charging effects, the measurements were carried out by neutralizing sample charging.

2) Results and discussion

Survey spectra of the hybrid copper catalyst is shown below.

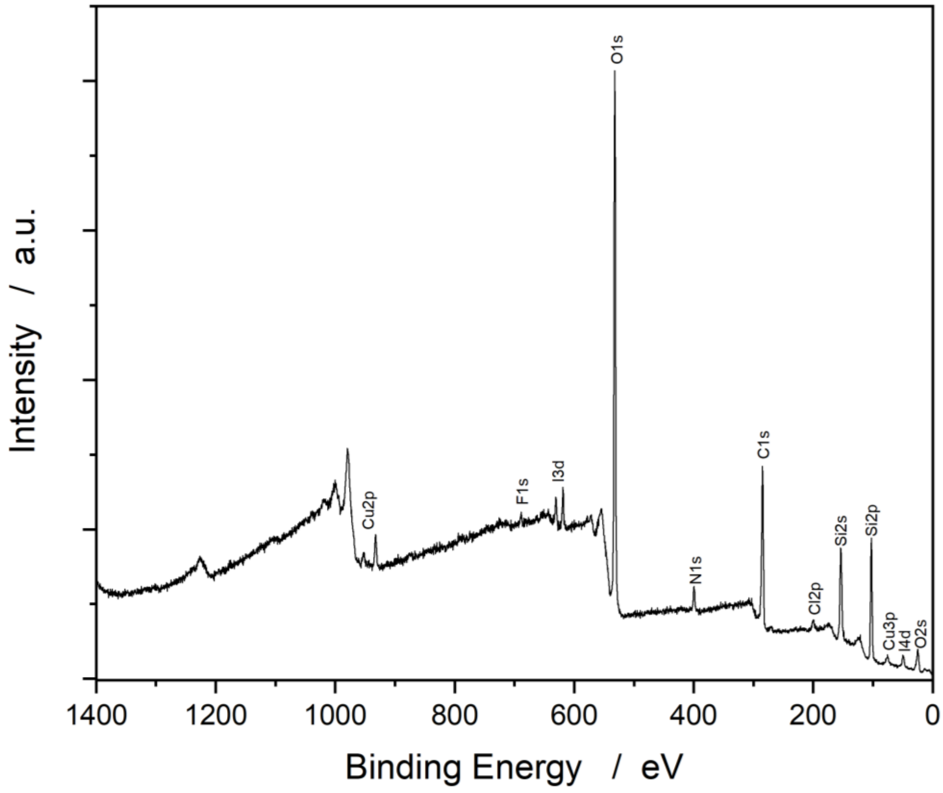

The Cu 2p3 peak was investigated for the determination of the oxidation state of Hybrid-Cu. The peaks of the Hybrid-Cu and the reused Hybrid-Cu are located at 932.6 eV. These preliminary results indicated the presence of Cu(0) or Cu(I).[7] For the distinction between Cu(0) and Cu(I), the Cu LMM-Auger peak was analyzed, showing comparable results to published paper.[8] The structure of the measured Cu LMM peak indicates Cu(I).

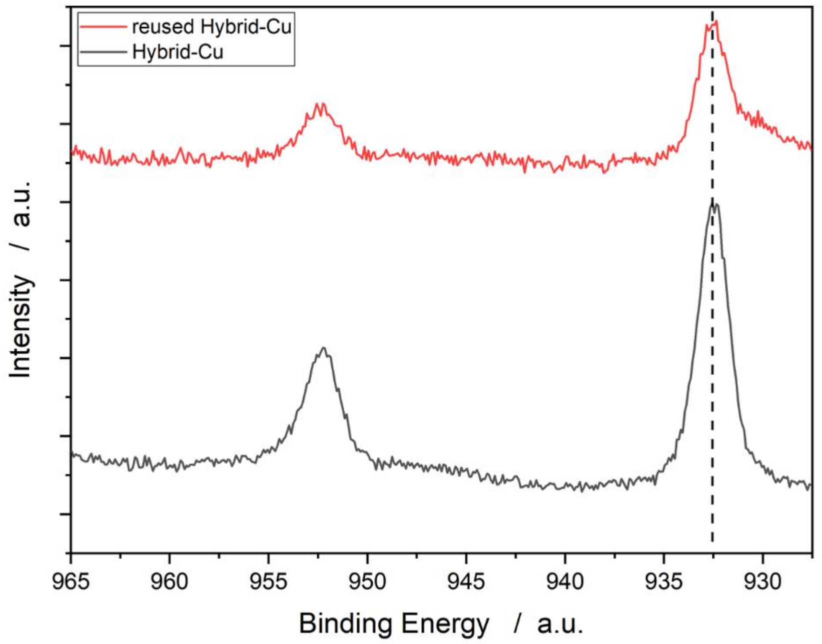

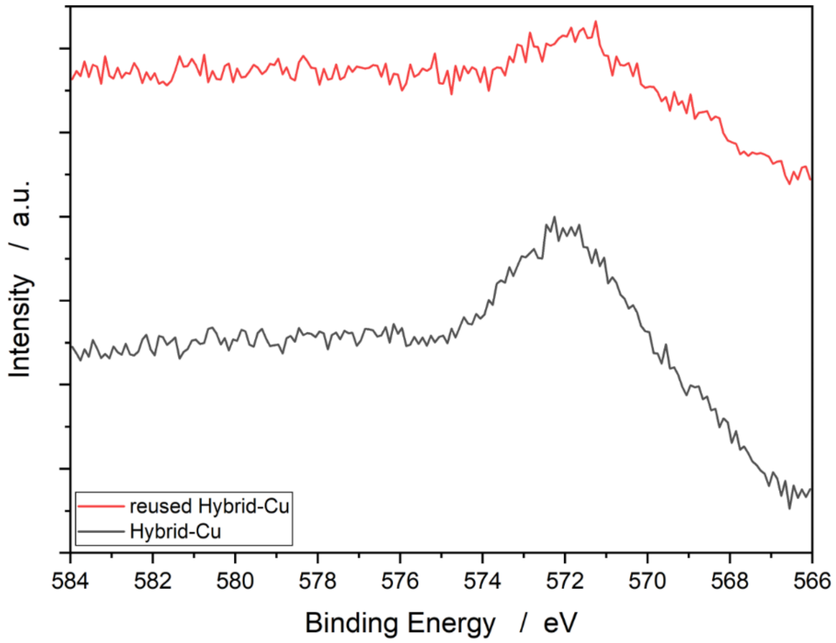

Characterization Data of Product 3, 5, and 7

2-(*p*-Tolyl)benzo[*d*]thiazole (3aa)

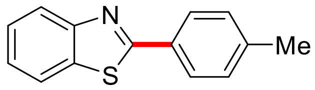

1) **GP1** was followed using benzothiazole (**1a**) (34 mg, 0.25 mmol), 4-iodotoluene (**2a**) (273 mg, 1.25 mmol), Hybrid-Cu (30 mg, 11 mol %) and LiO*t*Bu (60 mg, 0.75 mmol) in Et_2O (0.5 mL). Purification by column chromatography on silica gel (*n*-hexane/EtOAc: 70/1) yielded **3aa** (52.4 mg, 93%) as a light yellow solid.
2) **GP3** was followed using benzothiazole (**1a**) (34 mg, 0.25 mmol), 4-iodotoluene (**2a**) (273 mg, 1.25 mmol), Hybrid-Cu (30 mg, 11 mol %) and Cs_2CO_3 (244 mg, 0.75 mmol) in Et_2O (0.5 mL). Purification by column chromatography on silica gel (*n*-hexane/EtOAc: 4/1) yielded **3aa** (28.7 mg, 51%) as a light yellow solid.

M. p.: 86 ºC.

^{1}H NMR (300 MHz, $CDCl_3$): δ = 7.96 (dd, J = 8.0, 1.3 Hz, 1H), 7.92 – 7.89 (m, 1H), 7.87 (m, 1H), 7.78 (dd, J = 8.0, 1.3 Hz, 1H), 7.38 (ddd, J = 8.0, 7.2, 1.3 Hz, 1H), 7.26 (ddd, J = 8.0, 7.2, 1.3 Hz, 1H), 7.22 – 7.19 (m, 1H), 7.19 – 7.16 (m, 1H), 2.32 (s, 3H).

^{13}C NMR (125 MHz, $CDCl_3$): δ = 168.0 (C_q), 154.0 (C_q), 141.2 (C_q), 134.8 (C_q), 130.8 (C_q), 129.6 (CH), 127.4 (CH), 126.1 (CH), 124.9 (CH), 122.9 (CH), 121.4 (CH), 21.5 (CH_3).

IR (ATR): 3024, 2918, 1611, 1445 ,1074, 816, 756, 691, 485, 434 cm^{-1}.

MS (ESI) *m/z* (relative intensity): 226 (100) $[M+H]^+$, 248 (60) $[M+Na]^+$.

HR-MS (ESI): *m/z* calcd for $C_{14}H_{12}NS$ $[M+H]^+$: 226.0688, found: 226.0688.

The spectral data were in accordance with those reported in the literature.[5]

2-Phenylbenzo[*d*]thiazole (3ab)

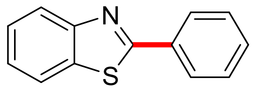

1) **GP1** was followed using benzothiazole (**1a**) (34 mg, 0.25 mmol), iodobenzene (**2b**) (255 mg, 1.25 mmol), Hybrid-Cu (30 mg, 11 mol %) and LiO*t*Bu (60 mg, 0.75 mmol) in Et_2O (0.5 mL). Purification by column chromatography on silica gel (*n*-hexane/EtOAc: 70/1) yielded **3ab** (49.7 mg, 94%) as a light yellow solid.
2) **GP3** was followed using benzothiazole (**1a**) (34 mg, 0.25 mmol), iodobenzene (**2b**) (255 mg, 1.25 mmol), Hybrid-Cu (30 mg, 11 mol %) and Cs_2CO_3 (244 mg, 0.75 mmol) in Et_2O (0.5 mL). Purification by column chromatography on silica gel (*n*-hexane/EtOAc: 4/1) yielded **3ab** (31.2 mg, 59%) as a light yellow solid.

M. p.: 115 ºC.

^{1}H NMR (500 MHz, $CDCl_3$): δ = 8.11 – 8.04 (m, 3H), 7.90 – 7.85 (m, 1H), 7.51 – 7.44 (m, 4H), 7.37 (ddd, *J* = 8.3, 7.2, 1.2 Hz, 1H).

^{13}C NMR (125 MHz, $CDCl_3$): δ = 168.0 (C_q), 154.1 (C_q), 135.0 (C_q), 133.5 (C_q), 130.9 (CH), 128.9 (CH), 127.5 (CH), 126.2 (CH), 125.1 (CH), 123.2 (CH), 121.5 (CH).

IR (ATR): 3016, 1510, 1478, 1433, 1313, 1252, 1215, 963, 918, 750 cm^{-1}.

MS (ESI) *m/z* (relative intensity): 212 (100) $[M+H]^+$.

HR-MS (ESI): *m/z* calcd for $C_{13}H_{10}NS$ $[M+H]^+$: 212.0530, found: 212.0528.

The spectral data were in accordance with those reported in the literature.[5]

2-(4-Methoxyphenyl)benzo[*d*]thiazole (3ac)

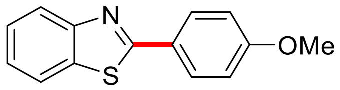

1) **GP1** was followed using benzothiazole (**1a**) (34 mg, 0.25 mmol), 4-iodoanisole (**2c**) (293 mg, 1.25 mmol), Hybrid-Cu (30 mg, 11 mol %) and LiO*t*Bu (60 mg, 0.75 mmol) in Et_2O (0.5 mL). Purification by column chromatography on silica gel (*n*-hexane/EtOAc: 70/1) yielded **3ac** (47.7 mg, 79%) as a light yellow solid.
2) **GP3** was followed using benzothiazole (**1a**) (34 mg, 0.25 mmol), 4-iodoanisole (**2c**) (293 mg, 1.25 mmol), Hybrid-Cu (30 mg, 11 mol %) and Cs_2CO_3 (244 mg, 0.75 mmol) in Et_2O (0.5 mL). Purification by column chromatography on silica gel (*n*-hexane/EtOAc: 4/1) yielded **3ac** (25.9 mg, 43%) as a light yellow solid.

M. p.: 134 ºC.

^{1}H NMR (400 MHz, $CDCl_3$): δ = 8.04 – 7.98 (m, 3H), 7.86 (ddd, *J* = 8.0, 1.3, 0.7 Hz, 1H), 7.45 (ddd, *J* = 8.3, 7.2, 1.3 Hz, 1H), 7.33 (ddd, *J* = 8.0, 7.2, 1.3 Hz, 1H), 7.01 – 6.94 (m, 2H), 3.86 (s, 3H).

^{13}C NMR (100 MHz, $CDCl_3$): δ = 167.8 (C_q), 161.9 (C_q), 154.2 (C_q), 134.8 (C_q), 129.1 (CH), 126.4 (C_q), 126.2 (CH), 124.8 (CH), 122.8 (CH), 121.5 (CH), 114.4 (CH), 55.5 (CH_3).

IR (ATR): 3061, 2994, 2836, 1604, 1483, 1434, 1256, 1225, 1171, 1027 cm^{-1}.

MS (ESI) *m/z* (relative intensity): 242 (100) $[M+H]^+$, 264 (30) $[M+Na]^+$.

HR-MS (ESI): *m/z* calcd for $C_{14}H_{12}NOS$ $[M+H]^+$: 242.0635, found: 242.0634.

The spectral data were in accordance with those reported in the literature.[5]

2-(4-(Trifluoromethyl)phenyl)benzo[*d*]thiazole (3ad)

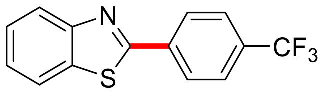

GP1 was followed using benzothiazole (**1a**) (34 mg, 0.25 mmol), 1-iodo-4-(trifluoromethyl)benzene (**2d**) (340 mg, 1.25 mmol), Hybrid-Cu (30 mg, 11 mol %) and LiO*t*Bu (60 mg, 0.75 mmol) in Et_2O (0.5 mL). Purification by column chromatography on silica gel (*n*-hexane/EtOAc: 70/1) yielded **3ad** (64.9 mg, 93%) as a light yellow solid.

M. p.: 161 ºC.

^{1}H NMR (400 MHz, $CDCl_3$): δ = 8.22 – 8.14 (m, 2H), 8.09 (ddd, J = 8.0, 1.2, 0.7 Hz, 1H), 7.91 (ddd, J = 8.0, 1.2, 0.7 Hz, 1H), 7.76 – 7.69 (m, 2H), 7.51 (ddd, J = 8.2, 7.2, 1.2 Hz, 1H), 7.41 (ddd, J = 8.2, 7.2, 1.2 Hz, 1H).

^{13}C NMR (100 MHz, $CDCl_3$): δ = 166.0 (C_q), 154.0 (C_q), 136.8 (d, $J_{C\text{-}F}$ = 2 Hz, C_q), 135.2 (C_q), 132.4 (q, $J_{C\text{-}F}$ = 33 Hz, C_q), 127.8 (CH), 126.6 (CH), 126.0 (q, $J_{C\text{-}F}$= 4 Hz, CH), 125.8 (CH), 123.8 (d, $J_{C\text{-}F}$ = 272 Hz, C_q) 123.6 (CH), 121.7 (CH).

^{19}F NMR (282 MHz, $CDCl_3$): δ = -62.86 (s).

IR (ATR): 3072, 1324, 1214, 1132, 1067, 970, 847, 744, 668, 624 cm^{-1}.

MS (ESI) *m/z* (relative intensity): 280 (100) $[M+H]^+$.

HR-MS (ESI): *m/z* calcd for $C_{14}H_9F_3NS$ $[M+H]^+$: 280.0403, found: 280.0402.

The spectral data were in accordance with those reported in the literature.[5]

2-(4-Fluorophenyl)benzo[*d*]thiazole (3ae)

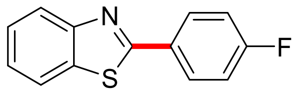

GP1 was followed using benzothiazole (**1a**) (34 mg, 0.25 mmol), 1-fluoro-4-iodobenzene (**2e**) (278 mg, 1.25 mmol), Hybrid-Cu (30 mg, 11 mol %) and LiO*t*Bu (60 mg, 0.75 mmol) in Et_2O (0.5 mL). Purification by column chromatography on silica gel (*n*-hexane/EtOAc: 70/1) yielded **3ae** (54.5 mg, 95%) as a light yellow solid.

M. p.: 101 ºC.

^{1}H NMR (500 MHz, $CDCl_3$): δ = 8.10 – 8.01 (m, 3H), 7.88 (ddd, J = 7.9, 1.2, 0.6 Hz, 1H), 7.48 (ddd, J = 8.3, 7.2, 1.2 Hz, 1H), 7.37 (ddd, J = 8.3, 7.2, 1.2 Hz, 1H), 7.20 – 7.11 (m, 2H).

^{13}C NMR (125 MHz, $CDCl_3$): δ = 166.7 (C_q), 164.4 (d, $J_{C\text{-}F}$= 252 Hz, C_q), 154.1 (C_q), 135.0 (C_q), 129.9 (d, $J_{C\text{-}F}$ = 3 Hz, C_q), 129.49 (d, $J_{C\text{-}F}$ = 9 Hz, CH), 126.39 (CH), 125.22 (CH), 123.16 (CH), 121.59 (CH), 116.13 (d, $J_{C\text{-}F}$ = 22 Hz, CH).

^{19}F NMR (282 MHz, $CDCl_3$) δ –108.94 (tt, J = 8.5, 5.3 Hz).

IR (ATR): 3063, 1602, 1519, 1481, 1434, 1314, 1250, 1097, 967, 831 cm^{-1}.

MS (ESI) *m/z* (relative intensity): 230 (100) $[M+H]^+$.

HR-MS (ESI): *m/z* calcd for $C_{13}H_9FNS$ $[M+H]^+$: 230.0436, found: 230.0434.

The spectral data were in accordance with those reported in the literature.[7]

2-(4-Chlorophenyl)benzo[*d*]thiazole (3af)

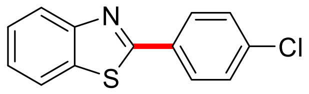

GP1 was followed using benzothiazole (**1a**) (34 mg, 0.25 mmol), 1-chloro-4-iodobenzene (**2f**) (298 mg, 1.25 mmol), Hybrid-Cu (30 mg, 11 mol %) and LiO*t*Bu (60 mg, 0.75 mmol) in Et_2O (0.5 mL). Purification by column chromatography on silica gel (*n*-hexane/EtOAc: 70/1) yielded **3af** (56.5 mg, 92%) as a light yellow solid.

M. p.: 112 ºC.

^{1}H NMR (500 MHz, $CDCl_3$): δ = 8.05 (dt, *J* = 8.2, 0.8 Hz, 1H), 8.02 – 7.98 (m, 2H), 7.88 (ddd, *J* = 8.2, 0.8, 0.8 Hz, 1H), 7.48 (ddd, *J* = 8.2, 7.2, 1.2 Hz, 1H), 7.46 – 7.43 (m, 2H), 7.38 (ddd, *J* = 8.2, 7.2, 1.2 Hz, 1H).

^{13}C NMR (125 MHz, $CDCl_3$): δ = 166.6 (C_q), 154.0 (C_q), 137.0 (C_q), 135.0 (C_q), 132.1 (C_q), 129.2 (CH), 128.7 (CH), 126.5 (CH), 125.4 (CH), 123.3 (CH), 121.6 (CH).

IR (ATR): 3062, 1590, 1474, 1433, 1399, 1314, 1287, 1225, 1091, 1015, 389 cm^{-1}.

MS (ESI) *m/z* (relative intensity): 246 (100) $[M+H]^+$.

HR-MS (ESI): *m/z* calcd for $C_{13}H_9ClNS$ $[M+H]^+$: 246.0139, found: 246.0139.

The spectral data were in accordance with those reported in the literature.[6]

2-(4-Bromophenyl)benzo[*d*]thiazole (3ag)

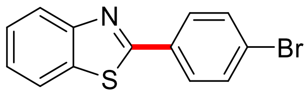

GP1 was followed using benzothiazole (**1a**) (34 mg, 0.25 mmol), 1-bromo-4-iodobenzene (**2g**) (354 mg, 1.25 mmol), Hybrid-Cu (30 mg, 11 mol %) and LiO*t*Bu (60 mg, 0.75 mmol) in Et_2O (0.5 mL). Purification by column chromatography on silica gel (*n*-hexane/EtOAc: 70/1) yielded **3ag** (66.0 mg, 91%) as a light yellow solid.

M. p.: 132 ºC.

^{1}H NMR (500 MHz, $CDCl_3$): δ = 8.05 (dt, J = 8.2, 0.9 Hz, 1H), 7.94 – 7.89 (m, 2H), 7.86 (ddd, J = 8.2, 0.9, 0.9 Hz, 1H), 7.61 – 7.57 (m, 2H), 7.48 (ddd, J = 8.2, 7.2, 1.2 Hz, 1H), 7.37 (ddd, J = 8.2, 7.2, 1.2 Hz, 1H).

^{13}C NMR (125 MHz, $CDCl_3$): δ = 166.6 (C_q), 154.0 (C_q), 135.0 (C_q), 132.4(C_q), 132.1 (CH), 128.8 (CH), 126.4 (CH), 125.4 (CH), 125.4 (C_q), 123.2 (CH), 121.6 (CH).

IR (ATR): 3016, 1574, 1433, 1314, 1215, 1102, 1068, 820, 752, 683, 481 cm^{-1}.

MS (ESI) *m/z* (relative intensity): 289 (100) (^{79}Br) $[M+H]^+$, 291 (100) (^{81}Br) $[M+H]^+$.

HR-MS (ESI): *m/z* calcd for $C_{13}H_9{}^{79}BrNS$ $[M+H]^+$: 289.9635, found: 289.9634.

The spectral data were in accordance with those reported in the literature.[7]

2-(*m*-Tolyl)benzo[*d*]thiazole (3ah)

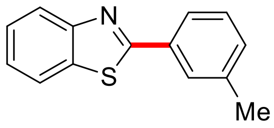

GP1 was followed using benzothiazole (**1a**) (34 mg, 0.25 mmol), 1-iodo-3-methylbenzene (**2h**) (273 mg, 1.25 mmol), Hybrid-Cu (30 mg, 11 mol %) and LiO*t*Bu (60 mg, 0.75 mmol) in Et_2O (0.5 mL). Purification by column chromatography on silica gel (*n*-hexane/EtOAc: 70/1) yielded **3ah** (52.9 mg, 94%) as a light yellow solid.

M. p.: 63 ºC.

^{1}H NMR (400 MHz, $CDCl_3$): δ = 8.06 (dd, *J* = 8.2, 1.2 Hz, 1H), 7.96 – 7.90 (m, 1H), 7.89 (dd, *J* = 8.0, 1.2 Hz, 1H), 7.90 – 7.81 (m, 1H), 7.48 (ddd, *J* = 8.3, 7.2, 1.2 Hz, 1H), 7.37 (ddd, *J* = 7.8, 7.2, 1.2 Hz, 2H), 7.33 – 7.25 (m, 1H), 2.44 (s, 3H).

^{13}C NMR (100 MHz, $CDCl_3$): δ = 168.3 (C_q), 154.1 (C_q), 138.8 (C_q), 135.0 (C_q), 133.5 (C_q), 131.8 (CH), 128.9 (CH), 128.0 (CH), 126.3 (CH), 125.1 (CH), 124.8 (CH), 123.1 (CH), 121.6 (CH), 21.3 (CH_3).

IR (ATR): 3056, 2919, 1606, 1557, 1471, 1378, 1312, 1238, 1171, 1092, 1014 cm^{-1}.

MS (ESI) *m/z* (relative intensity): 226 (100) $[M+H]^+$, 248 (10) $[M+Na]^+$.

HR-MS (ESI): *m/z* calcd for $C_{14}H_{12}NS$ $[M+H]^+$: 226.0683, found: 226.0685.

The spectral data were in accordance with those reported in the literature.[6]

2-(3-Methoxyphenyl)benzo[*d*]thiazole (3ai)

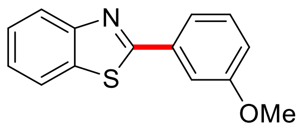

GP1 was followed using benzothiazole (**1a**) (34 mg, 0.25 mmol), 1-iodo-3-methoxybenzene (**2i**) (293 mg, 1.25 mmol), Hybrid-Cu (30 mg, 11 mol %) and LiO*t*Bu (60 mg, 0.75 mmol) in Et_2O (0.5 mL). Purification by column chromatography on silica gel (*n*-hexane/EtOAc: 70/1) yielded **3ai** (45.8 mg, 76%) as a light yellow solid.

M. p.: 82 ºC.

^{1}H NMR (400 MHz, $CDCl_3$): δ = 8.06 (ddd, *J* = 8.3, 1.3, 0.6 Hz, 1H), 7.88 (ddd, *J* = 8.0, 1.3, 0.7 Hz, 1H), 7.66 (dd, *J* = 2.6, 1.6 Hz, 1H), 7.63 (ddd, *J* = 7.6, 1.6, 0.9 Hz, 1H), 7.48 (ddd, *J* = 8.3, 7.2, 1.3 Hz, 1H), 7.41 – 7.34 (m, 2H), 7.03 (ddd, *J* = 8.3, 2.6, 0.9 Hz, 1H), 3.90 (s, 3H).

^{13}C NMR (100 MHz, $CDCl_3$): δ = 167.9 (C_q), 160.0 (C_q), 154.0 (C_q), 135.1 (C_q), 134.9 (C_q), 130.0 (CH), 126.3 (CH), 125.2 (CH), 123.2 (CH), 121.6 (CH), 120.2 (CH), 117.3 (CH), 112.0 (CH), 55.5 (CH_3).

IR (ATR): 3066, 2932, 2834, 2160, 2039, 1582, 1484, 1433, 1287, 1008, 758 cm^{-1}.

MS (ESI) *m/z* (relative intensity): 242 (100) $[M+H]^+$, 264 (35) $[M+Na]^+$.

HR-MS (ESI): *m/z* calcd for $C_{14}H_{12}NOS$ $[M+H]^+$: 242.0637, found: 242.0634.

The spectral data were in accordance with those reported in the literature.[6]

2-[3-(Trifluoromethyl)phenyl]benzo[*d*]thiazole (3aj)

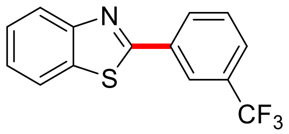

GP1 was followed using benzothiazole (**1a**) (34 mg, 0.25 mmol), 1-iodo-3-(trifluoromethyl)benzene (**2j**) (340 mg, 1.25 mmol), Hybrid-Cu (30 mg, 11 mol %) and LiO*t*Bu (60 mg, 0.75 mmol) in Et_2O (0.5 mL). Purification by column chromatography on silica gel (*n*-hexane/EtOAc: 70/1) yielded **3aj** (53.0 mg, 76%) as a light yellow solid.

M. p.: 87 ºC.

^{1}H NMR (400 MHz, $CDCl_3$): δ = 8.38 – 8.34 (m, 1H), 8.23 (ddd, *J* = 7.2, 1.3, 0.7 Hz, 1H), 8.09 (ddd, *J* = 8.2, 1.3, 0.7 Hz, 1H), 7.91 (ddd, *J* = 8.0, 1.3, 0.7 Hz, 1H), 7.73 (ddd, *J* = 7.8, 1.3, 0.7 Hz, 1H), 7.61 (ddd, *J* = 7.8, 1.3, 0.7 Hz, 1H), 7.51 (ddd, *J* = 8.2, 7.2, 1.3 Hz, 1H), 7.41 (ddd, *J* = 8.0, 7.2, 1.3 Hz, 1H).

^{13}C NMR (100 MHz, $CDCl_3$): δ = 166.1 (C_q), 154.0 (C_q), 135.1 (C_q), 134.4 (C_q), 131.6 (q, $J_{C\text{-}F}$ = 32 Hz, C_q), 130.7 (CH), 129.6 (CH), 127.3 (q, $J_{C\text{-}F}$ = 4 Hz, CH), 126.6 (CH), 125.7 (CH), 124.2 (q, $J_{C\text{-}F}$ = 4 Hz, CH), 123.8 (d, $J_{C\text{-}F}$ = 273 Hz, C_q), 123.5 (CH), 121.7 (CH).

^{19}F NMR (282 MHz, $CDCl_3$): δ = -62.76 (s).

IR (ATR): 3066, 2923, 1613, 1584, 1479, 1442, 1257, 1152, 1076, 937, 880 cm^{-1}.

MS (ESI) *m/z* (relative intensity): 280 (100) $[M+H]^+$.

HR-MS (ESI): *m/z* calcd for $C_{14}H_9F_3NS$ $[M+H]^+$: 280.0403, found: 280.0402.

The spectral data were in accordance with those reported in the literature.[6]

2-(3-Fluorophenyl)benzo[*d*]thiazole (3ak)

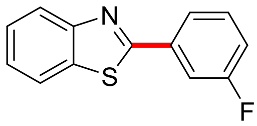

GP1 was followed using benzothiazole (**1a**) (34 mg, 0.25 mmol), 1-fluoro-3-iodobenzene (**2k**) (278 mg, 1.25 mmol), Hybrid-Cu (30 mg, 11 mol %) and LiO*t*Bu (60 mg, 0.75 mmol) in Et_2O (0.5 mL). Purification by column chromatography on silica gel (*n*-hexane/EtOAc: 70/1) yielded **3ak** (52.2 mg, 91%) as a light yellow solid.

M. p.: 72 ºC.

^{1}H NMR (500 MHz, $CDCl_3$): δ = 8.07 (dd, J = 8.2, 0.8 Hz, 1H), 7.89 (dd, J = 8.0, 0.8 Hz, 1H), 7.86 – 7.79 (m, 2H), 7.49 (ddd, J = 8.2, 7.2, 1.2 Hz, 1H), 7.44 (ddd, J = 8.2, 8.0, 5.7 Hz, 1H), 7.39 (ddd, J = 8.2, 7.2, 1.2 Hz, 1H), 7.17 (ddd, J = 8.2, 2.6, 1.2 Hz, 1H).

^{13}C NMR (125 MHz, $CDCl_3$): δ = 166.4 (d, $J_{C\text{-}F}$= 3 Hz, C_q), 163.0 (d, $J_{C\text{-}F}$ = 247 Hz, C_q), 153.9 (C_q), 135.6 (d, $J_{C\text{-}F}$ = 8 Hz, C_q), 135.0 (C_q), 130.6 (d, $J_{C\text{-}F}$ = 8 Hz, CH), 126.5 (CH), 125.5 (CH), 123.4 (CH), 123.3 (d, $J_{C\text{-}F}$ = 3 Hz, CH), 121.6 (CH), 117.8 (d, $J_{C\text{-}F}$ = 21 Hz), 114.26 (d, $J_{C\text{-}F}$ = 24 Hz).

^{19}F NMR (282 MHz, $CDCl_3$): δ = –112.02 (ddd, J = 9.2, 8.5, 5.7 Hz).

IR (ATR): 3053, 2162, 1590, 1457, 1423, 1312, 1230, 1160, 783, 700, 653 cm^{-1}.

MS (ESI) *m/z* (relative intensity): 230 (100) $[M+H]^+$.

HR-MS (ESI): *m/z* calcd for $C_{13}H_9FNS$ $[M+H]^+$: 230.0437, found: 230.0435.

The spectral data were in accordance with those reported in the literature.[6]

2-(3-Chlorophenyl)benzo[*d*]thiazole (3al)

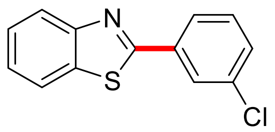

GP1 was followed using benzothiazole (**1a**) (34 mg, 0.25 mmol), 1-chloro-3-iodobenzene (**2l**) (298 mg, 1.25 mmol), Hybrid-Cu (30 mg, 11 mol %) and LiO*t*Bu (60 mg, 0.75 mmol) in Et_2O (0.5 mL). Purification by column chromatography on silica gel (*n*-hexane/EtOAc: 70/1) yielded **3al** (39.9 mg, 65%) as a light yellow solid.

M. p.: 96 ºC.

^{1}H NMR (500 MHz, $CDCl_3$): δ = 8.11 – 8.09 (m, 1H), 8.07 (ddd, J = 8.2, 1.2, 0.7 Hz, 1H), 7.93 (ddd, J = 8.0, 7.2, 1.2 Hz, 1H), 7.90 (ddd, J = 8.0, 1.2, 0.7 Hz, 1H), 7.50 (ddd, J = 8.2, 7.2, 1.2 Hz, 1H), 7.46 – 7.37 (m, 3H).

^{13}C NMR (125 MHz, $CDCl_3$): δ = 166.3 (C_q), 154.0 (C_q), 135.2 (C_q), 135.1 (C_q), 135.1 (C_q), 130.8 (CH), 130.2 (CH), 127.4 (CH), 126.5 (CH), 125.7 (CH), 125.5 (CH), 123.4 (CH), 121.7 (CH).

IR (ATR): 3060, 2924, 1693, 1562, 1457, 1312, 1227, 1070, 975, 862, 782 cm^{-1}.

MS (ESI) *m/z* (relative intensity): 246 (100) $[M+H]^+$.

HR-MS (ESI): *m/z* calcd for $C_{13}H_9ClNS$ $[M+H]^+$: 246.0140, found: 246.0139.

The spectral data were in accordance with those reported in the literature.[6]

2-(3-Bromophenyl)benzo[*d*]thiazole (3am)

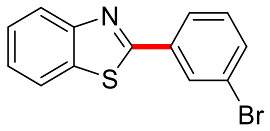

GP1 was followed using benzothiazole (**1a**) (34 mg, 0.25 mmol), 1-bromo-3-iodobenzene (**2m**) (354 mg, 1.25 mmol), Hybrid-Cu (30 mg, 11 mol %) and LiO*t*Bu (60 mg, 0.75 mmol) in Et_2O (0.5 mL). Purification by column chromatography on silica gel (*n*-hexane/EtOAc: 70/1) yielded **3am** (42.8 mg, 59%) as a light yellow solid.

M. p.: 88 ºC.

^{1}H NMR (500 MHz, $CDCl_3$): δ = 8.27 – 8.25 (m, 1H), 8.06 (ddd, J = 8.0, 0.9 Hz, 1H), 7.97 (ddd, J = 7.8, 1.7, 0.9 Hz, 1H), 7.90 (ddd, J = 8.0, 1,2, 0.9 Hz, 1H), 7.60 (ddd, J = 8.0, 2.0, 0.9 Hz, 1H), 7.50 (ddd, J = 8.2, 7.2, 1.2 Hz, 1H), 7.39 (ddd, J = 8.2, 7.2, 1.2 Hz, 1H), 7.34 (dd, J = 7.9 Hz, 1H).

^{13}C NMR (125 MHz, $CDCl_3$): δ = 166.1 (C_q), 153.9 (C_q), 135.4 (C_q), 135.1 (C_q), 133.8 (CH), 130.5 (CH), 130.2 (CH), 126.5 (CH), 126.1 (CH), 125.6 (CH), 123.4 (CH), 123.2 (C_q), 121.7 (CH).

IR (ATR): 3059, 2923, 1601, 1486, 1455, 1224, 1119, 957, 726, 630, 449 cm^{-1}.

MS (ESI) *m/z* (relative intensity): 289 (100) (^{79}Br) $[M+H]^+$, 291 (100) (^{81}Br) $[M+H]^+$.

HR-MS (ESI): *m/z* calcd for $C_{13}H_9{}^{79}BrNS$ $[M+H]^+$: 289.9634, found: 289.9634.

The spectral data were in accordance with those reported in the literature.[8]

2-(*o*-Tolyl)benzo[*d*]thiazole (3an)

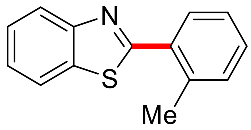

GP1 was followed using benzothiazole (**1a**) (34 mg, 0.25 mmol), 1-iodo-2-methylbenzene (**2n**) (273 mg, 1.25 mmol), Hybrid-Cu (30 mg, 11 mol %) and LiO*t*Bu (60 mg, 0.75 mmol) in Et_2O (0.5 mL). Purification by column chromatography on silica gel (*n*-hexane/EtOAc: 70/1) yielded **3an** (20.3 mg, 36%) as a yellow oil.

^{1}H NMR (400 MHz, $CDCl_3$): δ = 8.09 (ddd, J = 8.2, 1.2, 0.6 Hz, 1H), 7.92 (ddd, J = 8.2, 1.2, 0.6 Hz, 1H), 7.74 (dd, J = 7.4, 1.4 Hz, 1H), 7.49 (ddd, J = 8.2, 7.4, 1.4 Hz, 1H), 7.42 – 7.37 (m, 1H), 7.37 – 7.27 (m, 3H), 2.64 (s, 3H).

^{13}C NMR (100 MHz, $CDCl_3$): δ = 168.0 (C_q), 153.7 (C_q), 137.2 (C_q), 135.6 (C_q), 133.1 (C_q), 131.5 (CH), 130.5 (CH), 130.0 (CH), 126.1 (CH), 126.1 (CH), 125.1 (CH), 123.4 (CH), 121.3 (CH), 21.3 (CH_3).

IR (ATR): 2924, 2838, 2612, 1977, 1571, 1500, 1115, 1021, 970, 754, 551 cm^{-1}.

MS (ESI) *m/z* (relative intensity): 226 (100) $[M+H]^+$, 248 (5) $[M+Na]^+$.

HR-MS (ESI): *m/z* calcd for $C_{14}H_{12}NO$ $[M+H]^+$: 226.0686, found: 226.0685.

The spectral data were in accordance with those reported in the literature.[5]

2-(2-Methoxyphenyl)benzo[*d*]thiazole (3ao)

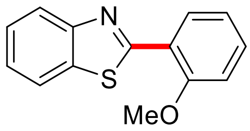

GP1 was followed using benzothiazole (**1a**) (34 mg, 0.25 mmol), 1-iodo-2-methoxybenzene (**2o**) (293 mg, 1.25 mmol), Hybrid-Cu (30 mg, 11 mol %) and LiO*t*Bu (60 mg, 0.75 mmol) in Et_2O (0.5 mL). Purification by column chromatography on silica gel (*n*-hexane/EtOAc: 70/1) yielded **3ao** (21.7 mg, 36%) as a light yellow solid.

M. p.: 108 ºC.

^{1}H NMR (400 MHz, $CDCl_3$): δ = 8.51 (ddd, *J* = 7.9, 1.8, 0.6 Hz, 1H), 8.07 (ddd, *J* = 8.2, 1.1, 0.6 Hz, 1H), 7.91 (ddd, *J* = 7.9, 1.1, 0.6 Hz, 1H), 7.50 – 7.41 (m, 2H), 7.38 – 7.32 (m, 1H), 7.12 (ddd, *J* = 7.9, 7.3, 1.1 Hz, 1H), 7.06 (dd, *J* = 8.2, 1.1 Hz, 1H), 4.05 (s, 3H).

^{13}C NMR (100 MHz, $CDCl_3$): δ = 163.1 (C_q), 157.2 (C_q), 152.2 (C_q), 136.1 (C_q), 131.7 (CH), 129.6 (CH), 125.9 (CH), 124.6 (CH), 124.1 (CH), 122.8 (CH), 122.3 (C_q), 121.2 (CH), 111.7 (CH), 55.7 (CH_3).

IR (ATR): 3007, 2971, 1893, 1519, 1181,1122, 1054, 964, 825, 587, 416 cm^{-1}.

MS (ESI) *m/z* (relative intensity): 242 (100) $[M+H]^+$, 264 (30) $[M+Na]^+$.

HR-MS (ESI): *m/z* calcd for $C_{14}H_{12}NOS$ $[M+H]^+$: 242.0637, found: 242.0635.

The spectral data were in accordance with those reported in the literature.[23]

6-Methoxy-2-(*p*-tolyl)benzo[*d*]thiazole (3ba)

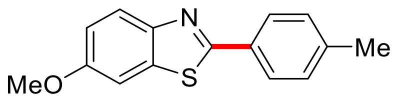

GP1 was followed using 6-methoxybenzo[d]thiazole (**1b**) (41 mg, 0.25 mmol), 4-iodotoluene (**2a**) (273 mg, 1.25 mmol), Hybrid-Cu (30 mg, 11 mol %) and LiO*t*Bu (60 mg, 0.75 mmol) in Et_2O (0.5 mL). Purification by column chromatography on silica gel (*n*-hexane/EtOAc: 70/1) yielded **3aa** (35.1 mg, 55%) as a light yellow solid.

M. p.: 115 ºC.

^{1}H NMR (400 MHz, $CDCl_3$): δ = 7.93 – 7.88 (m, 3H), 7.33 – 7.30 (m, 1H), 7.28 – 7.23 (m, 2H), 7.06 (dd, J = 8.9, 2.6 Hz, 1H), 3.86 (s, 3H), 2.39 (s, 3H).

^{13}C NMR (100 MHz, $CDCl_3$): δ = 165.7 (C_q), 157.6 (C_q), 148.7 (C_q), 140.9 (C_q), 136.2 (C_q), 131.1 (C_q), 129.6 (CH), 127.1 (CH), 123.5 (CH), 115.4 (CH), 104.2 (CH), 55.7 (CH_3), 21.4 (CH_3).

IR (ATR): 3020, 1606, 1488, 1411, 1321, 1119, 968, 903, 747, 667, 479 cm^{-1}.

MS (ESI) *m/z* (relative intensity): 256 (100) $[M+H]^+$.

HR-MS (ESI): *m/z* calcd for $C_{15}H_{14}NOS$ $[M+H]^+$: 256.0795, found: 256.0793.

The spectral data were in accordance with those reported in the literature.[13]

6-Fluoro-2-(*p*-tolyl)benzo[*d*]thiazole (3ca)

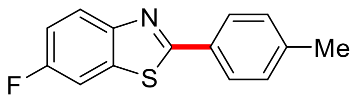

GP1 was followed using 6-fluorobenzo[d]thiazole (**1c**) (38 mg, 0.25 mmol), 4-iodotoluene (**2a**) (273 mg, 1.25 mmol), Hybrid-Cu (30 mg, 11 mol %) and LiO*t*Bu (60 mg, 0.75 mmol) in Et_2O (0.5 mL). Purification by column chromatography on silica gel (*n*-hexane/EtOAc: 70/1) yielded **3aa** (57.8 mg, 95%) as a light yellow solid.

M. p.: 149 ºC.

^{1}H NMR (400 MHz, $CDCl_3$): δ = 7.96 (dd, J = 8.9, 4.9 Hz, 2H), 7.96 – 7.87 (m, 3H), 7.53 (ddd, J = 8.1, 2.7, 0.5 Hz, 1H), 7.31 – 7.23 (m, 2H), 7.19 (dd, J = 8.9, 2.7 Hz, 1H), 2.43 – 2.37 (m, 3H).

^{13}C NMR (100 MHz, $CDCl_3$): δ = 167.9 (C_q), 160.3 (d, $J_{C\text{-}F}$ = 245 Hz, C_q), 150.8 (C_q), 141.45 (C_q), 135.87 (d, $J_{C\text{-}F}$ = 11.2 Hz, C_q), 130.66 (C_q), 129.71 (CH), 127.31 (CH), 123.86 (d, $J_{C\text{-}F}$ = 9.3 Hz, CH), 114.76 (d, $J_{C\text{-}F}$ = 24.6 Hz, CH), 107.74 (d, $J_{C\text{-}F}$ = 26.7 Hz, CH), 21.47 (CH_3).

^{19}F NMR (282 MHz, $CDCl_3$): δ = –116.20 (td, J = 8.5, 4.8 Hz).

IR (ATR): 2916, 2161, 1483, 1441, 1307, 1050, 867, 816, 761, 694, 491 cm^{-1}.

MS (ESI) *m/z* (relative intensity): 244 (100) $[M+H]^+$.

HR-MS (ESI): *m/z* calcd for $C_{14}H_{11}FNS$ $[M+H]^+$: 244.0593, found: 244.0591.

The spectral data were in accordance with those reported in the literature.[13]

6-Chloro-2-(*p*-tolyl)benzo[*d*]thiazole (3da)

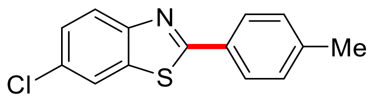

GP1 was followed using 6-chlorobenzo[d]thiazole (**1d**) (42 mg, 0.25 mmol), 4-iodotoluene (**2a**) (273 mg, 1.25 mmol), Hybrid-Cu (30 mg, 11 mol %) and Li*Ot*Bu (60 mg, 0.75 mmol) in Et_2O (0.5 mL). Purification by column chromatography on silica gel (*n*-hexane/EtOAc: 70/1) yielded **3aa** (55.8 mg, 86%) as a light yellow solid.

M. p.: 160 ºC.

^{1}H NMR (400 MHz, $CDCl_3$): δ = 7.95 – 7.89 (m, 3H), 7.83 (dd, *J* = 2.1, 0.5 Hz, 1H), 7.41 (dd, *J* = 8.7, 2.1 Hz, 1H), 7.30 – 7.25 (m, 2H), 2.41 (s, 3H).

^{13}C NMR (100 MHz, $CDCl_3$): δ = 168.7 (C_q), 152.7 (C_q), 141.7 (C_q), 136.1 (C_q), 130.8 (C_q), 130.5 (C_q), 129.8 (CH), 127.4 (CH), 127.0 (CH), 123.7 (CH), 121.1 (CH), 21.5 (CH_3).

IR (ATR): 2916, 1934, 1585, 1483, 1437, 1304, 1181, 1087, 963, 816, 561 cm^{-1}.

MS (ESI) *m/z* (relative intensity): 260 (100) $[M+H]^+$.

HR-MS (ESI): *m/z* calcd for $C_{14}H_{11}ClNS$ $[M+H]^+$: 260.0296, found: 260.0295.

The spectral data were in accordance with those reported in the literature.[14]

6-Bromo-2-(*p*-tolyl)benzo[*d*]thiazole (3ea)

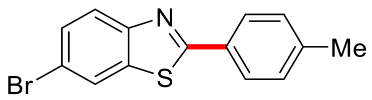

GP1 was followed using 6-bromobenzo[d]thiazole(**1e**) (53 mg, 0.25 mmol), 4-iodotoluene (**2a**) (273 mg, 1.25 mmol), Hybrid-Cu (30 mg, 11 mol %) and LiO*t*Bu (60 mg, 0.75 mmol) in Et_2O (0.5 mL). Purification by column chromatography on silica gel (*n*-hexane/EtOAc: 70/1) yielded **3aa** (50.2 mg, 66%) as a light yellow solid.

M. p.: 152 ºC.

^{1}H NMR (400 MHz, $CDCl_3$): δ = 8.00 (dd, J = 2.0, 0.5 Hz, 1H), 7.96 – 7.92 (m, 2H), 7.87 (dd, J = 8.7, 0.5 Hz, 1H), 7.58 – 7.53 (m, 1H), 7.31 – 7.25 (m, 2H), 2.41 (s, 3H).

^{13}C NMR (100 MHz, $CDCl_3$): δ = 168.7 (C_q), 153.0 (C_q), 141.8 (C_q), 136.6 (C_q), 130.5 (C_q), 129.8 (CH), 129.7 (CH), 127.5 (CH), 124.1 (CH), 124.1 (CH), 118.5 (C_q), 21.5 (CH_3).

IR (ATR): 3061, 1621, 1508, 1459, 1313, 1219, 1126, 1020, 694, 877, 802 cm^{-1}.

MS (ESI) *m/z* (relative intensity): 303 (100) (^{79}Br) [M+H]$^+$, 305 (100) (^{81}Br) [M+H]$^+$.

HR-MS (ESI): *m/z* calcd for $C_{14}H_{11}{}^{79}BrNS$ [M+H]$^+$: 303.9792, found: 303.9790.

The spectral data were in accordance with those reported in the literature.[15]

2-[3,5-Bis(trifluoromethyl)phenyl]benzo[*d*]thiazole (3ap)

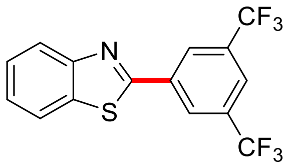

GP1 was followed using benzothiazole (**1a**) (mg, 0.25 mmol), 1-iodo-3,5-bis(trifluoromethyl)benzene (**2p**) (425 mg, 1.25 mmol), Hybrid-Cu (30 mg, 11 mol %) and LiO*t*Bu (60 mg, 0.75 mmol) in Et_2O (0.5 mL). Purification by column chromatography on silica gel (*n*-hexane/EtOAc: 70/1) yielded **3ap** (50.4 mg, 58%) as a light yellow solid.

M. p.: 134 ºC.

^{1}H NMR (500 MHz, $CDCl_3$): δ = 8.53 – 8.49 (m, 2H), 8.11 (dd, J = 8.2, 0.9 Hz, 1H), 7.97 – 7.95 (m, 1H), 7.95 – 7.91 (m, 1H), 7.54 (ddd, J = 8.2, 7.2, 1.2 Hz, 1H), 7.45 (ddd, J = 8.2, 7.2, 1.2 Hz, 1H).

^{13}C NMR (125 MHz, $CDCl_3$): δ = 164.1 (C_q), 153.8 (C_q), 135.6 (C_q), 135.1 (C_q), 132.6 (q, $J_{C\text{-}F}$ = 34 Hz, C_q), 127.3 (q, $J_{C\text{-}F}$ = 4 Hz, CH), 127.0 (CH), 126.2 (CH), 124.0 (q, $J_{C\text{-}F}$ = 4 Hz, CH), 123.9 (CH), 121.9 (CH), 120.8 (d, $J_{C\text{-}F}$ = 273 Hz, C_q).

^{19}F NMR (282 MHz, $CDCl_3$): δ = -62.95 (s).

IR (ATR): 3060, 2924, 1692, 1587, 1433, 1375, 1311, 1258, 1218, 1134, 1029 cm^{-1}.

MS (ESI) *m/z* (relative intensity): 348 (100) $[M+H]^+$.

HR-MS (ESI): *m/z* calcd for $C_{15}H_8F_6NS$ $[M+H]^+$: 348.0278, found: 348.0276.

The spectral data were in accordance with those reported in the literature.[16]

2-(3,4-Dichlorophenyl)benzo[*d*]thiazole (3aq)

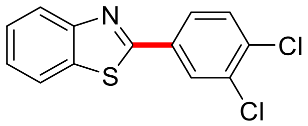

GP1 was followed using benzothiazole (**1a**) (mg, 0.25 mmol), 1,2-dichloro-4-iodobenzene (**2q**) (341 mg, 1.25 mmol), Hybrid-Cu (30 mg, 11 mol %) and LiO*t*Bu (60 mg, 0.75 mmol) in Et_2O (0.5 mL). Purification by column chromatography on silica gel (*n*-hexane/EtOAc: 70/1) yielded **3aq** (37.1 mg, 53%) as a light yellow solid.

M. p.: 108 ºC.

^{1}H NMR (500 MHz, $CDCl_3$): δ = 8.19 (d, J = 2.1 Hz, 1H), 8.05 (dd, J = 8.3, 0.9 Hz, 1H), 7.91 – 7.85 (m, 2H), 7.54 (d, J = 8.3 Hz, 1H), 7.50 (ddd, J = 8.3, 7.2, 1.2 Hz, 1H), 7.40 (ddd, J = 8.3, 7.2, 1.2 Hz, 1H).

^{13}C NMR (125 MHz, $CDCl_3$): δ = 165.1 (C_q), 153.9 (C_q), 135.1 (C_q), 135.0 (C_q), 133.5 (C_q), 133.4 (C_q), 131.0 (CH), 129.0 (CH), 126.7 (CH), 126.5 (CH), 125.7 (CH), 123.5 (CH), 121.7 (CH).

IR (ATR): 2919, 1621, 1555, 1500, 1451, 1215, 1109, 1054, 821, 742, 640 cm^{-1}.

MS (ESI) *m/z* (relative intensity): 279 (100) $[M+H]^+$.

HR-MS (ESI): *m/z* calcd for $C_{13}H_8Cl_2NS$ $[M+H]^+$: 279.9748, found: 279.9749.

The spectral data were in accordance with those reported in the literature.[17]

2-(*p*-Tolyl)thiazole (3fa)

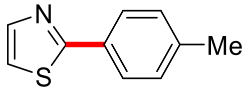

GP1 was followed using thiazole (**1f**) (21 mg, 0.25 mmol), 4-iodotoluene (**2a**) (273 mg, 1.25 mmol), Hybrid-Cu (30 mg, 11 mol %) and LiO*t*Bu (60 mg, 0.75 mmol) in Et_2O (0.5 mL). Purification by column chromatography on silica gel (*n*-hexane/EtOAc: 70/1) yielded **3fa** (37.7 mg, 86%) as a yellow oil.

^{1}H NMR (500 MHz, $CDCl_3$): δ = 7.87 – 7.83 (m, 2H), 7.82 (d, J = 3.3 Hz, 1H), 7.26 (d, J = 3.3 Hz, 1H), 7.24 – 7.20 (m, 2H), 2.37 (s, 3H).

^{13}C NMR (125 MHz, $CDCl_3$): δ = 168.6 (C_q), 143.5 (CH), 140.2 (C_q), 130.9 (C_q), 129.6 (CH), 126.4 (CH), 118.3 (CH), 21.4 (CH_3).

IR (ATR): 3067, 1613, 1473, 1328, 1162, 1068, 970, 807, 648, 546 cm^{-1}.

MS (ESI) *m/z* (relative intensity): 176 (100) $[M+H]^+$.

HR-MS (ESI): *m/z* calcd for $C_{10}H_{10}NS$ $[M+H]^+$: 176.0529, found: 176.0528.

The spectral data were in accordance with those reported in the literature.[20]

5-Phenyl-2-(*m*-tolyl)thiazole (3gh)

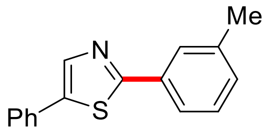

GP1 was followed using 5-phenylthiazole (**1g**) (40 mg, 0.25 mmol), 3-iodotoluene (**2h**) (273 mg, 1.25 mmol), Hybrid-Cu (30 mg, 11 mol %) and LiO*t*Bu (60 mg, 0.75 mmol) in Et_2O (0.5 mL). Purification by column chromatography on silica gel (*n*-hexane/EtOAc: 70/1) yielded **3gh** (52.2 mg, 83%) as a light yellow solid.

M. p.: 101 ºC.

^{1}H NMR (300 MHz, $CDCl_3$): δ = 8.00 (s, 1H), 7.82 – 7.79 (m, 1H), 7.74 (ddd, J = 7.7, 1.8, 1.2 Hz, 1H), 7.62 – 7.59 (m, 1H), 7.59 – 7.57 (m, 1H), 7.45 – 7.28 (m, 5H), 7.24 – 7.21 (m, 1H), 2.42 (s, 3H).

^{13}C NMR (125 MHz, $CDCl_3$): δ = 167.3 (C_q), 139.0 (C_q), 139.0 (CH), 138.7 (C_q), 133.5 (C_q), 131.4 (C_q), 130.8 (CH), 129.0 (CH), 128.8 (CH), 128.2 (CH), 126.8 (CH), 126.6 (CH), 123.5 (CH), 21.4 (CH_3).

IR (ATR): 2918, 2855, 1547, 1515, 1453, 1237, 1109, 1001, 956, 813, 792 cm^{-1}.

MS (ESI) *m/z* (relative intensity): 252 (100) $[M+H]^+$, 274 (3) $[M+Na]^+$.

HR-MS (ESI): *m/z* calcd for $C_{16}H_{14}NS$ $[M+H]^+$: 252.0843, found: 252.0841.

The spectral data were in accordance with those reported in the literature.[6]

4,5-Dimethyl-2-(*p*-tolyl)thiazole (3ha)

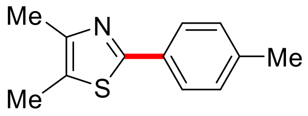

GP1 was followed using 4,5-dimethylthiazole (**1h**) (28 mg, 0.25 mmol), 4-iodotoluene (**2a**) (273 mg, 1.25 mmol), Hybrid-Cu (30 mg, 11 mol %) and LiO*t*Bu (60 mg, 0.75 mmol) in Et_2O (0.5 mL). Purification by column chromatography on silica gel (*n*-hexane/EtOAc: 70/1) yielded **3ha** (43.7 mg, 86%) as a light yellow solid.

M. p.: 62 ºC.

^{1}H NMR (500 MHz, $CDCl_3$): δ = 7.77 – 7.68 (m, 2H), 7.18 (d, J = 8.0 Hz, 2H), 2.36 (s, 6H), 2.35 (s, 3H).

^{13}C NMR (125 MHz, $CDCl_3$): δ = 163.5 (C_q), 149.0 (C_q), 139.4 (C_q), 131.3 (C_q), 129.4 (CH), 126.0 (CH), 125.9 (C_q), 21.3 (CH_3), 14.8 (CH_3), 11.4 (CH_3).

IR (ATR): 2955, 2923, 2854, 1810, 1480, 1448, 1143, 1082, 847, 785, 689 cm^{-1}.

MS (ESI) *m/z* (relative intensity): 204 (100) $[M+H]^+$.

HR-MS (ESI): *m/z* calcd for $C_{12}H_{14}NS$ $[M+H]^+$: 204.0842, found: 204.0841.

The spectral data were in accordance with those reported in the literature.[21]

2-(4-Chlorophenyl)-4,5-dimethylthiazole (3hf)

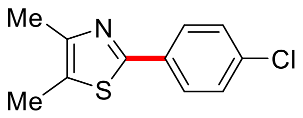

GP1 was followed using 4,5-dimethylthiazole (**1h**) (28 mg, 0.25 mmol1-chloro-4-iodobenzene (**2f**) (298 mg, 1.25 mmol), Hybrid-Cu (30 mg, 11 mol %) and LiO*t*Bu (60 mg, 0.75 mmol) in Et_2O (0.5 mL). Purification by column chromatography on silica gel (*n*-hexane/EtOAc: 70/1) yielded **3hf** (48.7 mg, 87%) as a light yellow solid.

M. p.: 93 ºC.

^{1}H NMR (400 MHz, $CDCl_3$): δ = 7.78 (ddd, J = 9.1, 2.9, 2.1 Hz, 2H), 7.35 (ddd, J = 9.1, 2.9, 2.1 Hz, 2H), 2.36 (s, 6H).

^{13}C NMR (100 MHz, $CDCl_3$): δ = 161.9 (C_q), 149.5 (C_q), 135.2 (C_q), 132.5 (C_q), 129.0 (CH), 127.2 (CH), 127.0 (C_q), 14.8 (CH_3), 11.5 (CH_3).

IR (ATR): 2922, 1724, 1679, 1611, 1494, 1364, 1257, 1076, 918, 731 cm^{-1}.

MS (ESI) *m/z* (relative intensity): 224 (100) $[M+H]^+$.

HR-MS (ESI): *m/z* calcd for $C_{11}H_{11}ClNS$ $[M+H]^+$: 224.0296, found: 224.0295.

The spectral data were in accordance with those reported in the literature.[6]

2-(*p*-Tolyl)benzo[*d*]oxazole (5aa)

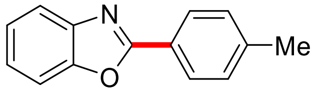

GP1 was followed using benzoxazole (**4a**) (30 mg, 0.25 mmol), 4-iodotoluene (**2a**) (273 mg, 1.25 mmol), Hybrid-Cu (30 mg, 11 mol %) and LiO*t*Bu (60 mg, 0.75 mmol) in Et_2O (0.5 mL). Purification by column chromatography on silica gel (*n*-hexane/EtOAc: 70/1) yielded **5aa** (41.3 mg, 79%) as a light yellow solid.

M. p.: 123 ºC.

^{1}H NMR (500 MHz, $CDCl_3$): δ = 8.13 (d, J = 8.3 Hz, 2H), 7.79 – 7.69 (m, 1H), 7.60 – 7.50 (m, 1H), 7.32 – 7.29 (m, 3H), 2.41 (s, 3H).

^{13}C NMR (125 MHz, $CDCl_3$): δ = 163.2 (C_q), 150.6 (C_q), 142.1 (C_q), 142.0 (C_q), 129.6 (CH), 127.5 (CH), 124.8 (CH), 124.4 (CH), 124.3 (C_q), 119.8 (CH), 110.4 (CH), 21.6 (CH_3).

IR (ATR): 3024, 2919, 1608, 1579, 1556, 1410, 1200, 1055, 926, 756, 380 cm^{-1}.

MS (ESI) *m/z* (relative intensity): 210 (100) $[M+H]^+$.

HR-MS (ESI): *m/z* calcd for $C_{14}H_{12}NO$ $[M+H]^+$: 210.0915, found: 210.0913.

The spectral data were in accordance with those reported in the literature.[15]

5-Methyl-2-(*p*-tolyl)benzo[*d*]oxazole (5ba)

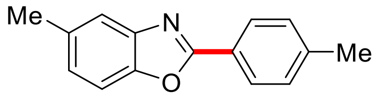

GP1 was followed using 5-methylbenzo[d]oxazole (**4b**) (33 mg, 0.25 mmol), 4-iodotoluene (**2a**) (273 mg, 1.25 mmol), Hybrid-Cu (30 mg, 11 mol %) and LiO*t*Bu (60 mg, 0.75 mmol) in Et_2O (0.5 mL). Purification by column chromatography on silica gel (*n*-hexane/EtOAc: 70/1) yielded **5ba** (40.7 mg, 73%) as a light yellow solid.

M. p.: 135 ºC.

^{1}H NMR (400 MHz, $CDCl_3$): δ = 8.14 – 8.07 (m, 2H), 7.54 – 7.50 (m, 1H), 7.41 (d, J = 8.2 Hz, 1H), 7.33 – 7.26 (m, 2H), 7.11 (ddd, J = 8.2, 1.7, 0.7 Hz, 1H), 2.47 – 2.45 (m, 3H), 2.41 (s, 3H).

^{13}C NMR (100 MHz, $CDCl_3$): δ = 163.3 (C_q), 148.9 (C_q), 142.3 (C_q), 141.8 (C_q), 134.2 (C_q), 129.6 (CH), 127.5 (CH), 125.9 (CH), 124.5 (C_q), 119.7 (CH), 109.8 (CH), 21.6 (CH_3), 21.5 (CH_3).

IR (ATR): 2958, 2866, 1616, 1581, 1268, 1018, 823, 807, 729, 650, 500 cm^{-1}.

MS (ESI) *m/z* (relative intensity): 224 (100) $[M+H]^+$, 246 (5) $[M+Na]^+$.

HR-MS (ESI): *m/z* calcd for $C_{15}H_{14}NO$ $[M+H]^+$: 224.1068, found: 224.1070.

The spectral data were in accordance with those reported in the literature.[18]

5-(*tert*-Butyl)-2-(*p*-tolyl)benzo[*d*]oxazole (5ca)

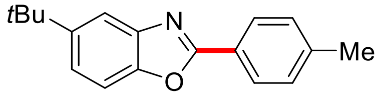

GP1 was followed using 5-(tert-butyl)benzo[d]oxazole (**4c**) (44 mg, 0.25 mmol), 4-iodotoluene (**2a**) (273 mg, 1.25 mmol), Hybrid-Cu (30 mg, 11 mol %) and LiO*t*Bu (60 mg, 0.75 mmol) in Et_2O (0.5 mL). Purification by column chromatography on silica gel (*n*-hexane/EtOAc: 70/1) yielded **5ca** (49.1 mg, 74%) as a light yellow solid.

M. p.: 113 ºC.

^{1}H NMR (400 MHz, $CDCl_3$): δ = 8.30 – 8.25 (m, 2H), 7.94 (dd, *J* = 1.9, 0.6 Hz, 1H), 7.62 (dd, *J* = 8.6, 0.6 Hz, 1H), 7.54 (dd, *J* = 8.6, 1.9 Hz, 1H), 7.49 – 7.44 (m, 2H), 2.57 (s, 3H), 1.54 (s, 9H).

^{13}C NMR (100 MHz, $CDCl_3$): δ = 163.4 (C_q), 148.7 (C_q), 148.0 (C_q), 142.1 (C_q), 141.8 (C_q), 129.6 (CH), 127.5 (CH), 124.6 (C_q), 122.5 (CH), 116.3 (CH), 109.6 (CH), 34.9 (C_q), 31.8 (CH_3), 21.6 (CH_3).

IR (ATR): 2920, 1616, 1556, 1497, 1462, 1215, 1166, 1118, 1049, 919, 831 cm^{-1}.

MS (ESI) *m/z* (relative intensity): 266 (100) $[M+H]^+$.

HR-MS (ESI): *m/z* calcd for $C_{18}H_{20}NO$ $[M+H]^+$: 266.1542, found: 266.1540.

The spectral data were in accordance with those reported in the literature.[18]

5-Chloro-2-(*p*-tolyl)benzo[*d*]oxazole (5da)

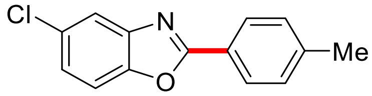

GP1 was followed using 5-chlorobenzo[d]oxazole (**4d**) (38 mg, 0.25 mmol), 4-iodotoluene (**2a**) (273 mg, 1.25 mmol), Hybrid-Cu (30 mg, 11 mol %) and LiO*t*Bu (60 mg, 0.75 mmol) in Et_2O (0.5 mL). Purification by column chromatography on silica gel (*n*-hexane/EtOAc: 70/1) yielded **5da** (31.1 mg, 51%) as a light yellow solid.

M. p.: 124 ºC.

^{1}H NMR (400 MHz, $CDCl_3$): δ = 8.27 – 8.22 (m, 2H), 7.79 (dd, J = 8.5, 0.4 Hz, 1H), 7.71 (dd, J = 2.1, 0.4 Hz, 1H), 7.48 – 7.44 (m, 3H), 2.58 (s, 3H).

^{13}C NMR (100 MHz, $CDCl_3$): δ = 163.9 (C_q), 150.8 (C_q), 142.4 (C_q), 141.0 (C_q), 130.4 (C_q), 129.7 (CH), 127.6 (CH), 125.1 (CH), 123.9 (C_q), 120.2 (CH), 111.1 (CH), 21.7 (CH_3).

IR (ATR): 2956, 2851, 1737, 1461, 1377, 1190, 1121, 1082, 716, 649, 597 cm^{-1}.

MS (ESI) *m/z* (relative intensity): 244 (100) $[M+H]^+$.

HR-MS (ESI): *m/z* calcd for $C_{14}H_{11}ClNO$ $[M+H]^+$: 244.0524, found: 244.0524.

The spectral data were in accordance with those reported in the literature.[19]

2-Phenylbenzo[*d*]oxazole (5ab)

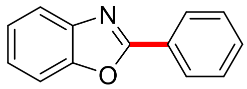

GP1 was followed using benzoxazole (**4a**) (30 mg, 0.25 mmol), iodobenzene (**2b**) (255 mg, 1.25 mmol), Hybrid-Cu (30 mg, 11 mol %) and LiO*t*Bu (60 mg, 0.75 mmol) in Et_2O (0.5 mL). Purification by column chromatography on silica gel (*n*-hexane/EtOAc: 70/1) yielded **5ab** (39.0 mg, 80%) as a light yellow solid.

M. p.: 100 ºC.

^{1}H NMR (400 MHz, $CDCl_3$): δ = 8.28 – 8.22 (m, 2H), 7.76 (ddd, J = 6.0, 3.3, 0.7 Hz, 1H), 7.57 (ddd, J = 6.0, 3.3, 0.7 Hz, 1H), 7.54 – 7.49 (m, 3H), 7.34 (dd, J = 6.0, 3.3 Hz, 2H).

^{13}C NMR (100 MHz, $CDCl_3$): δ = 163.0 (C_q), 150.8 (C_q), 142.1 (C_q), 131.5 (CH), 129.9 (CH), 127.6 (CH), 127.2 (C_q), 125.1 (CH), 124.6 (CH), 120.0 (CH), 110.6 (CH).

IR (ATR): 2922, 2020, 1973, 1555, 1474, 1263, 1182, 1057, 865, 791, 719 cm^{-1}.

MS (ESI) *m/z* (relative intensity): 196 (100) $[M+H]^+$.

HR-MS (ESI): *m/z* calcd for $C_{13}H_{10}NO$ $[M+H]^+$: 196.0755, found: 196.0756.

The spectral data were in accordance with those reported in the literature.[17]

5-Methyl-2-(*m*-tolyl)benzo[*d*]oxazole (5bh)

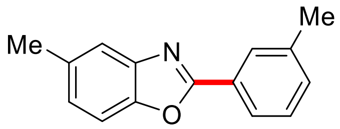

GP1 was followed using 5-methylbenzo[d]oxazole (**4b**) (33 mg, 0.25 mmol), 3-iodotoluene (**2h**) (273 mg, 1.25 mmol), Hybrid-Cu (30 mg, 11 mol %) and LiO*t*Bu (60 mg, 0.75 mmol) in Et_2O (0.5 mL). Purification by column chromatography on silica gel (*n*-hexane/EtOAc: 70/1) yielded **5bh** (43.0 mg, 77%) as a light yellow solid.

M. p.: 85 °C.

^{1}H NMR (300 MHz, $CDCl_3$): δ = 8.11 – 8.02 (m, 1H), 8.07 – 7.96 (m, 1H), 7.41 (ddd, J = 8.3, 0.3 Hz, 1H), 7.49 – 7.33 (m, 2H), 7.38 – 7.26 (m, 1H), 7.13 (ddd, J = 8.3, 1.7, 0.7 Hz, 1H), 2.47 (s, 3H), 2.44 (s, 3H).

^{13}C NMR (125 MHz, $CDCl_3$): δ = 163.2 (C_q), 148.9 (C_q), 142.2 (C_q), 138.6 (C_q), 134.3 (C_q), 132.1 (CH), 128.7 (CH), 128.0 (CH), 127.1 (C_q), 126.0 (CH), 124.6 (CH), 119.8 (CH), 109.8 (CH), 21.6 (CH_3), 21.4 (CH_3).

IR (ATR): 2919, 1613, 1483, 1423, 1143, 1055, 971, 873, 815, 631, 478 cm^{-1}.

MS (ESI) *m/z* (relative intensity): 224 (100) $[M+H]^+$, 246 (5) $[M+Na]^+$.

HR-MS (ESI): *m/z* calcd for $C_{15}H_{14}NO$ $[M+H]^+$: 224.1071, found: 224.1070.

The spectral data were in accordance with those reported in the literature.[6]

2-(*p*-Tolyl)oxazole (5ea)

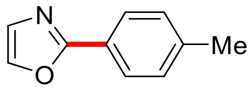

GP1 was followed using oxazole (**4e**) (17 mg, 0.25 mmol), 4-iodotoluene (**2a**) (273 mg, 1.25 mmol), Hybrid-Cu (30 mg, 11 mol %) and LiO*t*Bu (60 mg, 0.75 mmol) in Et_2O (0.5 mL). Purification by column chromatography on silica gel (*n*-hexane/EtOAc: 70/1) yielded **5ea** (24.3 mg, 61%) as a yellow oil.

^{1}H NMR (400 MHz, $CDCl_3$): δ = 7.93 – 7.89 (m, 2H), 7.66 (d, J = 0.8 Hz, 1H), 7.27 – 7.25 (m, 1H), 7.25 – 7.23 (m, 1H), 7.19 (d, J = 0.8 Hz, 1H), 2.38 (s, 3H).

^{13}C NMR (100 MHz, $CDCl_3$): δ = 162.2 (C_q), 140.6 (C_q), 138.2 (CH), 129.5 (CH), 128.2 (CH), 126.3 (CH), 124.8 (C_q), 21.5 (CH_3).

IR (ATR): 2924, 2230, 2211, 2171, 2042, 1976, 714, 686, 585, 487 cm^{-1}.

MS (ESI) *m/z* (relative intensity): 160 (100) $[M+H]^+$.

HR-MS (ESI): *m/z* calcd for $C_{10}H_{10}NO$ $[M+H]^+$: 160.0759, found: 160.0758.

The spectral data were in accordance with those reported in the literature.[22]

2,5-Diphenyloxazole (5fb)

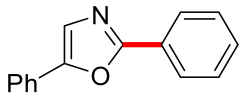

GP1 was followed using 5-phenyloxazole (**4f**) (36 mg, 0.25 mmol), iodobenzene (**2b**) (255 mg, 1.25 mmol), Hybrid-Cu (30 mg, 11 mol %) and LiO*t*Bu (60 mg, 0.75 mmol) in Et_2O (0.5 mL). Purification by column chromatography on silica gel (*n*-hexane/EtOAc: 70/1) yielded **5fb** (42.0 mg, 76%) as a light yellow solid.

M. p.: 70 ºC.

^{1}H NMR (300 MHz, $CDCl_3$): δ = 8.13 – 8.07 (m, 2H), 7.74 – 7.72 (m, 1H), 7.71 – 7.69 (m, 1H), 7.49 – 7.41 (m, 6H), 7.37 – 7.29 (m, 1H).

^{13}C NMR (125 MHz, $CDCl_3$): δ = 161.0 (C_q), 151.2 (C_q), 130.2 (CH), 128.8 (CH), 128.7 (CH), 128.3 (CH), 128.0 (C_q), 127.4 (C_q), 126.2 (CH), 124.1 (CH), 123.4 (CH).

IR (ATR): 2955, 2923, 2853, 1731, 1684, 1749, 1092, 1014, 934, 685 cm^{-1}.

MS (ESI) *m/z* (relative intensity): 222 (100) $[M+H]^+$.

HR-MS (ESI): *m/z* calcd for $C_{15}H_{12}NO$ $[M+H]^+$: 222.0913, found: 222.0913.

The spectral data were in accordance with those reported in the literature.[6]

2-(4-Chlorophenyl)-5-phenyloxazole (5ff)

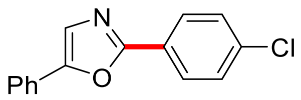

GP1 was followed using 5-phenyloxazole (**4f**) (36 mg, 0.25 mmol), 1-chloro-4-iodobenzene (**2f**) (298 mg, 1.25 mmol), Hybrid-Cu (30 mg, 11 mol %) and LiO*t*Bu (60 mg, 0.75 mmol) in Et_2O (0.5 mL). Purification by column chromatography on silica gel (*n*-hexane/EtOAc: 70/1) yielded **5ff** (50.5 mg, 79%) as a light yellow solid.

M. p.: 115 ºC.

^{1}H NMR (400 MHz, $CDCl_3$): δ = 8.05 – 7.99 (m, 2H), 7.72 – 7.67 (m, 2H), 7.47 – 7.40 (m, 5H), 7.37 – 7.30 (m, 1H).

^{13}C NMR (100 MHz, $CDCl_3$): δ = 160.2 (C_q), 151.5 (C_q), 136.4 (C_q), 129.1 (CH), 129.0 (CH), 128.6 (CH), 127.8 (C_q), 127.5 (CH), 126.0 (C_q), 124.2 (CH), 123.5 (CH).

IR (ATR): 2956, 2923, 2855, 1727, 1682, 1471, 1133, 761, 725, 383 cm^{-1}.

MS (ESI) *m/z* (relative intensity): 256 (100) $[M+H]^+$.

HR-MS (ESI): *m/z* calcd for $C_{15}H_{11}ClNO$ $[M+H]^+$: 256.0521, found: 256.0523.

The spectral data were in accordance with those reported in the literature.[6]

5-Phenyl-2-(*m*-tolyl)oxazole (5fh)

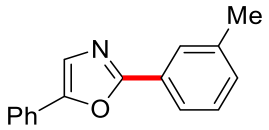

GP1 was followed using 5-phenyloxazole (**4f**) (36 mg, 0.25 mmol), 3-iodotoluene (**2h**) (273 mg, 1.25 mmol), Hybrid-Cu (30 mg, 11 mol %) and LiO*t*Bu (60 mg, 0.75 mmol) in Et_2O (0.5 mL). Purification by column chromatography on silica gel (*n*-hexane/EtOAc: 70/1) yielded **5fh** (43.5 mg, 74%) as a light yellow solid.

M. p.: 89 ºC.

^{1}H NMR (300 MHz, $CDCl_3$): δ = 7.94 – 7.87 (m, 2H), 7.74 – 7.69 (m, 2H), 7.48 – 7.39 (m, 3H), 7.39 – 7.29 (m, 2H), 7.29 – 7.24 (m, 1H), 2.43 (s, 3H).

^{13}C NMR (125 MHz, $CDCl_3$): δ = 161.3 (C_q), 151.1 (C_q), 138.6 (C_q), 131.1 (CH), 128.9 (CH), 128.7 (CH), 128.4 (CH), 128.0 (C_q), 127.3 (C_q), 126.8 (CH), 124.2 (CH), 123.4 (CH), 123.4 (CH), 21.4 (CH_3).

IR (ATR): 3055, 1886, 1499, 1231, 1099, 851, 823, 686, 609, 512 cm^{-1}.

MS (ESI) *m/z* (relative intensity): 236 (100) $[M+H]^+$, 258 (3) $[M+Na]^+$.

HR-MS (ESI): *m/z* calcd for $C_{16}H_{14}NO$ $[M+H]^+$: 236.1071, found: 236.1070.

The spectral data were in accordance with those reported in the literature.[6]

5-(4-Fluorophenyl)-2-phenyloxazole (5gb)

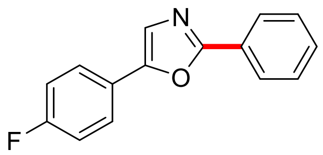

GP1 was followed using 5-(4-fluorophenyl)oxazole (**4g**) (41 mg, 0.25 mmol), iodobenzene (**2b**) (255 mg, 1.25 mmol), Hybrid-Cu (30 mg, 11 mol %) and LiO*t*Bu (60 mg, 0.75 mmol) in Et_2O (0.5 mL). Purification by column chromatography on silica gel (*n*-hexane/EtOAc: 70/1) yielded **5gb** (48.4 mg, 81%) as a light yellow solid.

M. p.: 90 ºC.

^{1}H NMR (400 MHz, $CDCl_3$): δ = 8.11 – 8.06 (m, 2H), 7.71 – 7.66 (m, 2H), 7.50 – 7.43 (m, 3H), 7.37 (s, 1H), 7.16 – 7.10 (m, 2H).

^{13}C NMR (100 MHz, $CDCl_3$): δ = 162.7 (d, $J_{C\text{-}F}$ = 249 Hz, C_q), 161.1 (C_q), 150.4 (C_q), 130.4 (CH), 128.8 (CH), 127.4 (C_q), 126.3 (CH), 126.1 (d, $J_{C\text{-}F}$ = 8 Hz, CH), 124.4 (d, $J_{C\text{-}F}$ = 3 Hz, C_q), 123.1 (d, $J_{C\text{-}F}$ = 1 Hz, CH), 116.1 (d, $J_{C\text{-}F}$ = 22 Hz, CH).

^{19}F NMR (282 MHz, $CDCl_3$): δ = –112.21 (ddd, J = 13.8, 8.7, 5.1 Hz).

IR (ATR): 2917, 1724, 1608, 1497, 1362, 1323, 1260, 1106, 963, 934, 752 cm^{-1}.

MS (ESI) *m/z* (relative intensity): 240 (100) $[M+H]^+$.

HR-MS (ESI): *m/z* calcd for $C_{15}H_{11}FNO$ $[M+H]^+$: 240.0818, found: 240.0819.

The spectral data were in accordance with those reported in the literature.[6]

5-(Benzo[*d*][1,3]dioxol-5-yl)-2-(*p*-tolyl)oxazole (5ha)

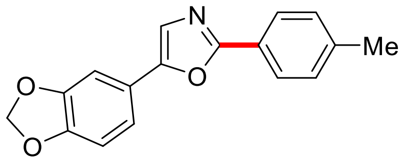

GP1 was followed using 5-(benzo[*d*][1,3]dioxol-5-yl)oxazole (**4h**) (47 mg, 0.25 mmol), 4-iodotoluene (**2a**) (273 mg, 1.25 mmol), Hybrid-Cu (30 mg, 11 mol %) and LiO*t*Bu (60 mg, 0.75 mmol) in Et_2O (0.5 mL). Purification by column chromatography on silica gel (*n*-hexane/EtOAc: 70/1) yielded **5ha** (58.7 mg, 84%) as a light yellow solid.

M. p.: 125 ºC.

^{1}H NMR (400 MHz, $CDCl_3$): δ = 7.98 – 7.90 (m, 2H), 7.28 – 7.26 (m, 2H), 7.25 – 7.23 (m, 1H), 7.20 (dd, J = 8.1, 1.7 Hz, 1H), 7.14 (dd, J = 1.7, 0.4 Hz, 1H), 6.85 (dd, J = 8.1, 0.5 Hz, 1H), 5.99 (s, 2H), 2.39 (s, 3H).

^{13}C NMR (100 MHz, $CDCl_3$): δ = 160.9 (C_q), 150.8 (C_q), 148.2 (C_q), 147.8 (C_q), 140.5 (C_q), 129.5 (CH), 126.1 (CH), 124.7 (C_q), 122.3 (C_q), 122.2 (CH), 118.2 (CH), 108.8 (CH), 104.8 (CH), 101.3 (CH_2), 21.5 (CH_3).

IR (ATR): 2898, 1726, 1683, 1609, 1545, 1363,1287, 1129, 1062, 963, 776 cm^{-1}.

MS (ESI) *m/z* (relative intensity): 280 (100) $[M+H]^+$, 302 (10) $[M+Na]^+$.

HR-MS (ESI): *m/z* calcd for $C_{17}H_{14}NO_3$ $[M+H]^+$: 280.0967, found: 280.0968.

5-(Benzo[*d*][1,3]dioxol-5-yl)-2-phenyloxazole (5hb)

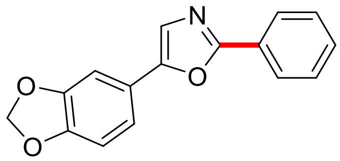

GP1 was followed using 5-(benzo[*d*][1,3]dioxol-5-yl)oxazole (**4h**) (47 mg, 0.25 mmol), iodobenzene (**2b**) (255 mg, 1.25 mmol), Hybrid-Cu (30 mg, 11 mol %) and LiO*t*Bu (60 mg, 0.75 mmol) in Et_2O (0.5 mL). Purification by column chromatography on silica gel (*n*-hexane/EtOAc: 70/1) yielded **5hb** (57.7 mg, 87%) as a light yellow solid.

M. p.: 136 ºC.

^{1}H NMR (400 MHz, $CDCl_3$): δ = 8.10 – 8.03 (m, 2H), 7.49 – 7.38 (m, 3H), 7.28 (s, 1H), 7.21 (dd, J = 8.1, 1.7 Hz, 1H), 7.15 (dd, J = 1.7, 0.4 Hz, 1H), 6.86 (dd, J = 8.1, 0.4 Hz, 1H), 5.99 (s, 2H).

^{13}C NMR (100 MHz, $CDCl_3$): δ = 160.6 (C_q), 151.1 (C_q), 148.2 (C_q), 147.9 (C_q), 130.2 (CH), 128.8 (CH), 127.4 (C_q), 126.1 (CH), 122.3 (CH), 122.2 (C_q), 118.3 (CH), 108.8 (CH), 104.8 (CH), 101.4 (CH_2).

IR (ATR): 2865, 1605, 1349, 1276, 1109, 1088, 964, 854, 774, 589, 422 cm^{-1}.

MS (ESI) *m/z* (relative intensity): 266 (100) $[M+H]^+$.

HR-MS (ESI): *m/z* calcd for $C_{16}H_{12}NO_3$ $[M+H]^+$: 266.0817, found: 266.0815.

The spectral data were in accordance with those reported in the literature.[6]

1-Methyl-2-(*p*-tolyl)-1*H*-benzo[*d*]imidazole (7aa)

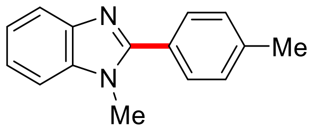

1) **GP2** was followed using 1-methyl-1*H*-benzo[*d*]imidazole (**6a**) (33 mg, 0.25 mmol), 4-iodotoluene (**2a**) (273 mg, 1.25 mmol), Hybrid-Cu (30 mg, 11 mol %) and K_3PO_4 (159 mg, 0.75 mmol) in THF (0.5 mL). Purification by column chromatography on silica gel (*n*-hexane/EtOAc: 4/1) yielded **7aa** (35.0 mg, 63%) as a light yellow solid.
2) **GP3** was followed using 1-methyl-1*H*-benzo[*d*]imidazole (**6a**) (33 mg, 0.25 mmol), 4-iodotoluene (**2a**) (273 mg, 1.25 mmol), Hybrid-Cu (30 mg, 11 mol %) and Cs_2CO_3 (244 mg, 0.75 mmol) in Et_2O (0.5 mL). Purification by column chromatography on silica gel (*n*-hexane/EtOAc: 4/1) yielded **7aa** (41.1 mg, 74%) as a light yellow solid.

M. p.: 125 ºC.

^{1}H NMR (400 MHz, $CDCl_3$) δ 7.80 (ddd, J = 5.4, 2.4, 0.7 Hz, 1H), 7.65 (t, J = 1.9 Hz, 1H), 7.63 (t, J = 1.9 Hz, 1H), 7.35 (ddd, J = 5.4, 2.4, 0.7H), 7.31 – 7.33 (m, 1H), 7.30 – 7.31 (m, 1H), 7.28 (m, 2H), 3.83 (s, 3H), 2.42 (s, 3H).

^{13}C NMR (100 MHz, $CDCl_3$) δ 153.9 (C_q), 142.9 (C_q), 139.8 (C_q), 136.5 (C_q), 129.3 (CH), 129.3 (C_q), 127.3 (CH), 122.6 (CH), 122.3 (CH), 119.7 (CH), 109.5 (CH), 31.6 (CH_3), 21.4 (CH_3).

IR (ATR): 3419, 3402, 2919, 1936, 1613, 1459, 1381, 1276, 1005, 822, 748 cm^{-1}.

MS (ESI) *m/z* (relative intensity): 223 (60) $[M+H]^+$, 245 (40) $[M+Na]^+$.

HR-MS (ESI): *m/z* calcd for $C_{15}H_{15}N_2$ $[M+H]^+$: 223.1237, found: 223.1235.

The spectral data were in accordance with those reported in the literature.[24]

1-Methyl-2-phenyl-1*H*-benzo[*d*]imidazole (7ab)

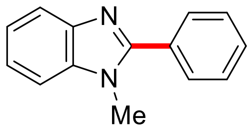

1) **GP2** was followed using 1-methyl-1*H*-benzo[*d*]imidazole (**6a**) (33 mg, 0.25 mmol), iodobenzene (**2b**) (255 mg, 1.25 mmol), Hybrid-Cu (30 mg, 11 mol %) and K_3PO_4 (159 mg, 0.75 mmol) in THF (0.5 mL). Purification by column chromatography on silica gel (*n*-hexane/EtOAc: 4/1) yielded **7ab** (36.4 mg, 70%) as a light yellow solid.
2) **GP3** was followed using 1-methyl-1*H*-benzo[*d*]imidazole (**6a**) (33 mg, 0.25 mmol), iodobenzene (**2b**) (255 mg, 1.25 mmol), Hybrid-Cu (30 mg, 11 mol %) and Cs_2CO_3 (244 mg, 0.75 mmol) in Et_2O (0.5 mL). Purification by column chromatography on silica gel (*n*-hexane/EtOAc: 4/1) yielded **7ab** (41.1 mg, 79%) as a light yellow solid.

M. p.: 96 ºC.

^{1}H NMR (400 MHz, $CDCl_3$) δ 7.81 (ddd, J = 5.4, 2.4, 0.7 Hz, 1H), 7.76 – 7.72 (m, 2H), 7.54 – 7.47 (m, 3H), 7.36 (ddd, J = 5.4, 2.4, 0.7 Hz, 1H), 7.32 – 7.27 (m, 2H), 3.83 (s, 3H).

^{13}C NMR (100 MHz, $CDCl_3$) δ 153.7 (C_q), 142.9 (C_q), 136.5 (C_q), 130.1 (C_q), 129.7 (CH), 129.4 (CH), 128.6 (CH), 122.7 (CH), 122.4 (CH), 119.8 (CH), 109.6 (CH), 31.6 (CH_3).

IR (ATR): 3059, 2924, 2159, 1469, 1442, 1382, 1328, 1006, 744, 698 cm^{-1}.

MS (ESI) *m/z* (relative intensity): 210 (100) $[M+H]^+$.

HR-MS (ESI): *m/z* calcd for $C_{14}H_{13}N_2$ $[M+H]^+$: 210.1074, found: 210.1073.

The spectral data were in accordance with those reported in the literature.[25]

2-(4-Methoxyphenyl)-1-methyl-1*H*-benzo[*d*]imidazole (7ac)

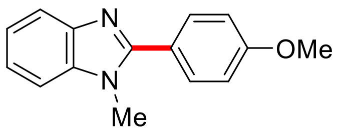

1) **GP2** was followed using 1-methyl-1*H*-benzo[*d*]imidazole (**6a**) (33 mg, 0.25 mmol), 4-iodoanisole (**2c**) (293 mg, 1.25 mmol), Hybrid-Cu (30 mg, 11 mol %) and K_3PO_4 (159 mg, 0.75 mmol) in THF (0.5 mL). Purification by column chromatography on silica gel (*n*-hexane/EtOAc: 4/1) yielded **7ac** (36.3 mg, 61%) as a light yellow solid.
2) **GP3** was followed using 1-methyl-1*H*-benzo[*d*]imidazole (**6a**) (33 mg, 0.25 mmol), iodobenzene (**2b**) (255 mg, 1.25 mmol), Hybrid-Cu (30 mg, 11 mol %) and Cs_2CO_3 (244 mg, 0.75 mmol) in Et_2O (0.5 mL). Purification by column chromatography on silica gel (*n*-hexane/EtOAc: 4/1) yielded **7ac** (47.1 mg, 79%) as a light yellow solid.

M. p.: 118 ºC.

^{1}H NMR (400 MHz, $CDCl_3$) δ 7.79 (ddd, J = 4.9, 2.3, 0.7 Hz, 1H), 7.70 (dd, J = 2.9, 2.3 Hz, 1H), 7.68 (dd, J = 2.9, 2.3 Hz, 1H), 7.35 (ddd, J = 4.9, 2.3, 0.7 Hz, 1H), 7.29 (dd, J = 2.9, 2.3 Hz, 1H) 7.28 (dd, J = 2.9, 2.3 Hz, 1H), 7.03 (dd, J = 2.9, 2.3 Hz, 1H), 7.02 (dd, J = 2.9, 2.3 Hz, 1H).3.86 (s, 3H), 3.83 (s, 3H).

^{13}C NMR (100 MHz, $CDCl_3$) δ 160.7 (C_q), 142.9 (C_q), 136.5 (C_q), 130.8 (C_q), 122.5 (CH), 122.5 (C_q), 122.5 (CH), 122.3 (CH), 119.6 (CH), 114.1 (CH), 109.5 (CH), 55.4 (CH_3), 31.7 (CH_3).

IR (ATR): 2938, 2837, 1706, 1611, 1461, 1250, 1177, 1025, 838, 745 cm^{-1}.

MS (ESI) *m/z* (relative intensity): 239 (80) $[M+H]^+$, 261 (20) $[M+Na]^+$

HR-MS (ESI): *m/z* calcd for $C_{15}H_{15}N_2O$ $[M+H]^+$: 239.1182, found: 239.1180.

The spectral data were in accordance with those reported in the literature.[24]

2-(4-Fluorophenyl)-1-methyl-1*H*-benzo[*d*]imidazole (7ae)

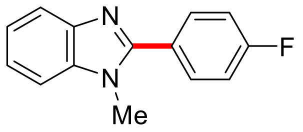

1) **GP2** was followed using 1-methyl-1*H*-benzo[*d*]imidazole (**6a**) (33 mg, 0.25 mmol), 1-fluoro-4-iodobenzene (**2e**) (278 mg, 1.25 mmol), Hybrid-Cu (30 mg, 11 mol %) and K_3PO_4 (159 mg, 0.75 mmol) in THF (0.5 mL). Purification by column chromatography on silica gel (*n*-hexane/EtOAc: 4/1) yielded **7ae** (36.7 mg, 65%) as a light yellow solid.
2) **GP3** was followed using 1-methyl-1*H*-benzo[*d*]imidazole (**6a**) (33 mg, 0.25 mmol), 1-fluoro-4-iodobenzene (**2e**) (278 mg, 1.25 mmol), Hybrid-Cu (30 mg, 11 mol %) and Cs_2CO_3 (244 mg, 0.75 mmol) in Et_2O (0.5 mL). Purification by column chromatography on silica gel (*n*-hexane/EtOAc: 4/1) yielded **7ae** (39.6 mg, 70%) as a light yellow solid

M. p.: 98 ºC.

^{1}H NMR (400 MHz, $CDCl_3$) δ 7.80 (ddd, J = 4.5, 2.0, 0.7 Hz, 1H), 7.75 (ddd, J = 5.1, 3.0, 2.3 Hz, 1H), 7.73 (ddd, J = 5.1, 3.0, 2.3 Hz, 1H), 7.37 (ddd, J = 4.5, 2.0, 0.7, 1H), 7.33 – 7.28 (m, 2H), 7.25 – 7.17 (m, 2H), 3.83 (s, 3H).

^{13}C NMR (100 MHz, $CDCl_3$) δ 163.6 (d, $J_{C\text{-}F}$ = 250.5 Hz, C_q), 152.8 (C_q), 142.8 (C_q), 136.5 (C_q), 131.4 (d, $J_{C\text{-}F}$ = 8.4 Hz, CH), 126.3 (d, $J_{C\text{-}F}$ = 3.4 Hz, C_q), 122.9 (CH), 122.5 (CH), 119.8 (CH), 115.9 (d, $J_{C\text{-}F}$ = 22.0 Hz, CH)), 109.9 (CH), 109.6 (CH), 31.6 (CH_3).

^{19}F NMR (282 MHz, $CDCl_3$) δ –110.60 (ddd, J = 13.6, 8.5, 5.2 Hz).

IR (ATR): 3059, 2951, 2158, 1645, 1606, 1461, 1225, 1097, 843, 744 cm^{-1}.

MS (ESI) *m/z* (relative intensity): 227 (70) $[M+H]^+$, 249 (30) $[M+Na]^+$.

HR-MS (ESI): *m/z* calcd for $C_{14}H_{12}FN_2$ $[M+H]^+$: 227.0985, found: 227.0983.

The spectral data were in accordance with those reported in the literature.[24]

1-(4-(1-Methyl-1*H*-benzo[*d*]imidazol-2-yl)phenyl)ethan-1-one (7ar)

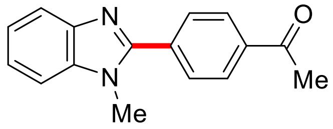

GP2 was followed using 1-methyl-1*H*-benzo[*d*]imidazole (**6a**) (33 mg, 0.25 mmol), 1-(4-iodophenyl)ethan-1-one (**2r**) (308 mg, 1.25 mmol), Hybrid-Cu (30 mg, 11 mol %) and K_3PO_4 (159 mg, 0.75 mmol) in THF (0.5 mL). Purification by column chromatography on silica gel (*n*-hexane/EtOAc: 4/1) yielded **7ar** (29.4 mg, 47%) as a light yellow solid.

M. p.: 153 ºC.

^{1}H NMR (400 MHz, $CDCl_3$) δ 8.10 (dd, J = 2.3, 1.8 Hz, 1H), 8.8 (dd, J = 2.3, 1.8 Hz, 1H), 7.89 (dd, J = 2.3, 1.8 Hz, 1H), 7.87 (dd, J = 2.3, 1.8 Hz, 1H), 7.83 – 7.81 (m, 1H), 7.41 – 7.39 (m, 1H), 7.33 (ddd, J = 6.7, 4.1, 1.8 Hz, 2H), 3.88 (s, 3H), 2.66 (s, 3H).

^{13}C NMR (100 MHz, $CDCl_3$) δ 197.5 (C_q), 152.4 (C_q), 143.0 (C_q), 137.6 (C_q), 136.7 (C_q), 134.6 (C_q), 129.6 (CH), 128.6 (CH), 123.3 (CH), 122.8 (CH), 120.1 (CH), 109.7 (CH), 31.8 (CH_3), 26.8 (CH_3).

IR (ATR): 2932, 1682, 1608, 1463, 1355, 1262, 1011, 834, 744, 604 cm^{-1}.

MS (ESI) *m/z* (relative intensity): 251 (80) $[M+H]^+$, 273 (20) $[M+H]^+$.

HR-MS (ESI): *m/z* calcd for $C_{16}H_{15}N_2O$ $[M+H]^+$: 251.1181, found: 251.1179.

Methyl 4-(1-methyl-1*H*-benzo[*d*]imidazol-2-yl)benzoate (7as)

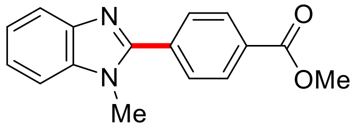

GP2 was followed using 1-methyl-1*H*-benzo[*d*]imidazole (**6a**) (33 mg, 0.25 mmol), methyl 4-iodobenzoate (**2s**) (328 mg, 1.25 mmol), Hybrid-Cu (30 mg, 11 mol %) and K_3PO_4 (159 mg, 0.75 mmol) in THF (0.5 mL). Purification by column chromatography on silica gel (*n*-hexane/EtOAc: 4/1) yielded **7as** (41.9 mg, 63%) as a light yellow solid.

M. p.: 120 ºC.

^{1}H NMR (400 MHz, $CDCl_3$) δ 8.20 (dd, J = 2.3, 1.7 Hz, 1H), 8.18 (dd, J = 2.3, 1.7 Hz, 1H), 7.87(dd, J = 2.3, 1.7 Hz, 1H), 7.85 (dd, J = 2.3, 1.7 Hz, 1H), 7.82 (ddd, J = 4.1, 2.3, 0.8 Hz, 1H), 7.40 (ddd, J = 4.1, 2.3, 0.8 Hz, 1H), 7.32 (ddd, J = 6.6, 4.1, 1.7 Hz, 2H), 3.95 (s, 3H), 3.88 (s, 3H).

^{13}C NMR (100 MHz, $CDCl_3$) δ 166.5 (C_q), 152.5 (C_q), 143.0 (C_q), 136.7 (C_q), 134.5 (C_q), 131.1 (C_q), 129.9 (CH), 129.4 (CH), 123.2 (CH), 122.7 (CH), 120.1 (CH), 109.7 (CH), 52.4 (CH_3), 31.8 (CH_3).

IR (ATR): 2950, 1719, 1612, 1530, 1460, 1274, 1016, 964, 743 cm^{-1}.

MS (ESI) *m/z* (relative intensity): 267 (90) $[M+H]^+$, 289 (10) $[M+H]^+$.

HR-MS (ESI): *m/z* calcd for $C_{16}H_{15}N_2O_2$ $[M+H]^+$: 267.1132, found: 267.1130.

1-Methyl-2-(*m*-tolyl)-1*H*-benzo[*d*]imidazole (7ah)

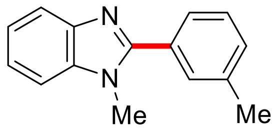

GP2 was followed using 1-methyl-1*H*-benzo[*d*]imidazole (**6a**) (33 mg, 0.25 mmol), 1-iodo-3-methylbenzene (**2h**) (273 mg, 1.25 mmol), Hybrid-Cu (30 mg, 11 mol %) and K_3PO_4 (159 mg, 0.75 mmol) in THF (0.5 mL). Purification by column chromatography on silica gel (*n*-hexane/EtOAc: 4/1) yielded **7ah** (34.5 mg, 62%) as a light yellow solid.

M. p.: 119 ºC.

^{1}H NMR (400 MHz, $CDCl_3$) δ 7.80 (ddd, J = 5.2, 2.3, 0.9 Hz, 1H), 7.61 – 7.60 (m, 1H), 7.50 – 7.49 (m, 1H), 7.41 (t, J = 7.8 Hz, 1H), 7.39 – 7.37 (ddd, J = 5.2, 2.3, 0.9 Hz, 1H), 7.32 – 7.27 (m, 3H), 3.84 (s, 3H), 2.43 (s, 3H).

^{13}C NMR (100 MHz, $CDCl_3$) δ 153.9 (C_q), 142.9 (C_q), 138.6 (C_q), 136.5 (C_q), 130.4 (CH), 130.2 (CH), 130.1 (C_q), 128.4 (CH), 126.3 (CH), 122.6 (CH), 122.3 (CH), 119.8 (CH), 109.5 (CH), 31.6 (CH_3), 21.4 (CH_3).

IR (ATR): 3050, 2920, 1609, 1589, 1456, 1325, 1256, 793, 742, 659 cm^{-1}.

MS (ESI) *m/z* (relative intensity): 223 (100) [M+H]$^+$.

HR-MS (ESI): *m/z* calcd for $C_{15}H_{14}N_2$ [M+H]$^+$: 223.1236, found: 223.1230.

The spectral data were in accordance with those reported in the literature.[24]

1-Methyl-2-(*p*-tolyl)-1*H*-benzo[*d*]imidazole (7aj)

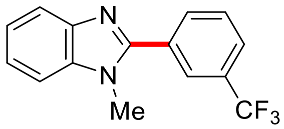

GP2 was followed using 1-methyl-1*H*-benzo[*d*]imidazole (**6a**) (33 mg, 0.25 mmol), 1-iodo-3-(trifluoromethyl)benzene (**2j**) (340 mg, 1.25 mmol), Hybrid-Cu (30 mg, 11 mol %) and K_3PO_4 (159 mg, 0.75 mmol) in THF (0.5 mL). Purification by column chromatography on silica gel (*n*-hexane/EtOAc: 4/1) yielded **7aj** (40.7 mg, 59%) as a light yellow solid.

M. p.: 141 ºC.

^{1}H NMR (400 MHz, $CDCl_3$) δ 8.06 – 8.04 (m, 1H), 7.97 – 7.93 (m, 1H), 7.82 (ddd, *J* = 3.6, 1.6, 0.8 Hz, 1H), 7.77 – 7.73 (m, 1H), 7.68 – 7.62 (m, 1H), 7.40 (ddd, *J* = 3.6, 1.6, 0.8 Hz, 1H), 7.34 (ddd, *J* = 6.4, 3.6, 1.6 Hz, 2H), 3.87 (s, 3H).

^{13}C NMR (100 MHz, $CDCl_3$) δ 152.0 (C_q), 142.9 (C_q), 136.6 (C_q), 132.5 (CH), 131.3 (d, $J_{C\text{-}F}$ = 32.8 Hz, C_q), 131.1 (C_q), 129.2 (CH), 126.6 (q, $J_{C\text{-}F}$ = 3.8 Hz, CH), 125.1 (C_q), 123.7 (d, $J_{C\text{-}F}$ = 273.6 Hz, CH), 123.3 (CH), 122.8 (CH), 120.1 (CH), 109.7 (CH), 31.7 (CH_3).

^{19}F NMR (282 MHz, $CDCl_3$) δ -62.73 (s).

IR (ATR): 3108, 3058, 2926, 1460, 1324, 1249, 1122, 1060, 743, 650 cm^{-1}.

MS (ESI) *m/z* (relative intensity): 277 (100) $[M+H]^+$.

HR-MS (ESI): *m/z* calcd for $C_{15}H_{11}F_3N_2$ $[M+H]^+$: 277.0949, found: 277.0947.

2-(2-Methoxyphenyl)-1-methyl-1*H*-benzo[*d*]imidazole (7ao)

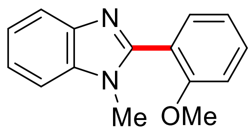

GP2 was followed using 1-methyl-1*H*-benzo[*d*]imidazole (**6a**) (33 mg, 0.25 mmol), 1-iodo-2-methoxybenzene (**2o**) (293 mg, 1.25 mmol), Hybrid-Cu (30 mg, 11 mol %) and K_3PO_4 (159 mg, 0.75 mmol) in THF (0.5 mL). Purification by column chromatography on silica gel (*n*-hexane/EtOAc: 4/1) yielded **7ao** (16.6 mg, 33%) as a light yellow oil.

M. p.: 97 ºC.

^{1}H NMR (400 MHz, $CDCl_3$) δ 7.80 (ddd, J = 3.5, 1.7, 0.7 Hz, 1H), 7.56 (dd, J = 7.5, 1.7 Hz, 1H), 7.47 (ddd, J = 8.3, 7.5, 1.7 Hz, 1H), 7.37 (ddd, J = 3.5, 1.7, 0.7 Hz, 1H), 7.28 (ddd, J = 7.5, 3.5, 1.7 Hz, 2H), 7.08 (td, J = 7.5, 0.7 Hz, 1H), 7.00 (dd, J = 8.3, 0.7 Hz, 1H), 3.79 (s, 3H), 3.63 (s, 3H).

^{13}C NMR (100 MHz, $CDCl_3$) δ 157.4 (C_q), 152.0 (C_q), 143.0 (C_q), 136.0 (C_q), 132.2 (CH), 131.5 (CH), 122.3 (CH), 121.9 (CH), 120.9 (CH), 119.7 (CH), 119.5 (C_q), 110.9 (CH), 109.3 (CH), 55.5 (CH_3), 30.8 (CH_3).

IR (ATR): 3059, 2941, 2837, 1458, 1437, 1251, 1117, 1201, 743, 640 cm^{-1}.

MS (ESI) *m/z* (relative intensity): 239 (100) $[M+H]^+$.

HR-MS (ESI): *m/z* calcd for $C_{15}H_{15}N_2O$ $[M+H]^+$: 239.1184, found: 239.1182.

The spectral data were in accordance with those reported in the literature.[25]

(3*S*,8*S*,9*S*,10*R*,13*R*,14*S*,17*R*)-10,13-Dimethyl-17-[(*R*)-6-methylheptan-2-yl]-2,3,4,7,8,9,10,11,12,13,14,15,16,17-tetradecahydro-1*H*-cyclopenta[*a*]phenanthren-3-yl 4-(1-methyl-1*H*-benzo[*d*]imidazol-2-yl)benzoate (7at)

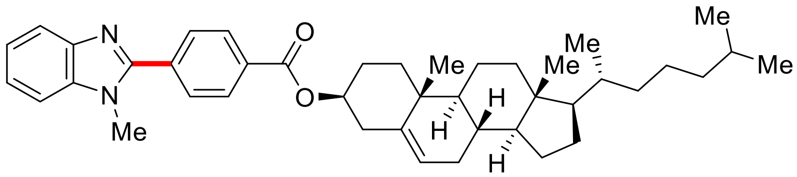

GP2 was followed using 1-methyl-1*H*-benzo[*d*]imidazole (**6a**) (33 mg, 0.25 mmol), (3*S*,8*S*,9*S*,10*R*,13*R*,14*S*,17*R*)-10,13-dimethyl-17-[(*R*)-6-methyl heptan-2-yl]-2,3,4,7,8,9,10,11,12,13,14,15,16,17-tetradecahydro-1*H*-cyclopenta[*a*]phe nanthren-3-yl 4-iodobenzoate (**2t**) (770 mg, 1.25 mmol), Hybrid-Cu (30 mg, 11 mol %) and K_3PO_4 (159 mg, 0.75 mmol) in THF (0.5 mL). Purification by column chromatography on silica gel (*n*-hexane/EtOAc: 4/1) yielded **7at** (63.6 mg, 41%) as a white solid.

M. p.: 192 ºC.

^{1}H NMR (400 MHz, $CDCl_3$) δ 8.20 (dd, J = 2.1, 1.5 Hz, 1H), 8.17 (dd, J = 2.1, 1.5 Hz, 1H), 7.86 (dd, J = 2.1, 1.5 Hz, 1H), 7.84 (dd, J = 2.1, 1.5 Hz, 1H), 7.83 – 7.81 (m, 1H), 7.40 (ddd, J = 6.3, 2.1, 0.8 Hz, 1H), 7.30 (ddd, J = 6.3, 3.8, 1.5 Hz, 2H), 5.42 (d, J = 4.5 Hz, 1H), 4.90 (m, 1H), 3.88 (s, 3H), 2.49 (d, J = 7.7 Hz, 2H), 2.07 – 1.92 (m, 4H), 1.88 – 1.62 (m, 3H), 1.62 – 1.42 (m, 5H), 1.34 – 1.30 (m, 3H), 1.27 – 1.16 (m, 4H), 1.12 – 1.08 (m, 3H), 1.07 – 0.95 (m, 7H), 0.91 (d, J = 6.5 Hz, 3H), 0.86 (d, J = 1.8 Hz, 3H), 0.84 (d, J = 1.8 Hz, 3H), 0.68 (s, 3H).

^{13}C NMR (100 MHz, $CDCl_3$) δ 165.4 (C_q), 152.6 (C_q), 143.0 (C_q), 139.5 (C_q), 136.7 (C_q), 134.3 (C_q), 131.8 (C_q), 129.8 (CH), 129.3 (CH), 123.2 (CH), 122.9 (CH), 122.7 (CH), 120.1 (CH), 109.7 (CH),
75.0 (CH), 56.7 (CH), 56.1 (CH), 50.0 (CH), 42.3 (C_q), 39.7 (CH_2), 39.5 (CH_2), 38.2 (CH_2), 37.0 (CH_3), 36.7 (CH_2), 36.2 (CH_2), 35.8 (CH), 31.9 (CH_2), 31.9 (CH), 31.8 (CH_3), 28.2 (C_q), 28.0 (CH), 27.9 (CH_2), 24.3 (CH_2), 23.8 (CH_2), 22.8 (CH_2), 22.6 (CH_3), 21.1 (CH_2), 19.4 (CH_3), 18.7 (CH_3), 11.9 (CH_3).

IR (ATR): 2936, 2867, 2248, 1674, 1714, 1465, 1273, 1119, 1016, 736 cm^{-1}.

MS (ESI) *m/z* (relative intensity): 621 (100) $[M+H]^+$.

HR-MS (ESI): *m/z* calcd for $C_{42}H_{57}N_2O_2$ $[M+H]^+$: 621.4412, found: 621.4415.

2-(4-{[(1*S*,2*S*,5*R*)-2-Isopropyl-5-methylcyclohexyl]oxy}phenyl)-1-methyl-1*H*-benzo[*d*]imidazole (7au)

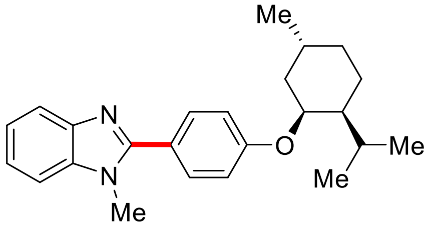

GP3 was followed using 1-methyl-1*H*-benzo[*d*]imidazole (**6a**) (33 mg, 0.25 mmol), 1-iodo-4-{[(1*S*,2*S*,5*R*)-2-isopropyl-5-methylcyclohexyl]oxy}benzene (**2u**) (448 mg, 1.25 mmol), Hybrid-Cu (30 mg, 11 mol %) and Cs_2CO_3 (244 mg, 0.75 mmol) in Et_2O (0.5 mL). Purification by column chromatography on silica gel (*n*-hexane/EtOAc: 4/1) yielded **7au** (48.9 mg, 87%) as a light yellow solid.

M. p.: 137 ºC.

^{1}H NMR (400 MHz, $CDCl_3$) δ 7.78 (ddd, *J* = 5.3, 2.3, 0.7 Hz, 1H), 7.68 (dd, *J* = 2.9, 2.3 Hz, 1H), 7.66 (dd, *J* = 2.9, 2.3 Hz, 1H), 7.36 (ddd, *J* = 5.3, 2.3, 0.7 Hz, 1H), 7.28 (ddd, *J* = 4.3, 2.3, 0.7 Hz, 2H), 7.02 (dd, *J* = 2.9, 2.3 Hz, 1H), 7.00 (dd, *J* = 2.9, 2.3 Hz, 1H), 4.71 (q, *J* = 2.6 Hz, 1H), 3.85 (s, 3H), 2.12 (dd, *J* = 14.0, 3.3 Hz, 1H), 1.83 – 1.61 (m, 4H), 1.61 – 1.51 (m, 1H), 0.97 (d, *J* = 6.7, 3H), 0.93 (d, *J* = 6.7 Hz, 3H), 0.86 (d, *J* = 6.7 Hz, 3H), 0.82 (d, *J* = 6.6 Hz, 3H).

^{13}C NMR (100 MHz, $CDCl_3$) δ 159.6 (C_q), 154.0 (C_q), 143.0 (C_q), 136.6 (C_q), 130.8 (CH), 122.4 (CH), 122.2 (CH), 121.9 (C_q), 119.5 (CH), 115.7 (CH), 109.4 (CH), 73.5 (CH), 47.7 (CH), 37.6 (CH_2), 34.9 (CH_2), 31.7 (CH_3), 29.3 (CH), 26.2 (CH), 24.8 (CH_2), 22.3 (CH), 21.0 (CH), 20.8 (CH).

IR (ATR): 2922, 2867, 1609, 1460, 1436, 1246, 1172, 962, 836, 741 cm^{-1}.

MS (ESI) *m/z* (relative intensity): 363 (100) $[M+H]^+$.

HR-MS (ESI): *m/z* calcd for $C_{24}H_{31}N_2O$ $[M+H]^+$: 363.2435, found: 363.2431.

References

1. J. Roger, F. Pozgan, H. Doucet, *J. Org. Chem.* **2009**, *74*, 1179–1186.
2. F. Besselièvre, F. Mahuteau-Betzer, D. S. Grierson, S. Piguel, *J. Org. Chem.* **2008**, *73*, 3278–3280.
3. F. Wu, Y. Feng, C. W. Jones, *ACS Catal*. **2014**, *4*, 1365–1375.
4. J. B. Ernst, C. Schwermann, G. Yokota, M. Tada, S. Muratsugu, N. L. Doltsinis, F. Glorius, *J. Am. Chem. Soc.* **2017**, *139*, 9144–9147.
5. P. Gandeepan, J. Koeller, K. Korvorapun, J. Mohr, L. Ackermann, *Angew. Chem. Int. Ed*. **2019**, *58*, 9820–9825.
6. J. R. Bolton, M. I. Stefan, P.-S. Shaw, K. R. Lykke, *J. Photochem. Photobiol. A* **2011**, *222*, 166–169.
7. M. C. Biesinger, L. W. M. Lau, A. R. Gerson, R. S. C. Smart, *Appl. Surf. Sci.* **2010**, *257*, 887–898.
8. J. Crêpellière, P. L. Popa, N. Bahlawane, R. Leturcq, F. Werner, S. Siebentritt, D. Lenoble, *J. Mater. Chem. C* **2016**, *4*, 4278–4287
9. J. Huang, J. Ying Chen, C. J. Borths, K. D. Baucom, R. D. Larsen, M. M. Faul, *J. Am. Chem. Soc*. **2010**, *132*, 3674–3675.
10. F. Yang, J. Koeller, L. Ackermann, *Angew. Chem. Int. Ed.* **2016**, *55*, 4759–4762.
11. X.-Q. Zhu, M.-T. Zhang, A. Yu, C.-H. Wang, J.-P. Cheng, *Org. Lett*. **2013**, *15*, 1598–1601
12. Q. Song, Q. Feng, M. Zhou, *Org. Lett*. **2013**, *15*, 5990–5993.
13. J. Liu, Q. Gui, Z. Yang, Z. Tan, R. Guo, J.-C. Shi, *Synthesis*, **2013**, *45*, 943–951.
14. S. K. Alla, P. Sadhu, T. Punniyamurthy, *J. Org. Chem.*, **2014**, *79*, 7502–7511.
15. D. Yang, K. Yan, W. Wei, L. Tian, Y. Shuai, R. Li, J. You, H. Wang, *Asian J. Org. Chem.* **2014**, *3*, 969–973.
16. K. Gopalaiah, S. N. Chandrudua, *RSC Adv.*, **2015**, *5*, 5015–5023.
17. G. Naresh, R. Kant, T. Narender, *J. Org. Chem.,* **2014**, *79*, 3821–3829.
18. L. Yang, J. Yuan, P. Mao, Q. Guoa, *RSC Advances*, **2015**, *5*, 107601–107607.
19. P. H. Tran, A.-H. T. Hanga, *RSC Advances*, **2018**, *8*, 11127–11133.
20. M. J. Iglesias, A. Prieto, M. C. Nicasio, *Org. Lett.*, **2012**, *14*, 4318–4321.
21. B. Liu, Z. Wang, N. Wu, M. Li, J. You, J. Lan, *Chem, Eur, J.* **2012**, *18*, 1599–1603.
22. X. Li, C. Li, B. Yin, C. Li, P. Liu, J. Li, Z. Shi, *Chem. Asian J.* **2013**, *8*, 1408–1411.
23. S. Ray, P. Das, B, Banerjee, A. Bhaumikb, C. Mukhopadhyay, *RSC Advances*,

2015, *5*, 72745–72754.

24. Z.-S. Gu, W.-W. Chen, L.-X. Shao, *J. Org. Chem.* **2014**, *79*, 5806–5811.
25. W. Zhang, Q. Zeng, X. Zhang, Y. Tian, Y. Yue, Y. Guo, Z. Wang, *J. Org. Chem.* **2011**, *76*, 4741–4745.

^{1}H NMR, ^{13}C NMR and ^{19}F NMR Spectra

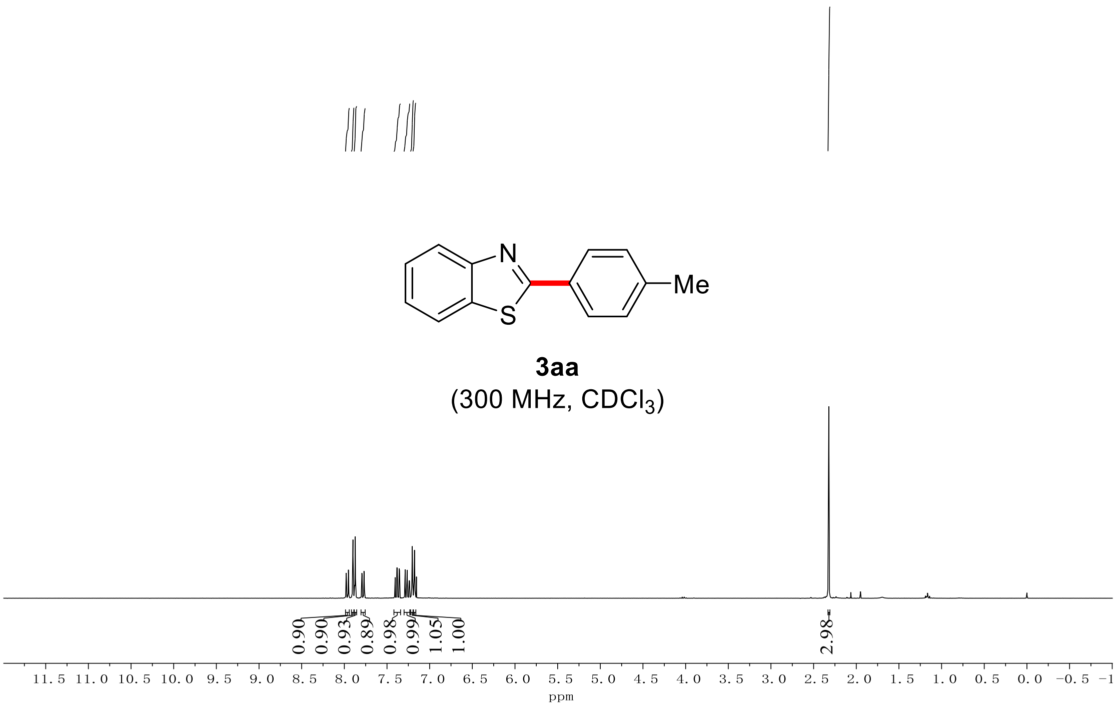

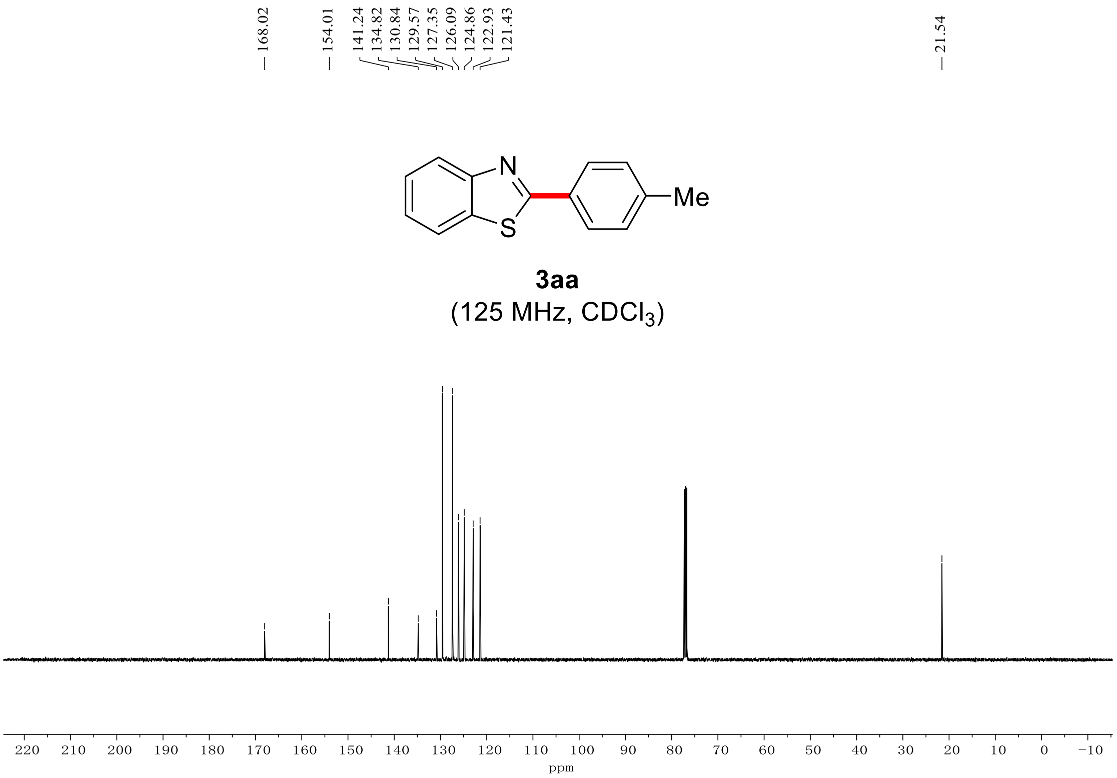

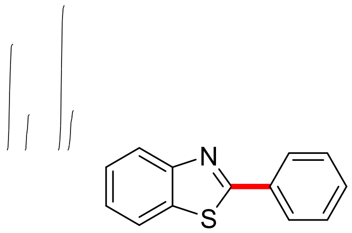

3ab
(500 MHz, $CDCl_3$)

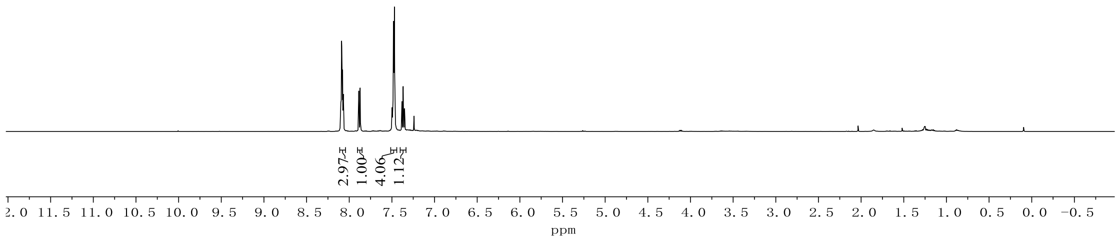

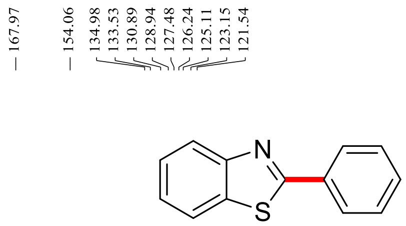

3ab
(125 MHz, $CDCl_3$)

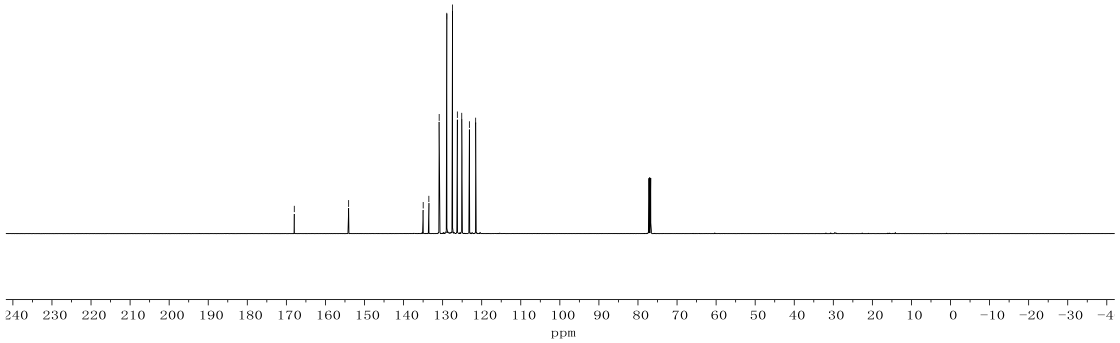

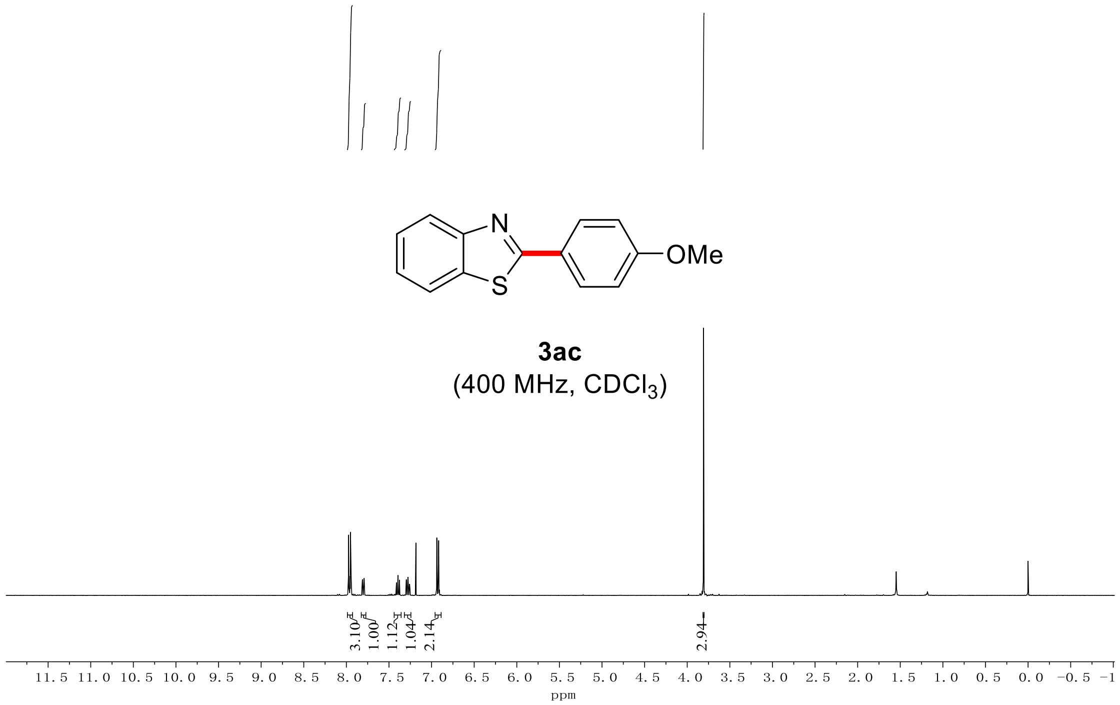
N
S
OMe
3ac
(400 MHz, CDCl$_3$)
3.10
1.00
1.12
1.04
2.14
2.94
11.5 11.0 10.5 10.0 9.5 9.0 8.5 8.0 7.5 7.0 6.5 6.0 5.5 5.0 4.5 4.0 3.5 3.0 2.5 2.0 1.5 1.0 0.5 0.0 −0.5 −1
ppm

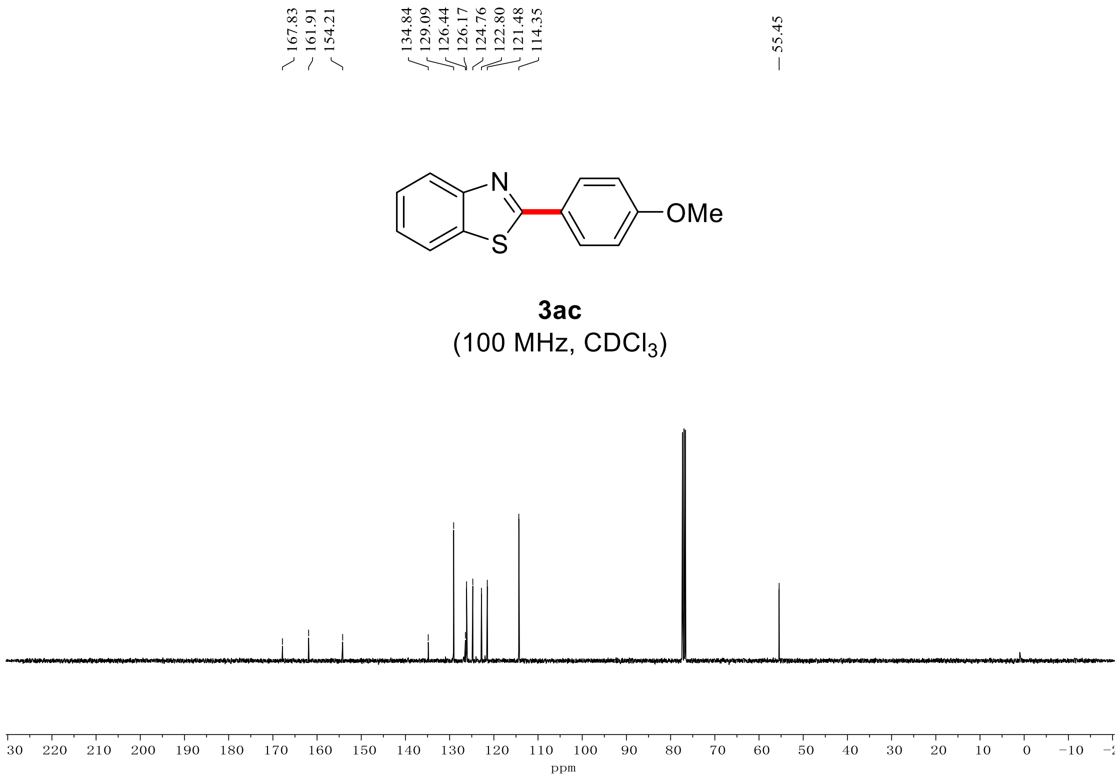
167.83
161.91
154.21
134.84
129.09
126.44
126.17
124.76
122.80
121.48
114.35
55.45
N
S
OMe
3ac
(100 MHz, CDCl$_3$)
30 220 210 200 190 180 170 160 150 140 130 120 110 100 90 80 70 60 50 40 30 20 10 0 −10
ppm

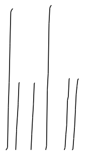

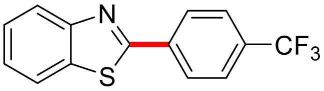

3ad
(400 MHz, $CDCl_3$)

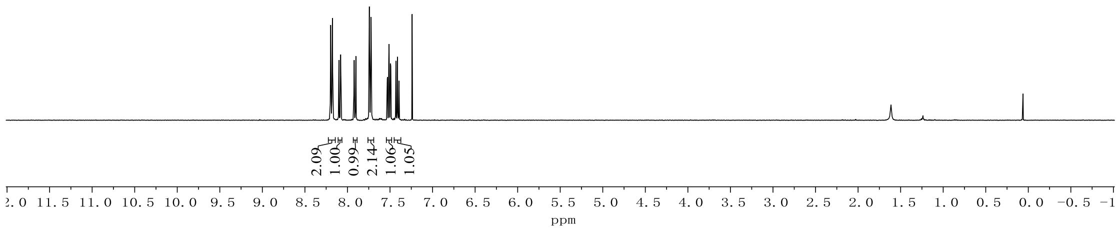

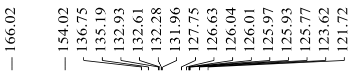

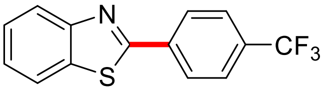

3ad
(100 MHz, $CDCl_3$)

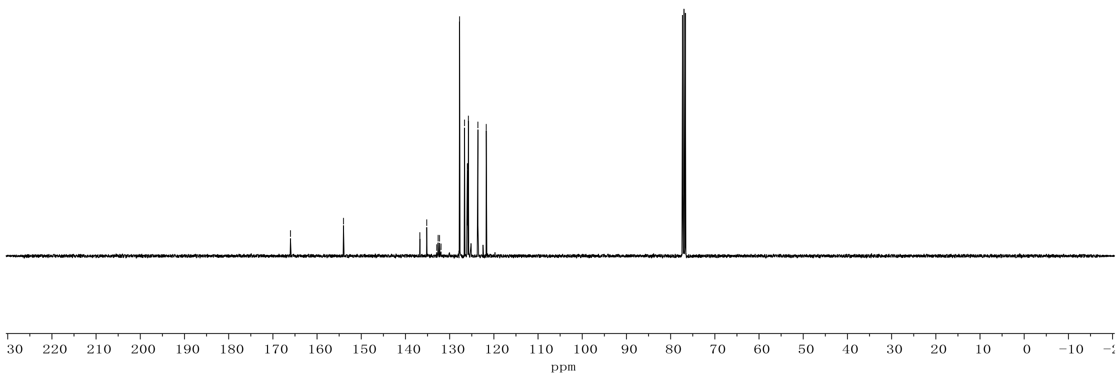

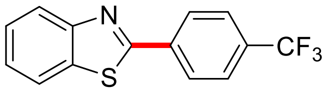

3ad

(282 MHz, $CDCl_3$)

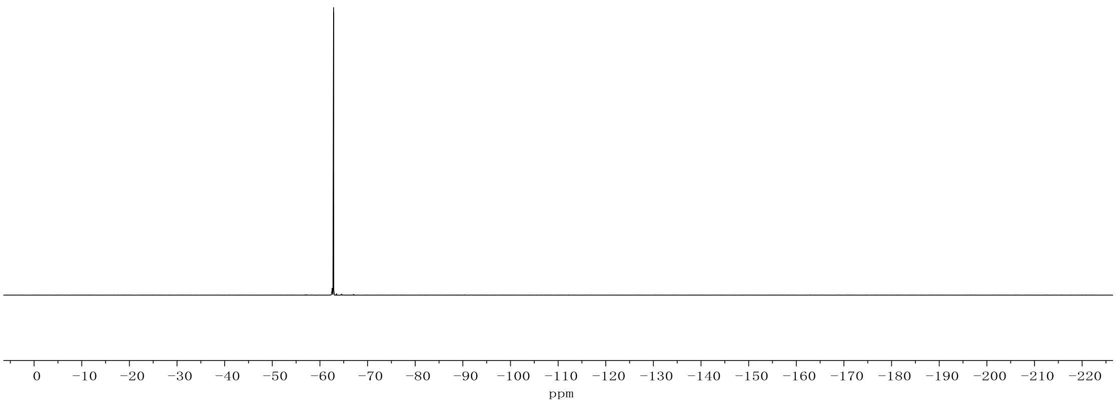

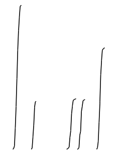

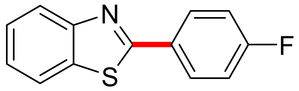

3ae
(500 MHz, $CDCl_3$)

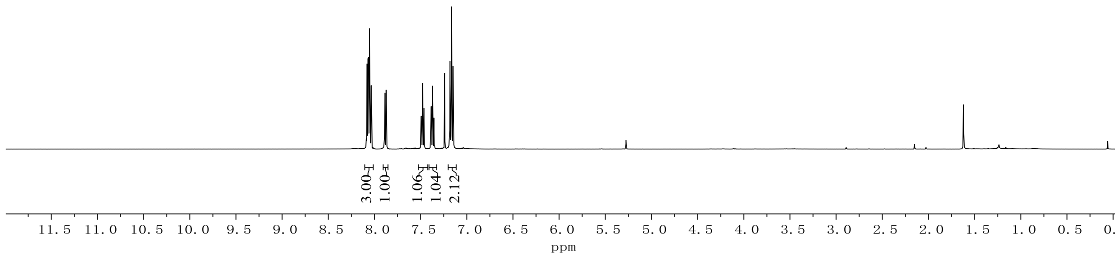

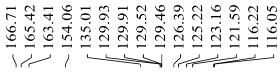

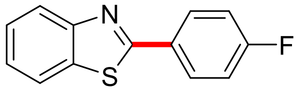

3ae
(125 MHz, $CDCl_3$)

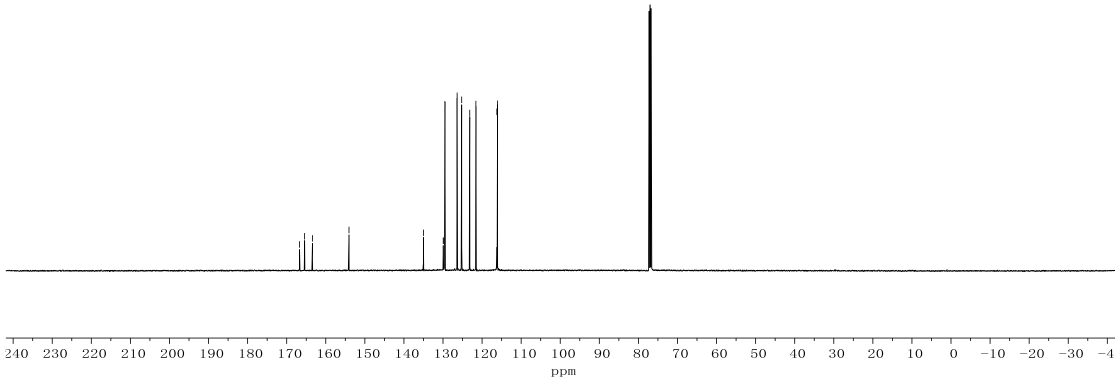

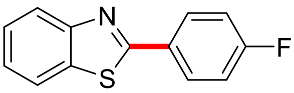

3ae
(282 MHz, $CDCl_3$)

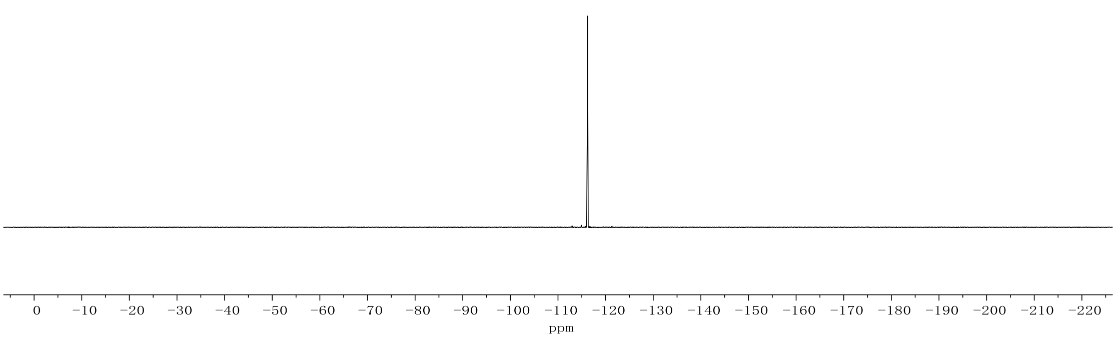

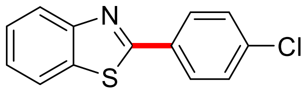

3af
(500 MHz, $CDCl_3$)

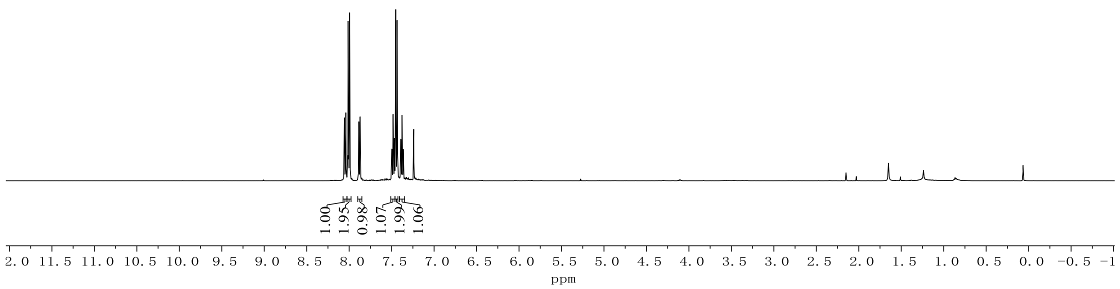

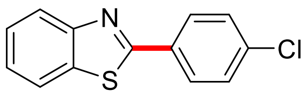

3af
(125 MHz, $CDCl_3$)

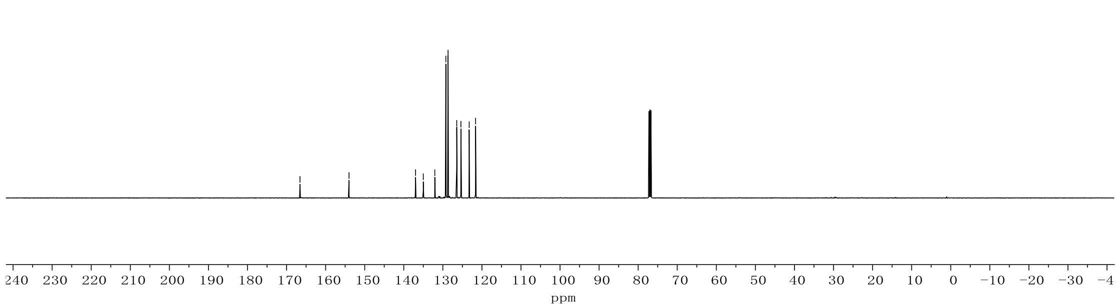

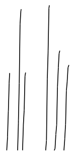

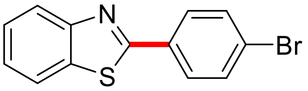

3ag
(500 MHz, $CDCl_3$)

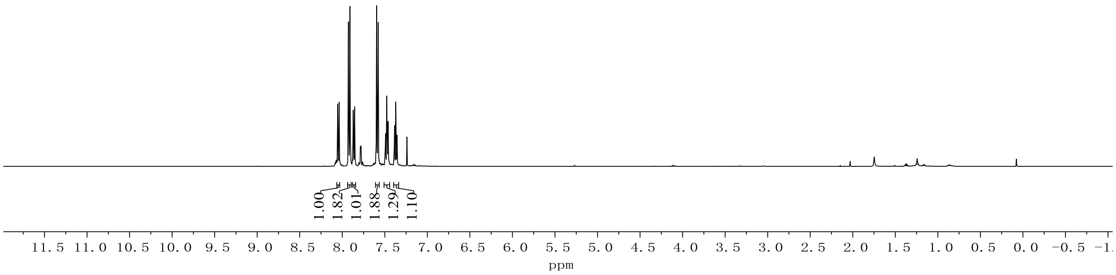

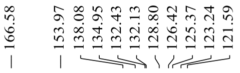

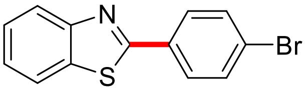

3ag
(125 MHz, $CDCl_3$)

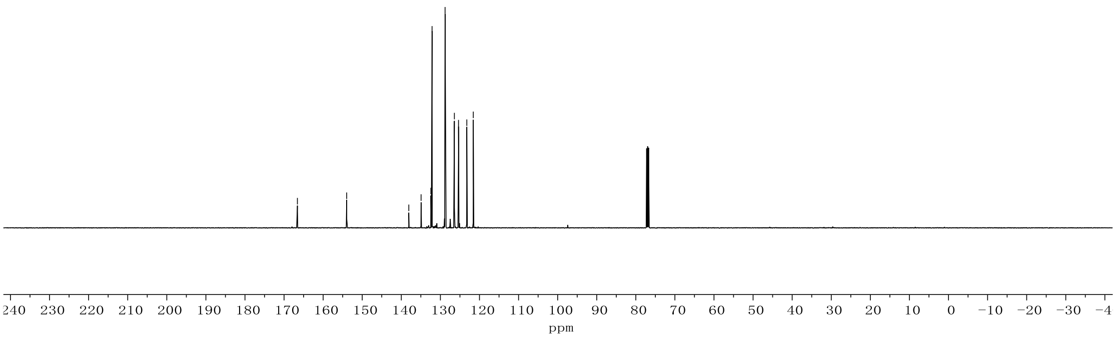

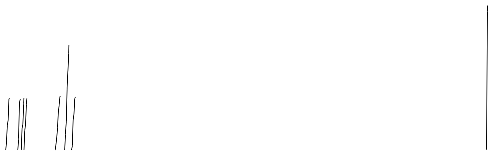

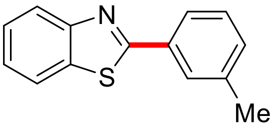

3ah
(400 MHz, $CDCl_3$)

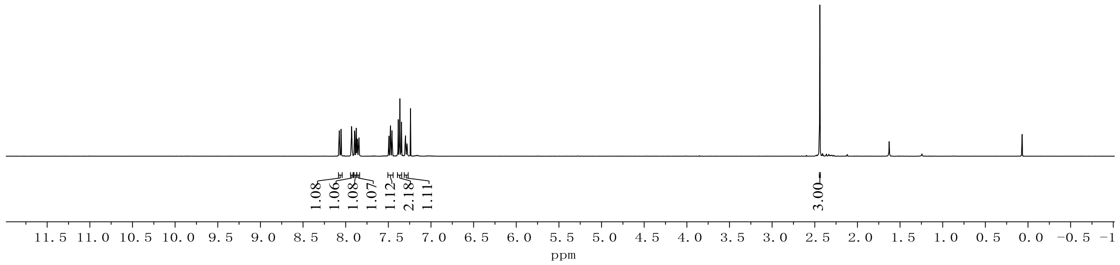

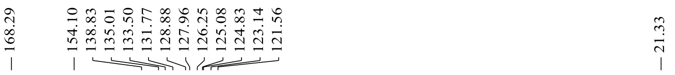

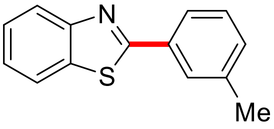

3ah
(100 MHz, $CDCl_3$)

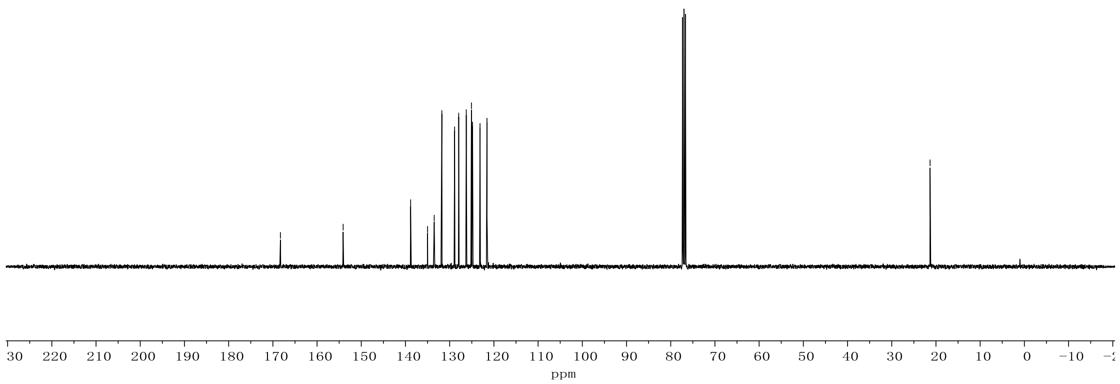

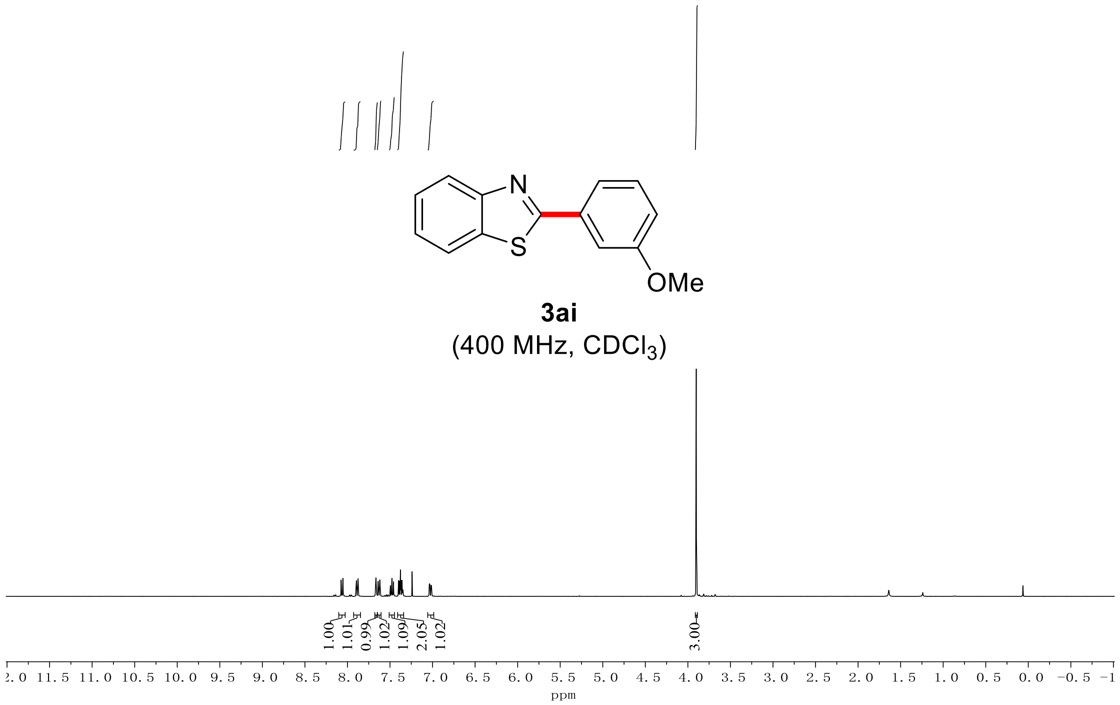

3ai
(400 MHz, $CDCl_3$)

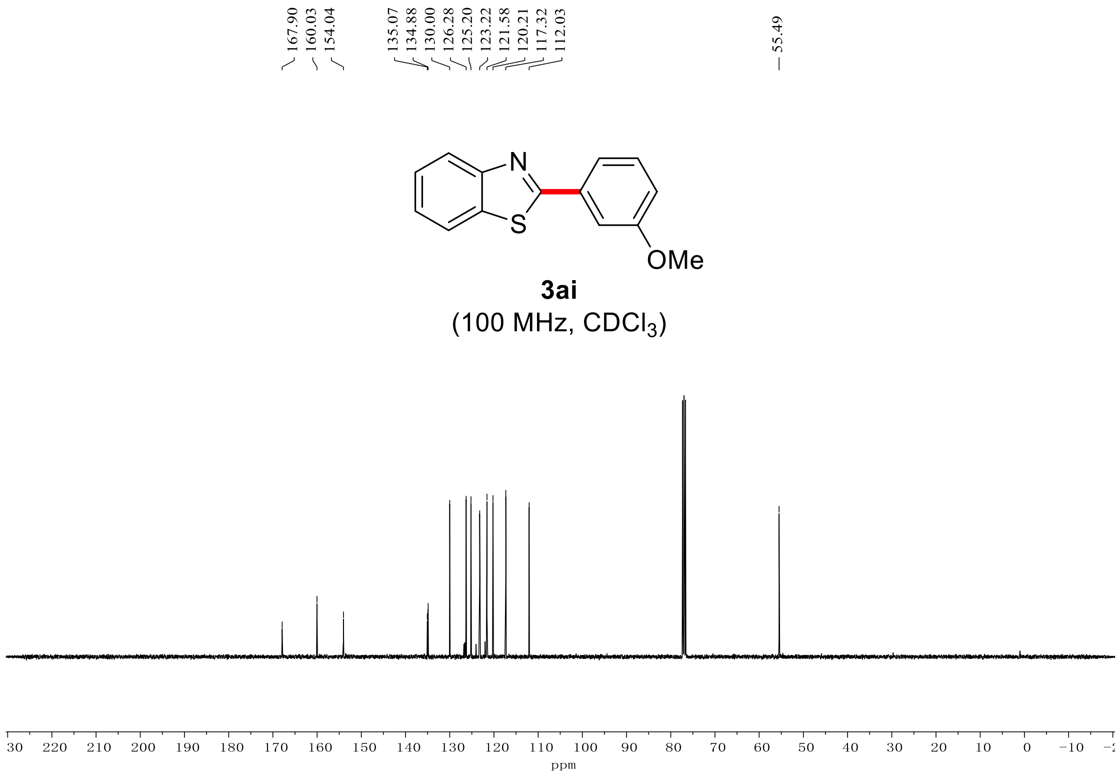

3ai
(100 MHz, $CDCl_3$)

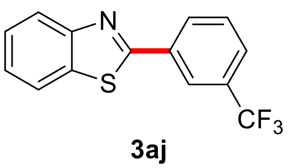

3aj
(400 MHz, $CDCl_3$)

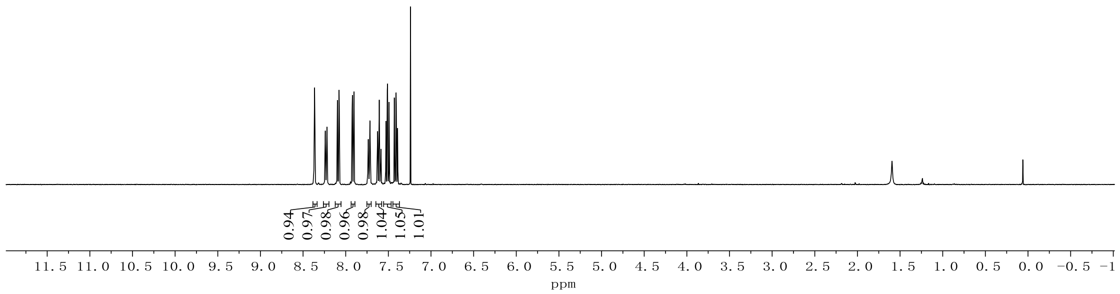

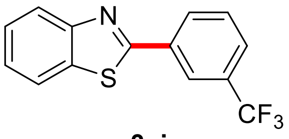

3aj
(100 MHz, $CDCl_3$)

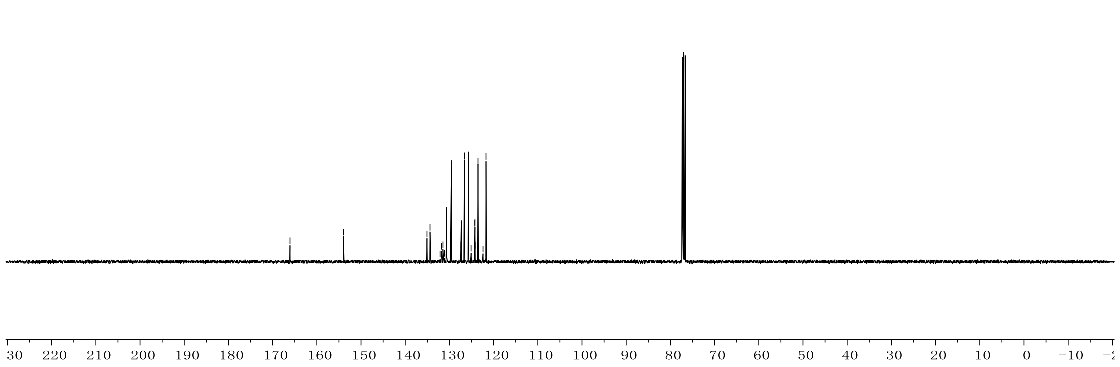

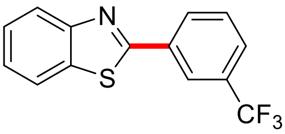

3aj
(282 MHz, $CDCl_3$)

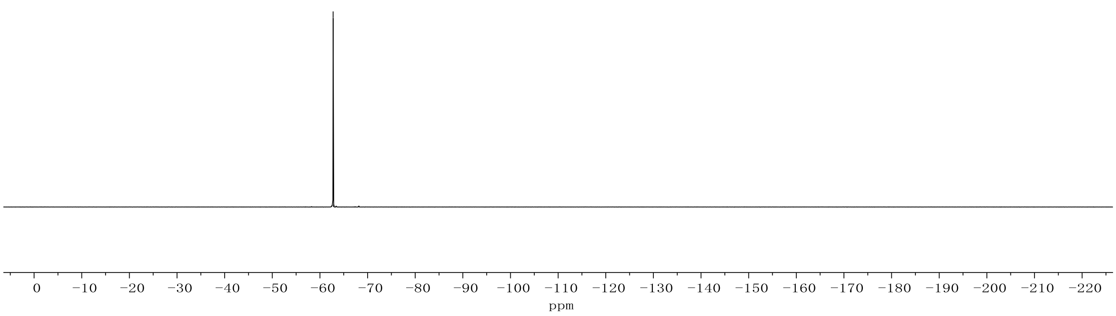

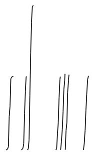

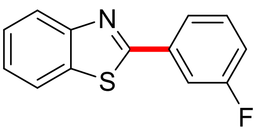

3ak
(500 MHz, $CDCl_3$)

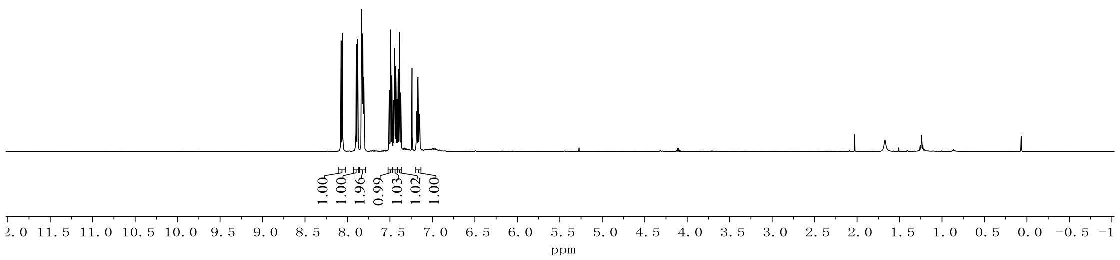

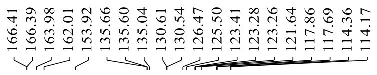

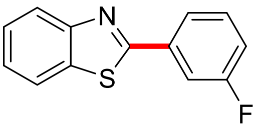

3ak
(125 MHz, $CDCl_3$)

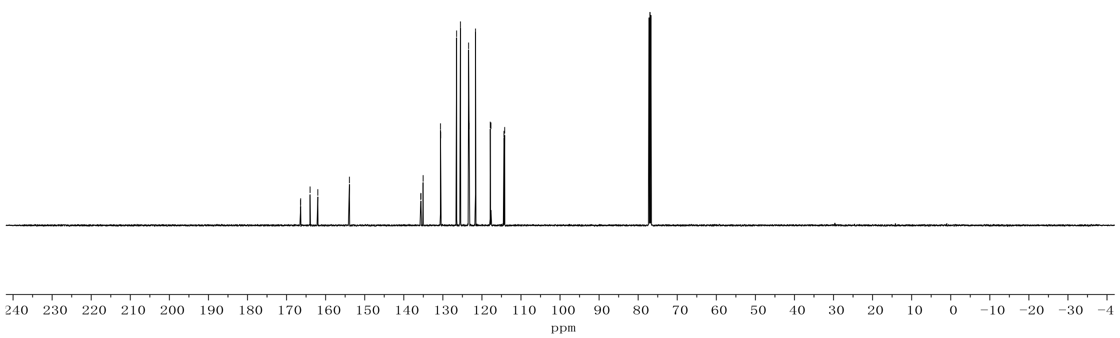

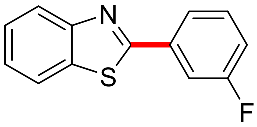

3ak
(282 MHz, $CDCl_3$)

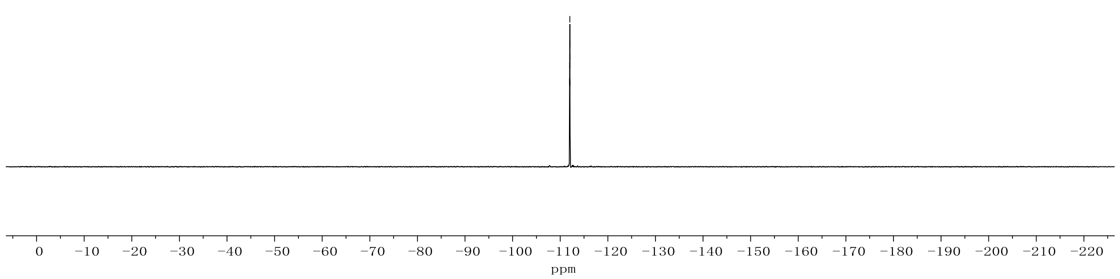

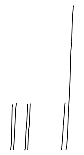

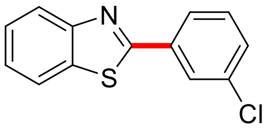

3al
(500 MHz, $CDCl_3$)

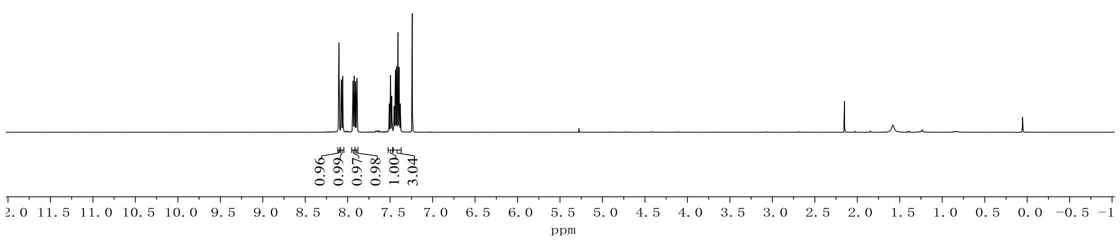

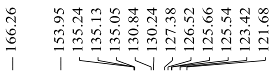

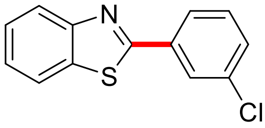

3al
(125 MHz, $CDCl_3$)

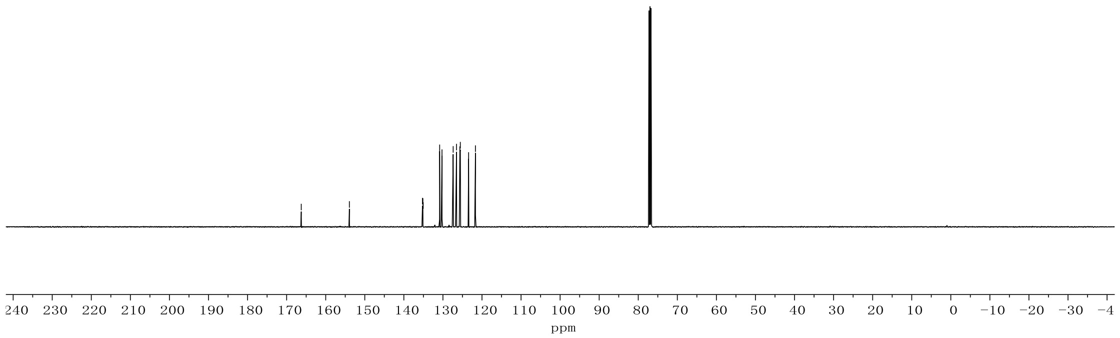

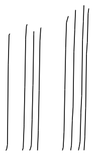

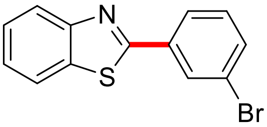

3am
(500 MHz, $CDCl_3$)

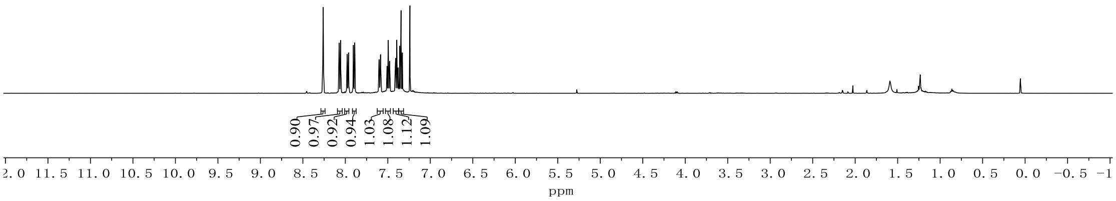

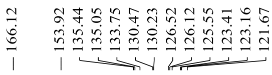

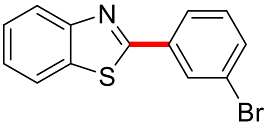

3am
(125 MHz, $CDCl_3$)

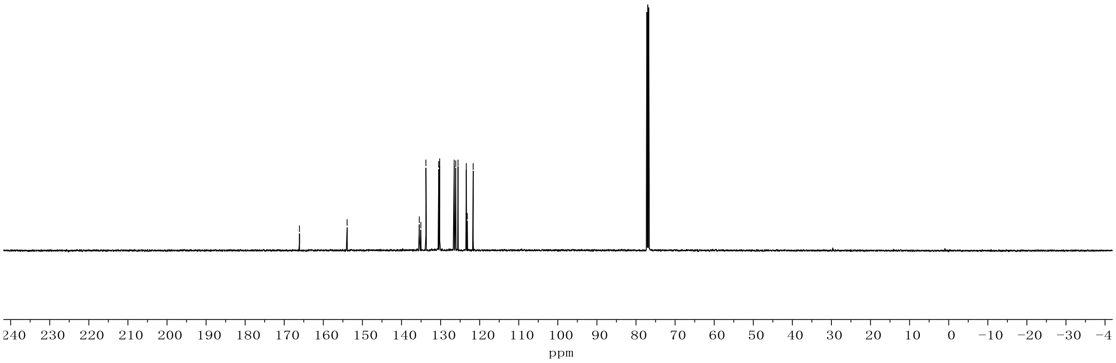

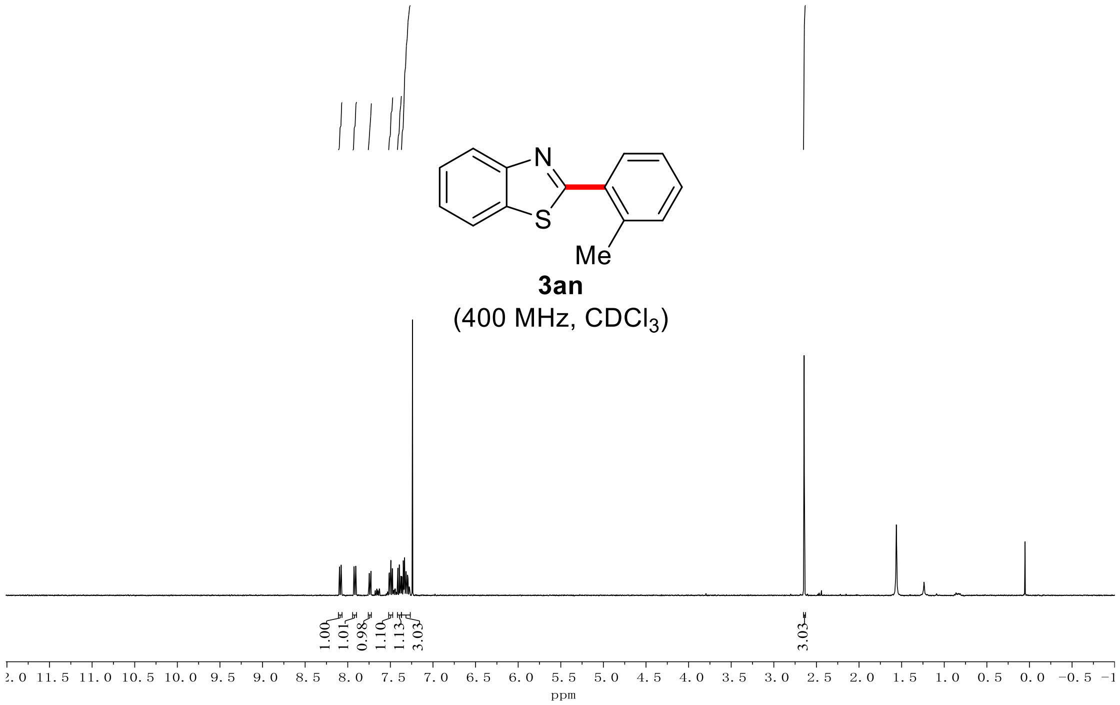
N
S
Me
3an
(400 MHz, CDCl3)
1.00
1.01
0.98
1.10
1.13
3.03
3.03
ppm

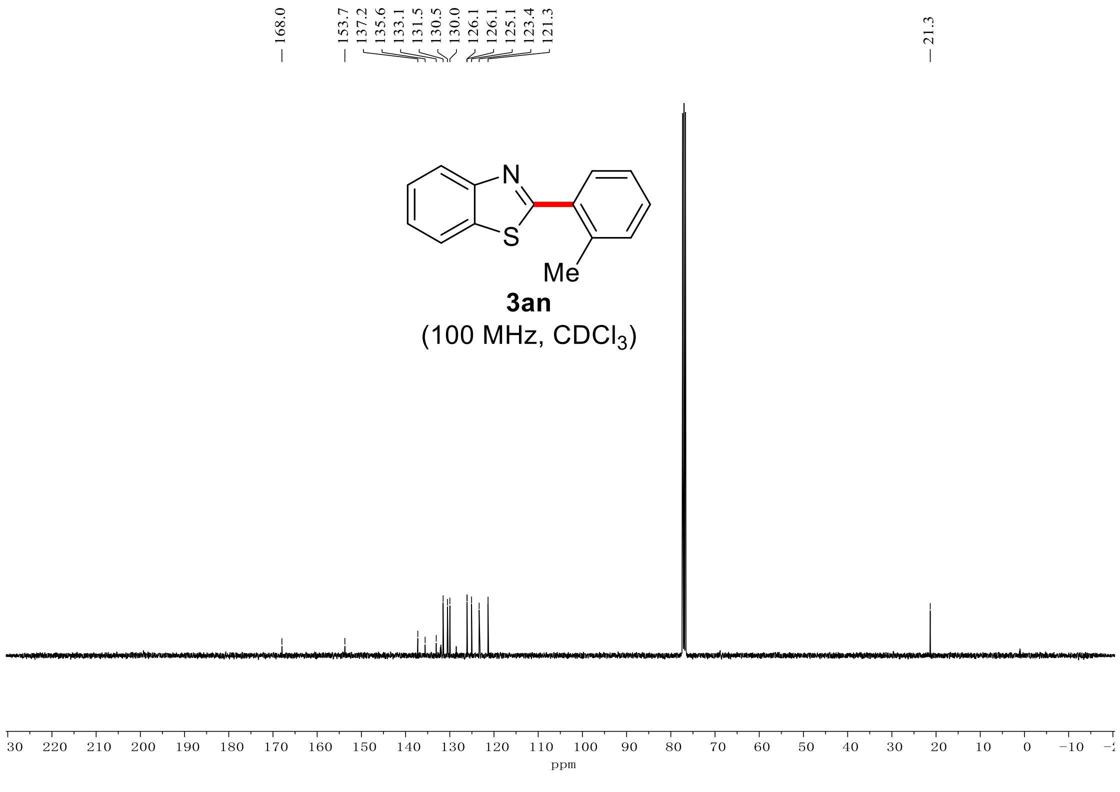
168.0
153.7
137.2
135.6
133.1
131.5
130.5
130.0
126.1
126.1
125.1
123.4
121.3
21.3
N
S
Me
3an
(100 MHz, CDCl3)
ppm

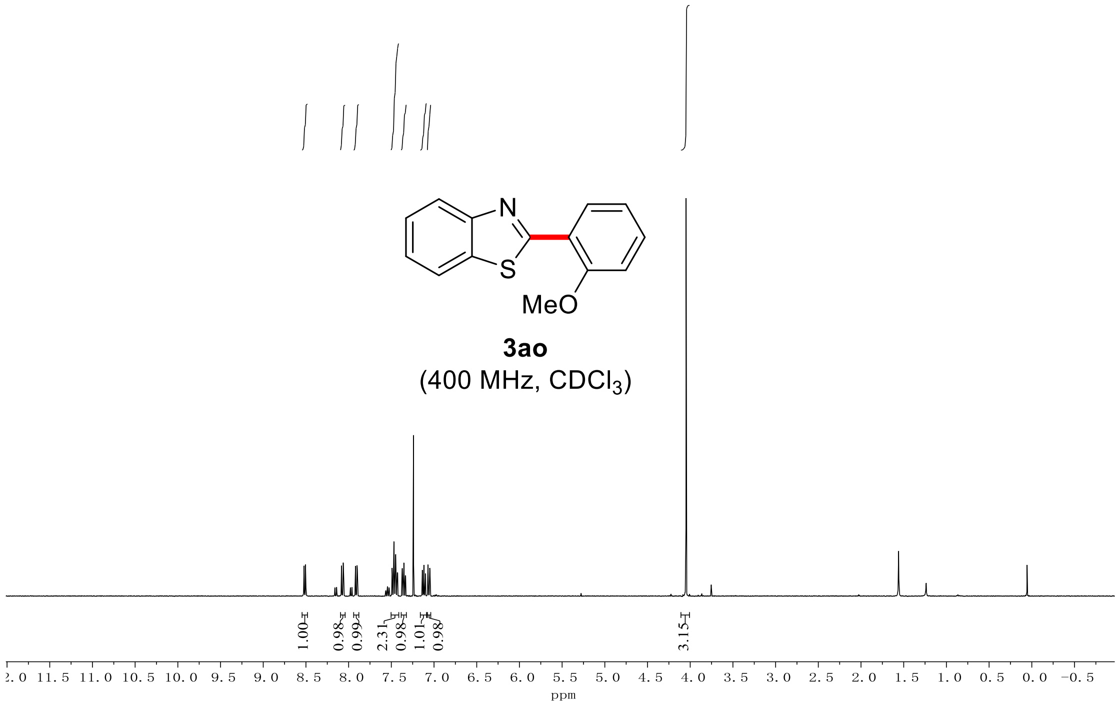
N
S
MeO
3ao
(400 MHz, CDCl3)
ppm

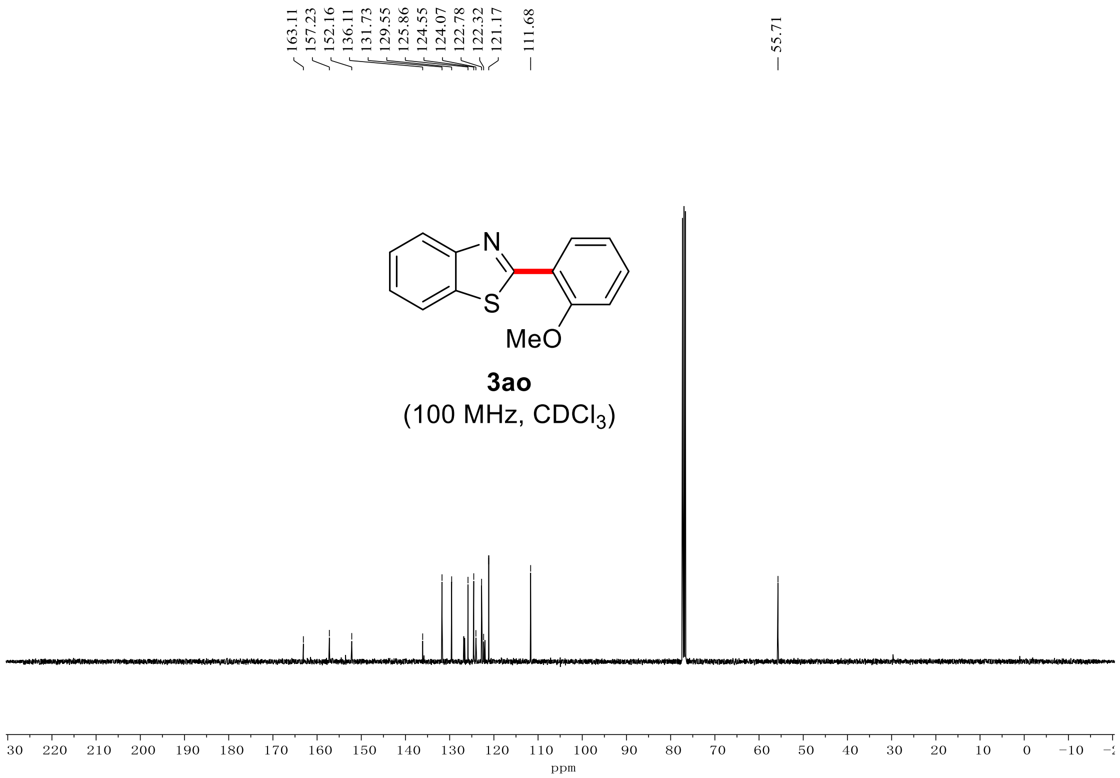
163.11
157.23
152.16
136.11
131.73
129.55
125.86
124.55
124.07
122.78
122.32
121.17
111.68
55.71
N
S
MeO
3ao
(100 MHz, CDCl3)
ppm

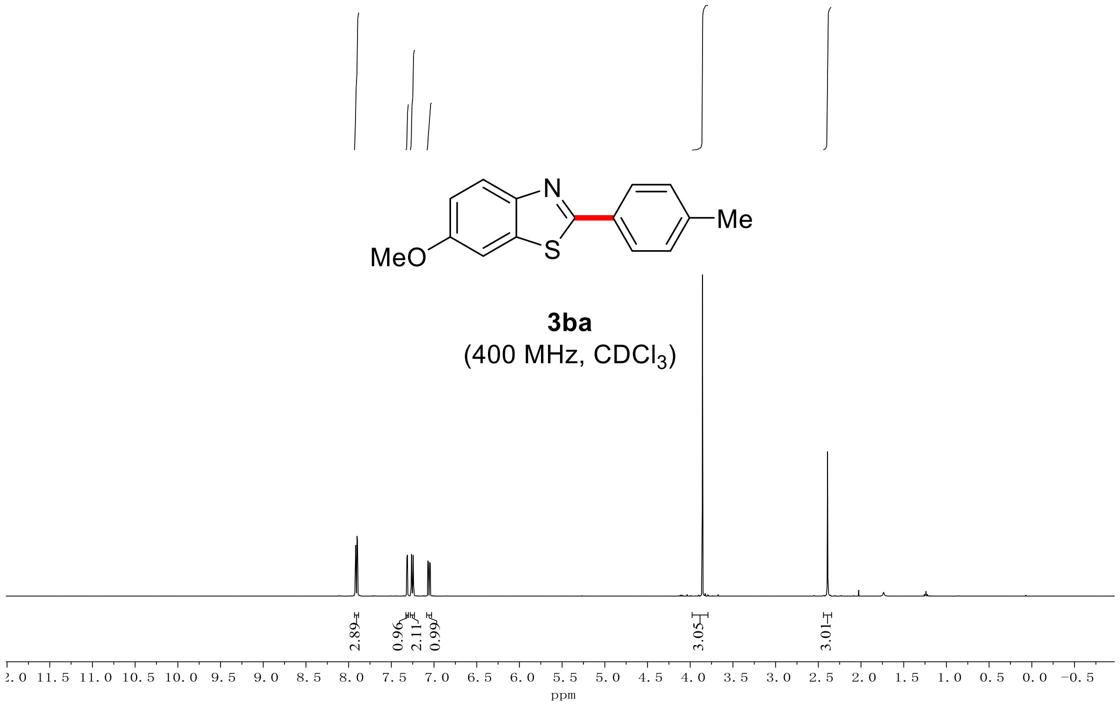

N
S
MeO
Me
3ba
(400 MHz, CDCl3)
2.89
0.96
2.11
0.99
3.05
3.01
ppm

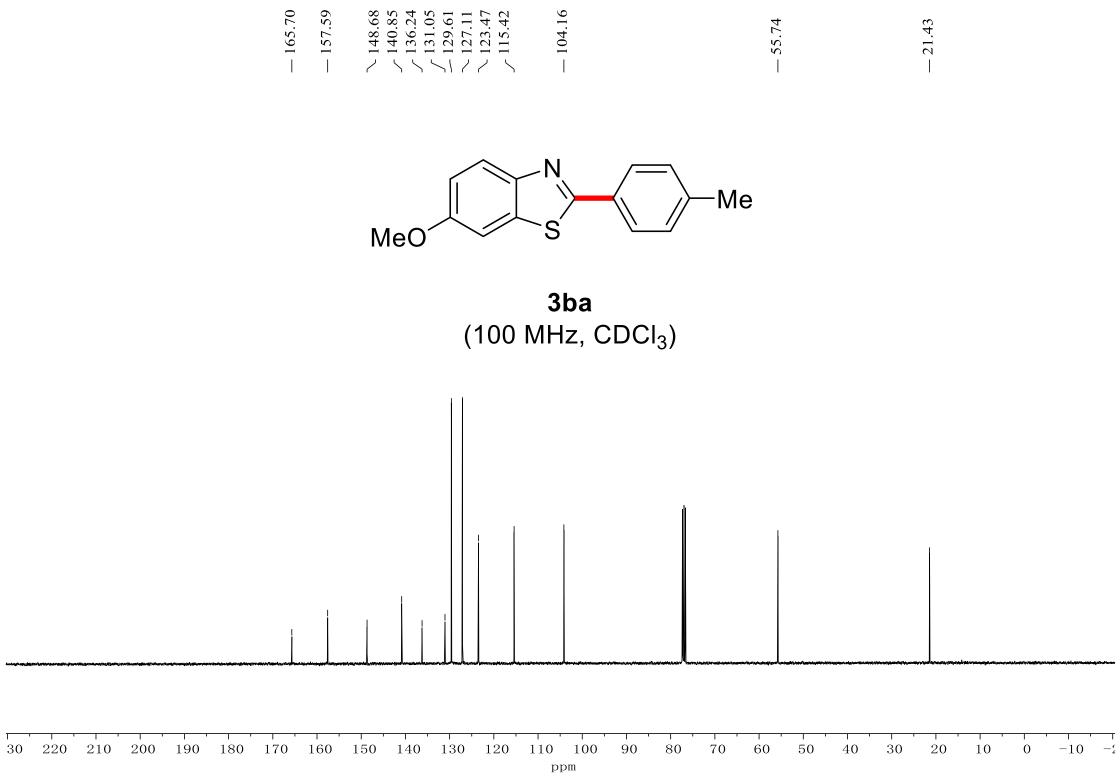

165.70
157.59
148.68
140.85
136.24
131.05
129.61
127.11
123.47
115.42
104.16
55.74
21.43
N
S
MeO
Me
3ba
(100 MHz, CDCl3)
ppm

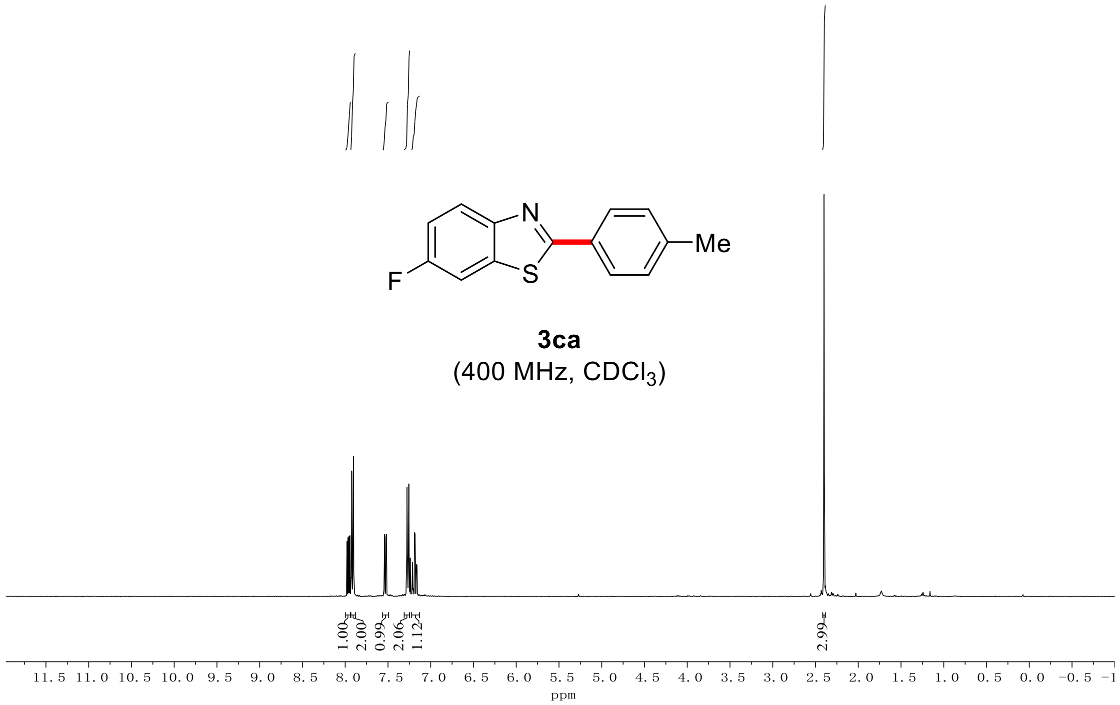
3ca
(400 MHz, CDCl$_3$)
Me
N
S
F
1.00
2.00
0.99
2.06
1.12
2.99
ppm

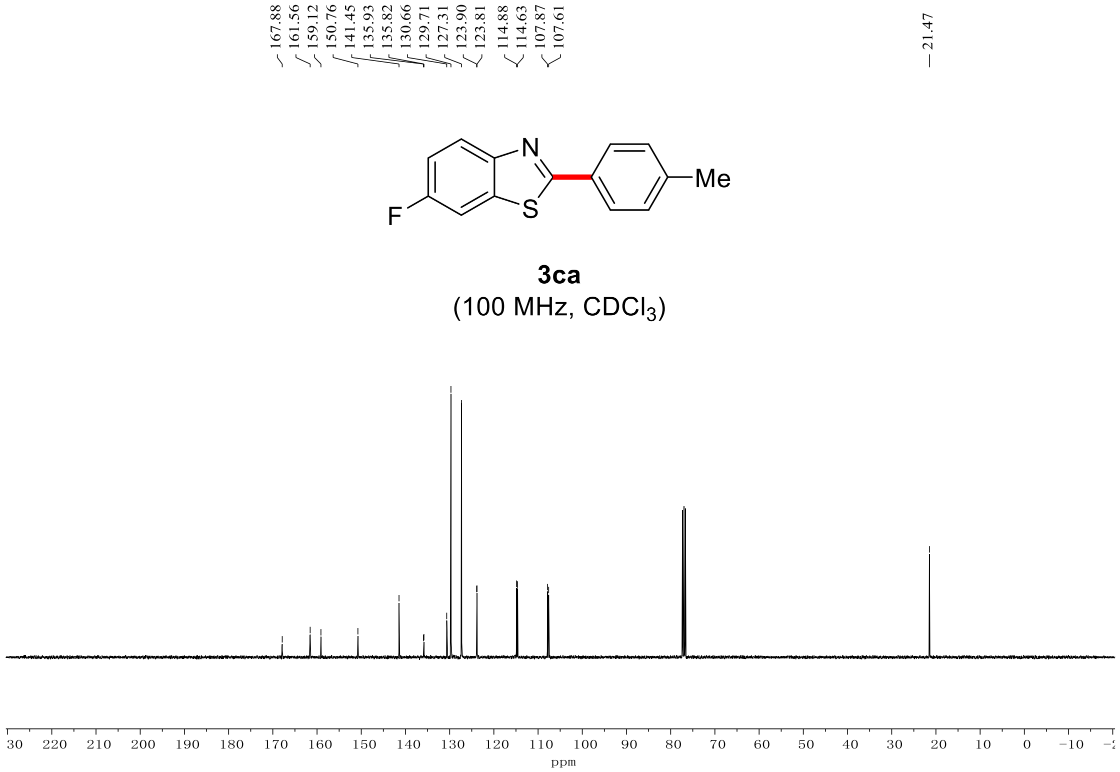
167.88
161.56
159.12
150.76
141.45
135.93
135.82
130.66
129.71
127.31
123.90
123.81
114.88
114.63
107.87
107.61
21.47
3ca
(100 MHz, CDCl$_3$)
Me
N
S
F
ppm

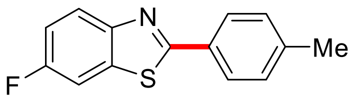

3ca
(282 MHz, $CDCl_3$)

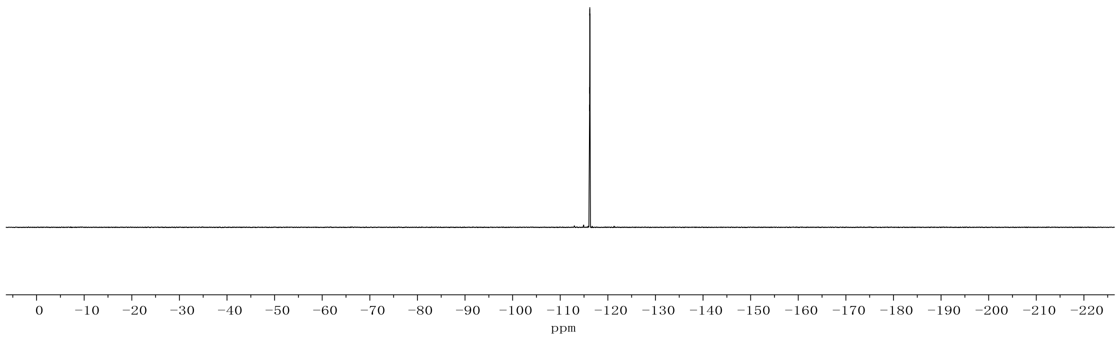

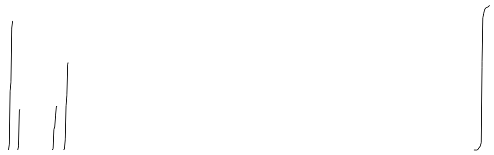

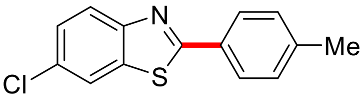

3da
(400 MHz, $CDCl_3$)

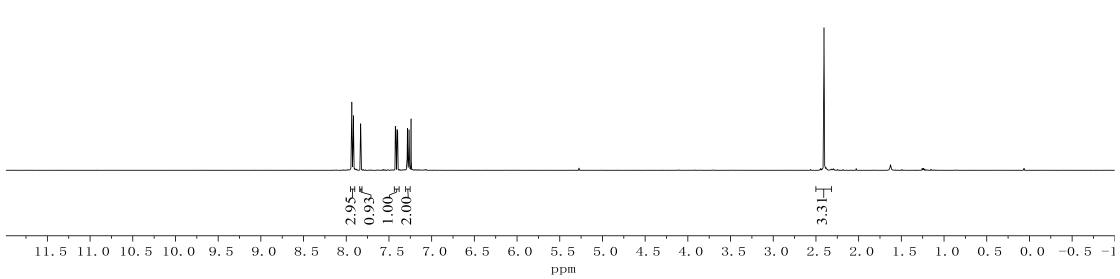

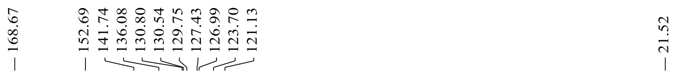

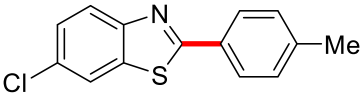

3da
(100 MHz, $CDCl_3$)

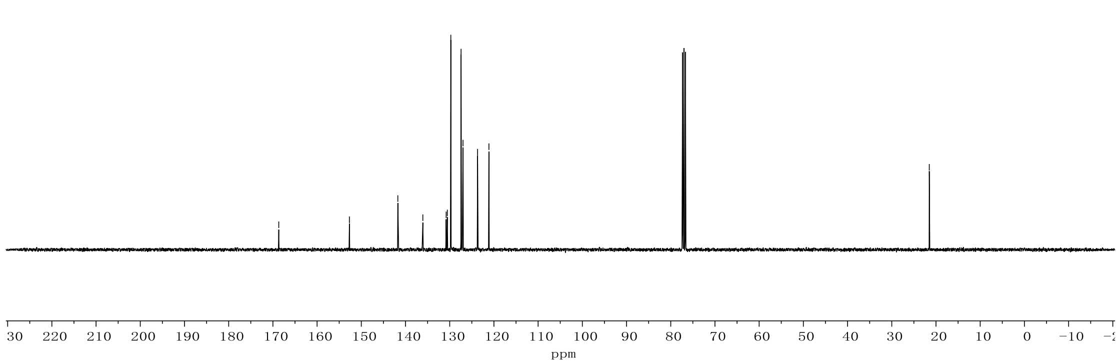

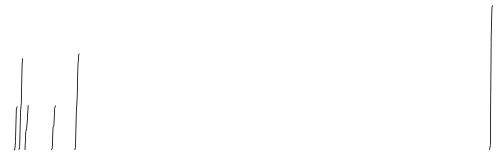

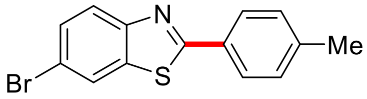

3ea
(400 MHz, $CDCl_3$)

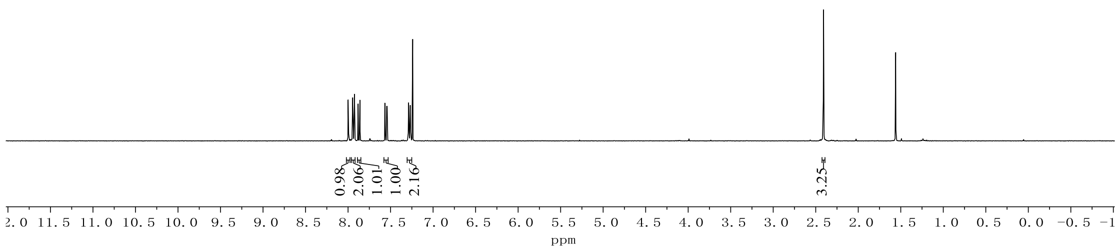

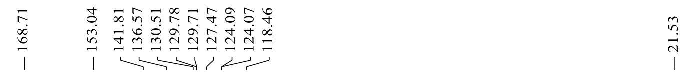

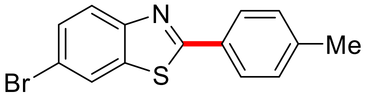

3ea
(100 MHz, $CDCl_3$)

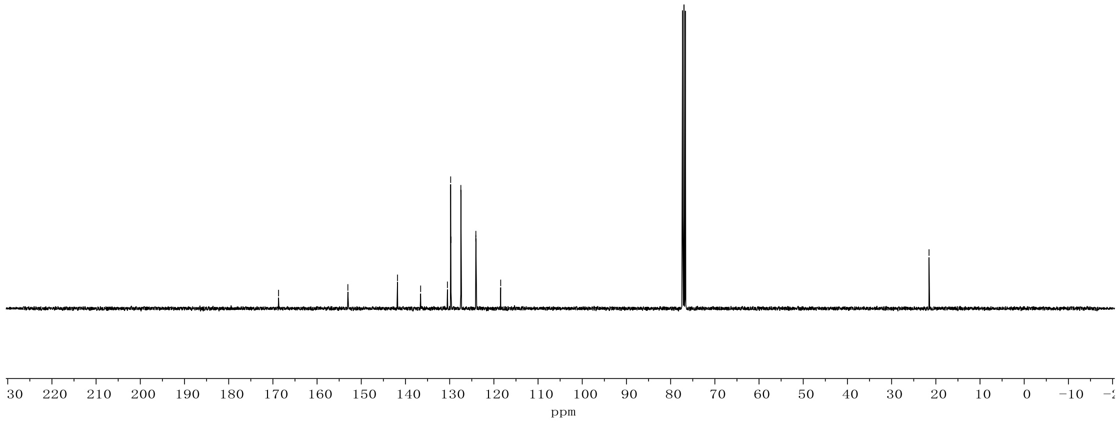

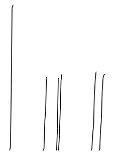

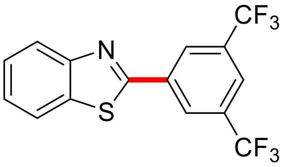

3ap
(500 MHz, $CDCl_3$)

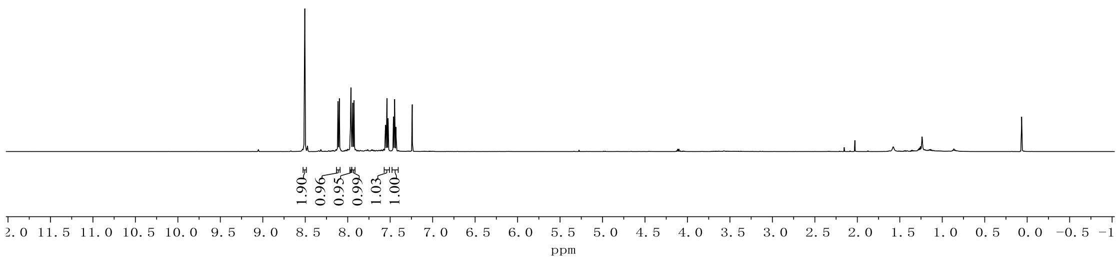

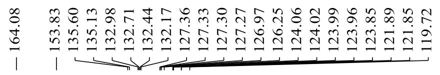

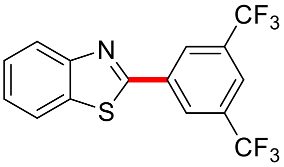

3ap
(125 MHz, $CDCl_3$)

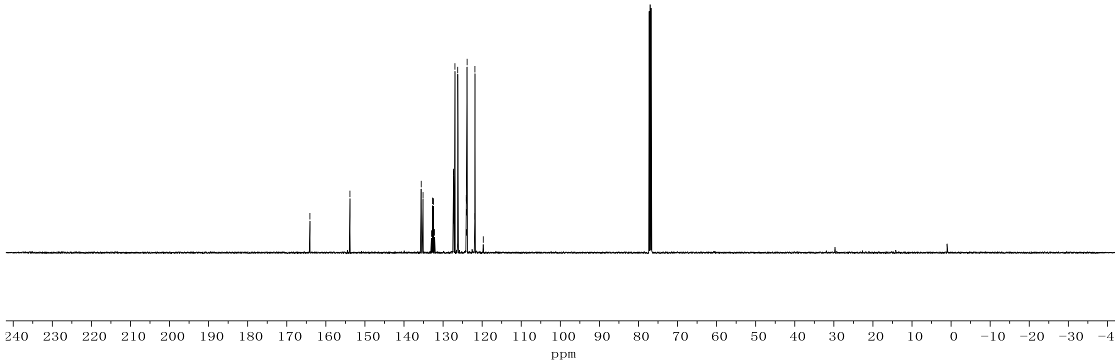

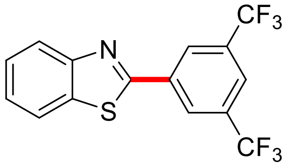

3ap

(282 MHz, $CDCl_3$)

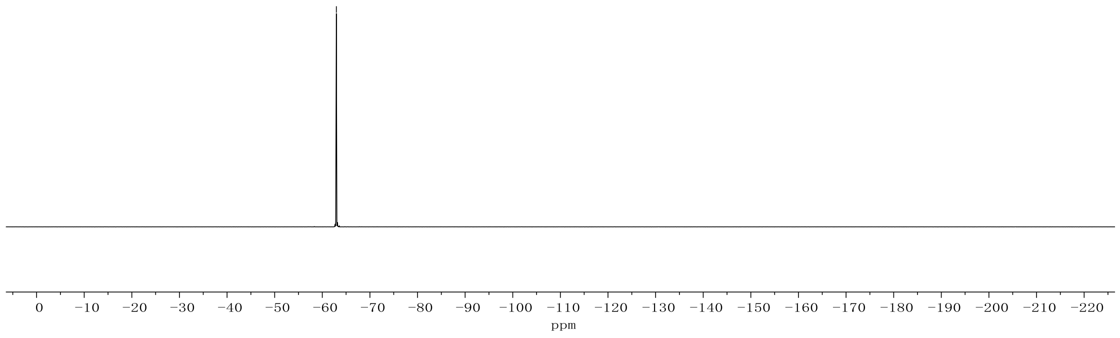

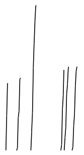

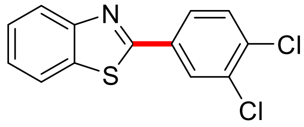

3aq
(500 MHz, $CDCl_3$)

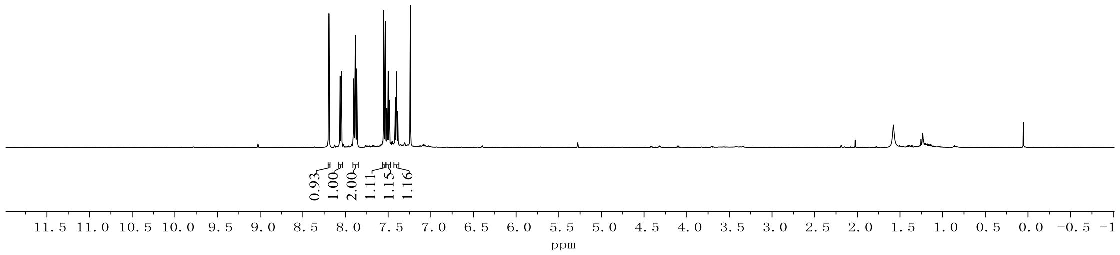

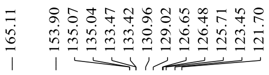

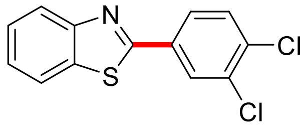

3aq
(125 MHz, $CDCl_3$)

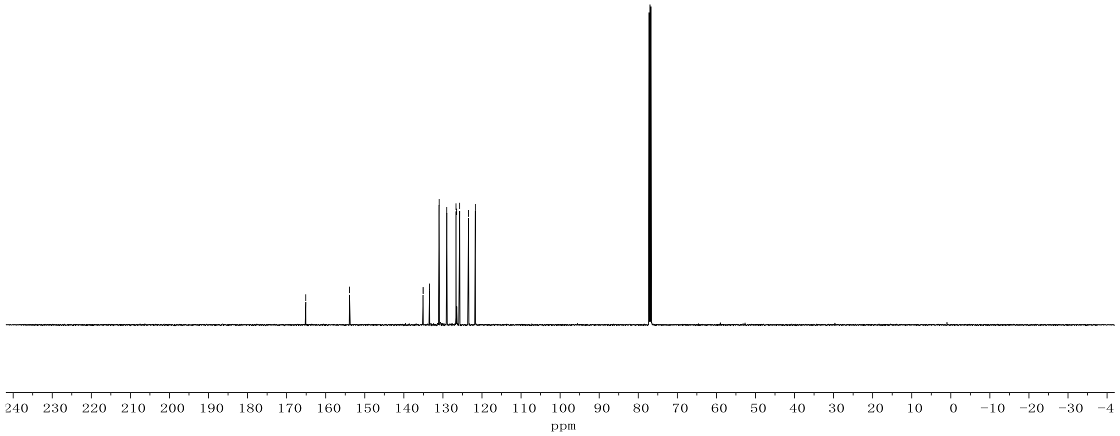

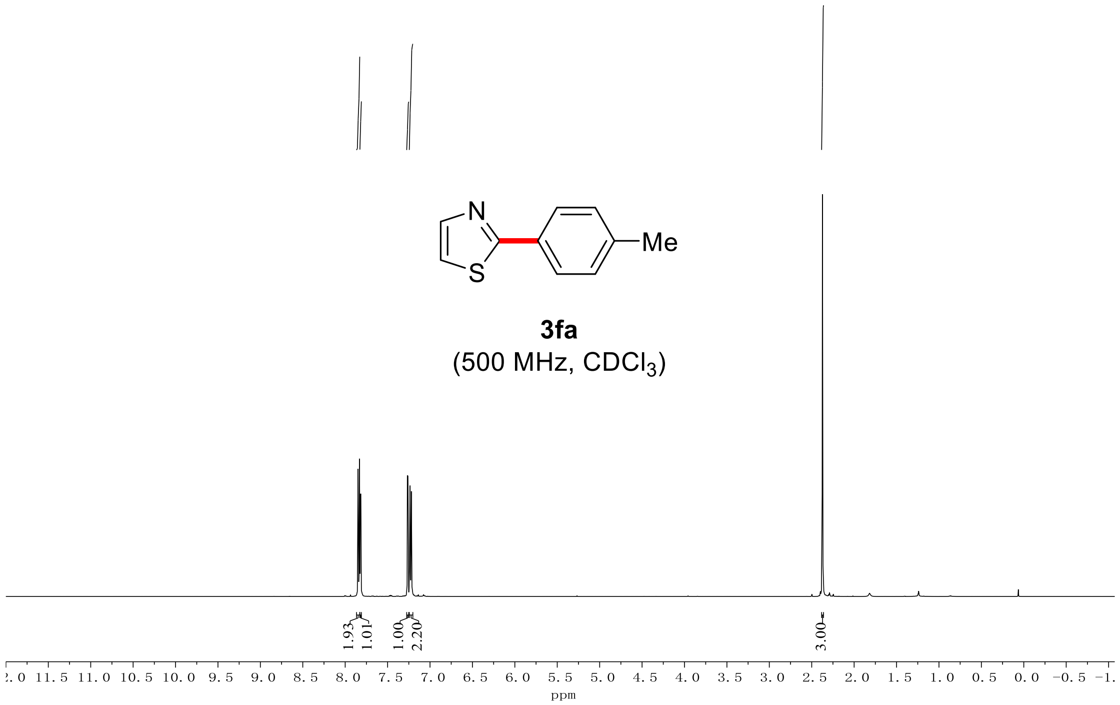
Me
N
S
3fa
(500 MHz, CDCl$_3$)
1.93
1.01
1.00
2.20
3.00
ppm

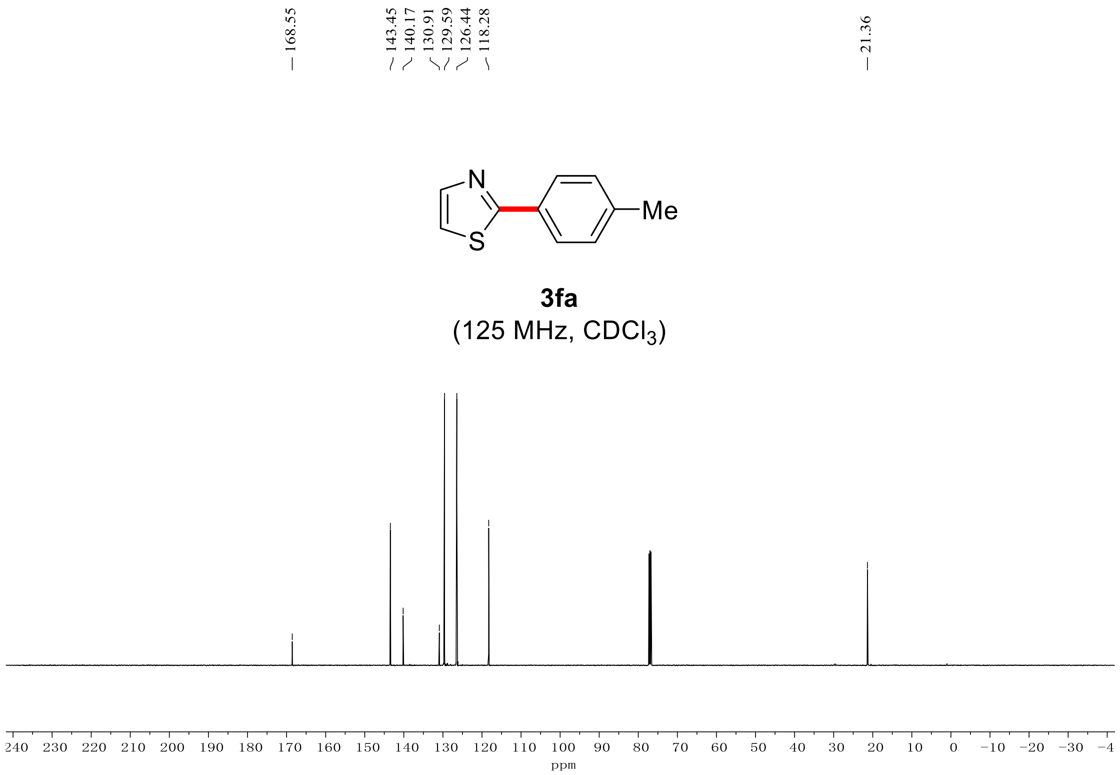
168.55
143.45
140.17
130.91
129.59
126.44
118.28
21.36
Me
N
S
3fa
(125 MHz, CDCl$_3$)
ppm

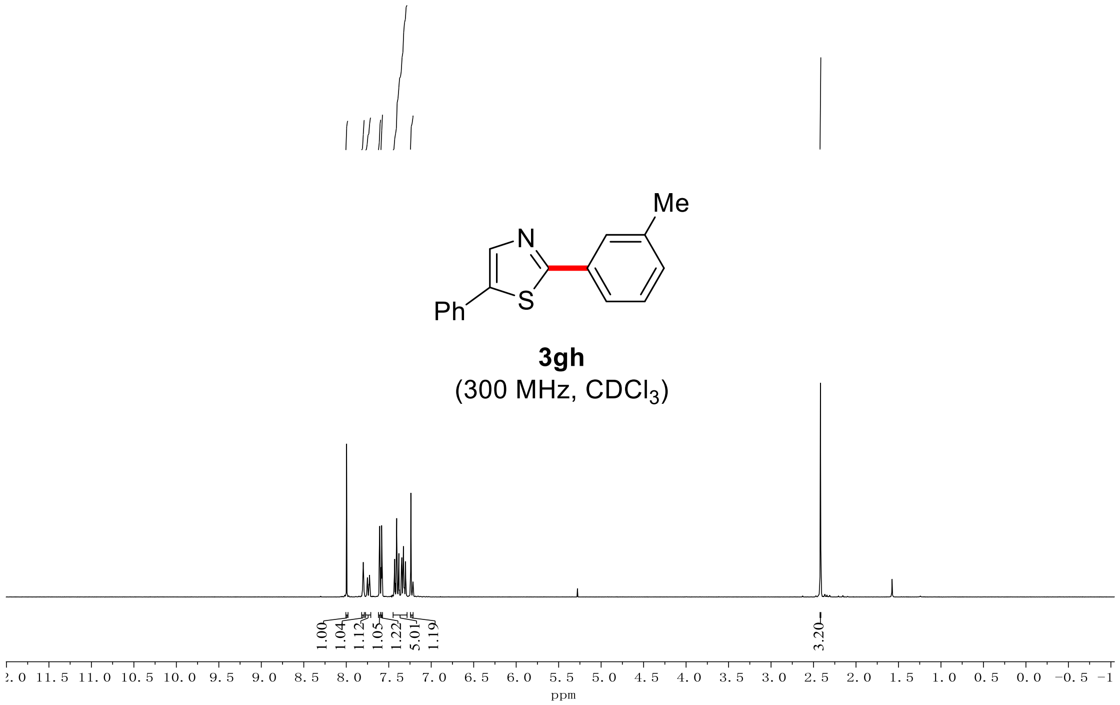
Me
N
S
Ph
3gh
(300 MHz, CDCl3)
1.00
1.04
1.12
1.05
1.22
5.01
1.19
3.20
ppm

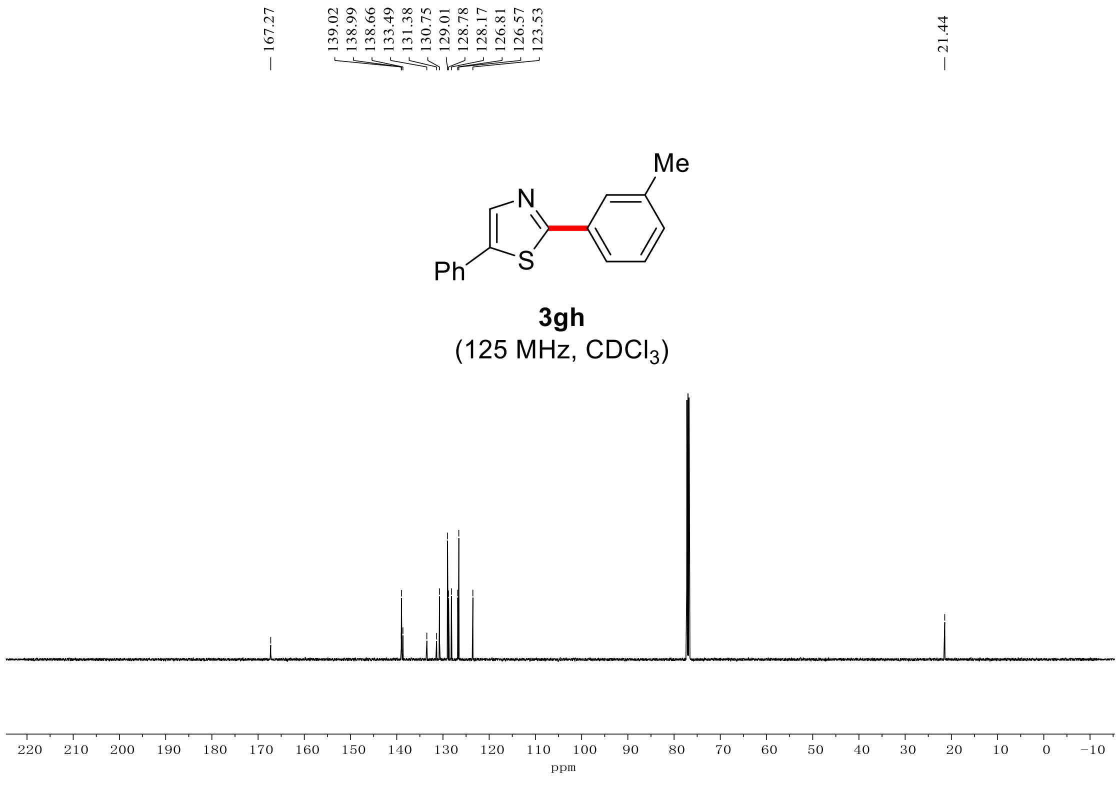
167.27
139.02
138.99
138.66
133.49
131.38
130.75
129.01
128.78
128.17
126.81
126.57
123.53
21.44
Me
N
S
Ph
3gh
(125 MHz, CDCl3)
ppm

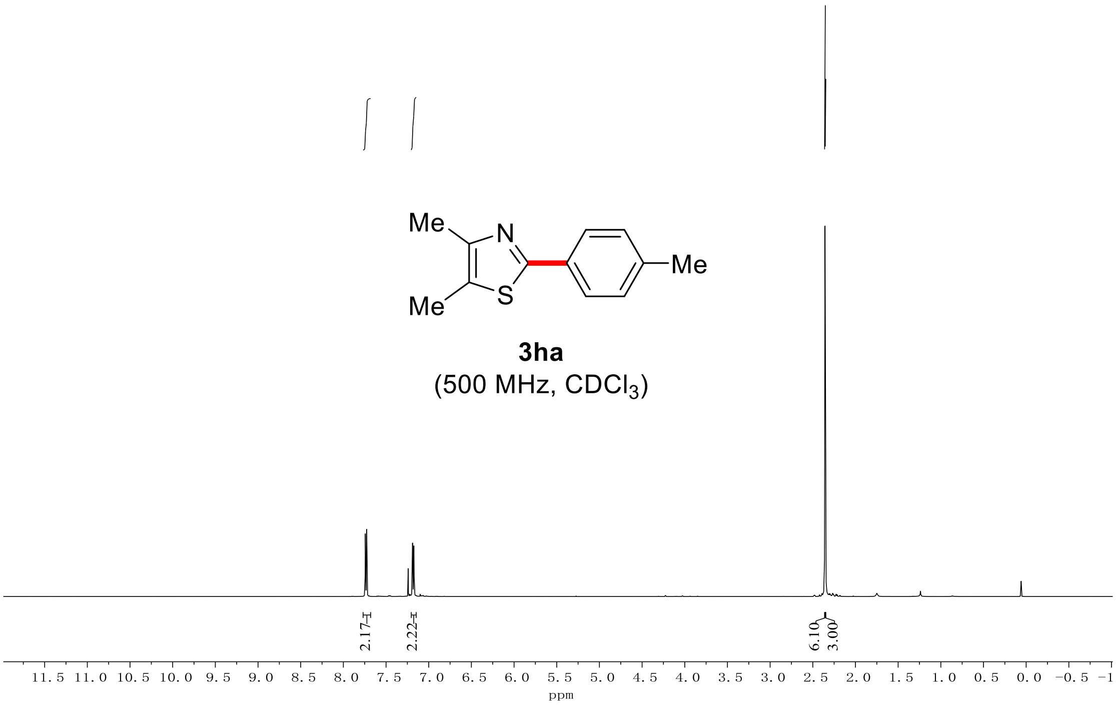
Me
N
Me
S
Me
3ha
(500 MHz, CDCl$_3$)
2.17
2.22
6.10
3.00
ppm

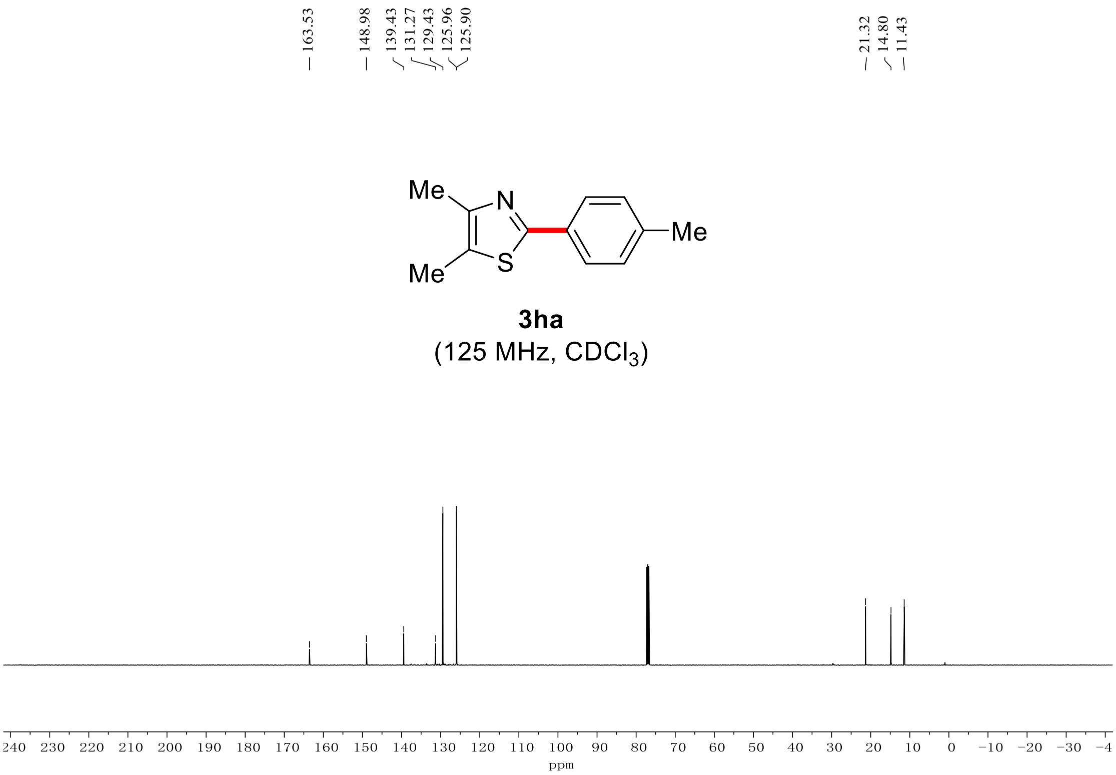
163.53
148.98
139.43
131.27
129.43
125.96
125.90
21.32
14.80
11.43
Me
N
Me
S
Me
3ha
(125 MHz, CDCl$_3$)
ppm

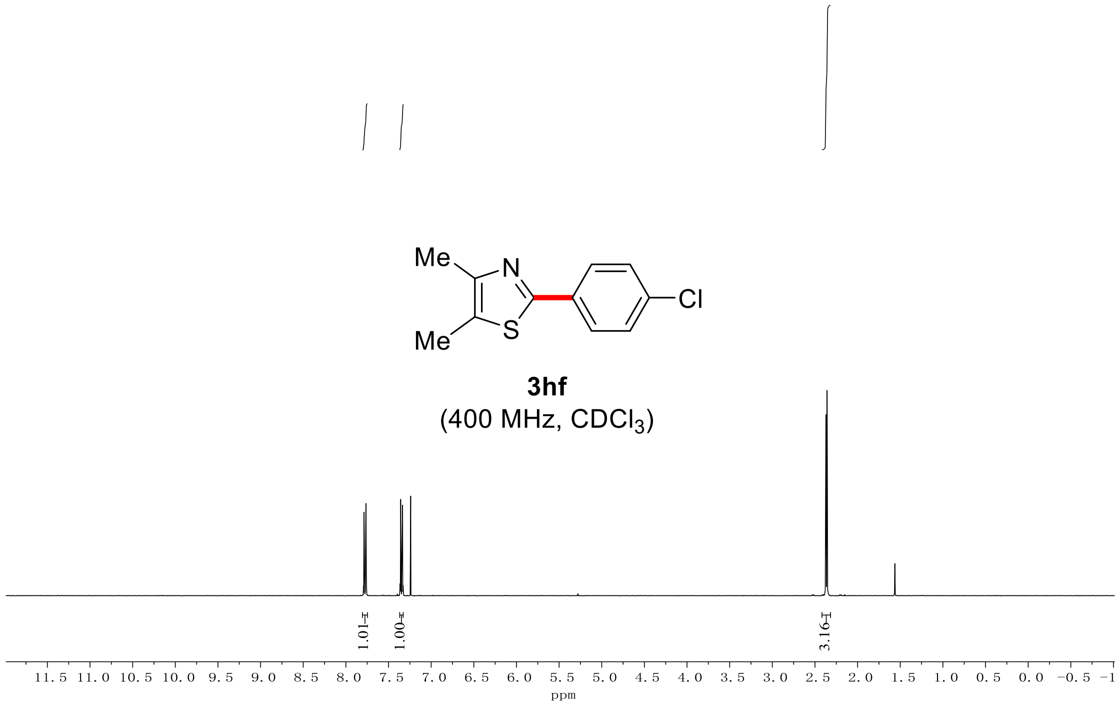
Me
N
S
Me
Cl
3hf
(400 MHz, CDCl3)
1.01
1.00
3.16
11.5 11.0 10.5 10.0 9.5 9.0 8.5 8.0 7.5 7.0 6.5 6.0 5.5 5.0 4.5 4.0 3.5 3.0 2.5 2.0 1.5 1.0 0.5 0.0 -0.5 -1
ppm

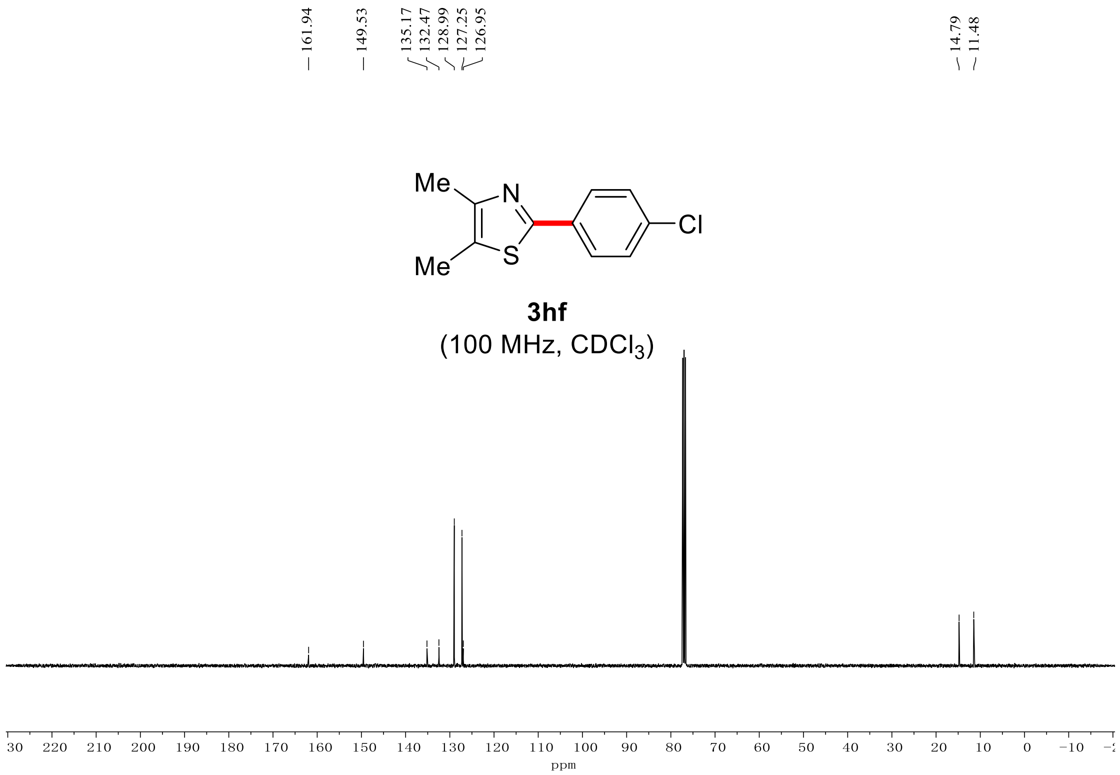
161.94
149.53
135.17
132.47
128.99
127.25
126.95
14.79
11.48
Me
N
S
Me
Cl
3hf
(100 MHz, CDCl3)
30 220 210 200 190 180 170 160 150 140 130 120 110 100 90 80 70 60 50 40 30 20 10 0 -10
ppm

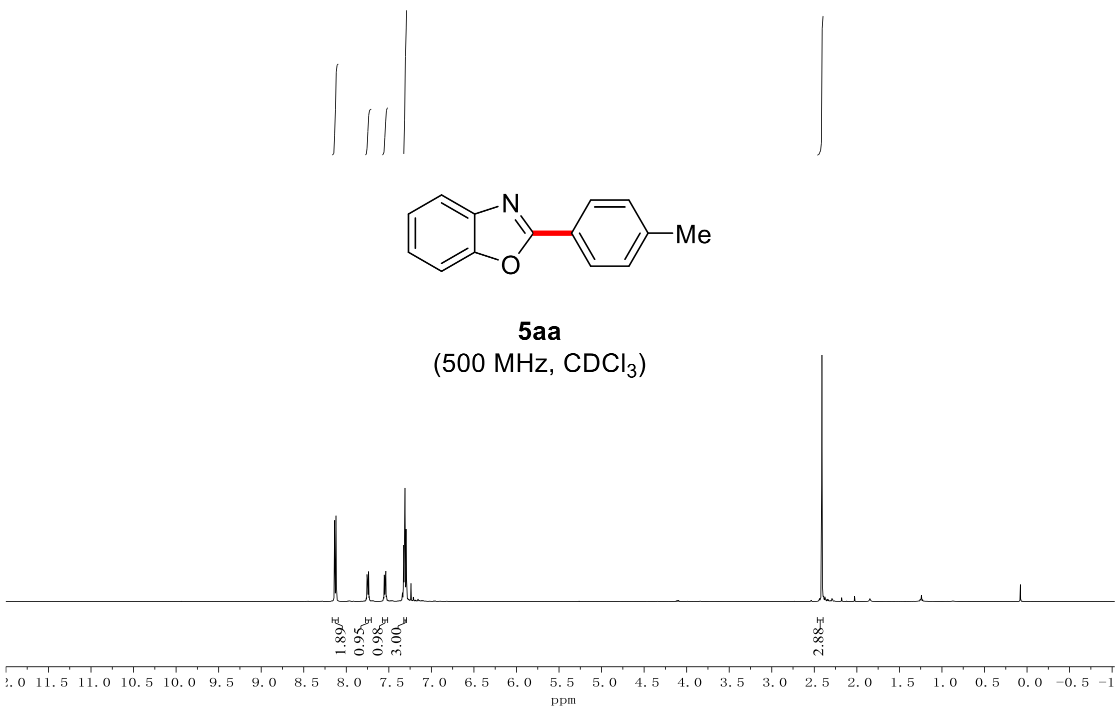

5aa
(500 MHz, $CDCl_3$)

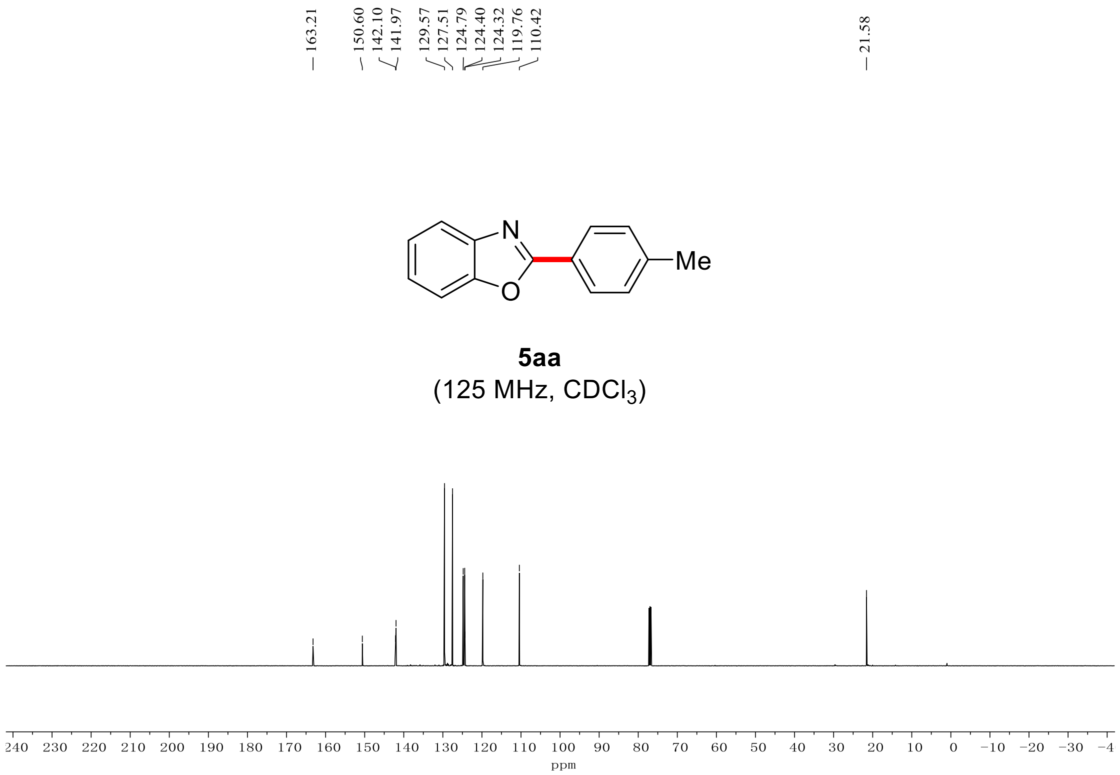

5aa
(125 MHz, $CDCl_3$)

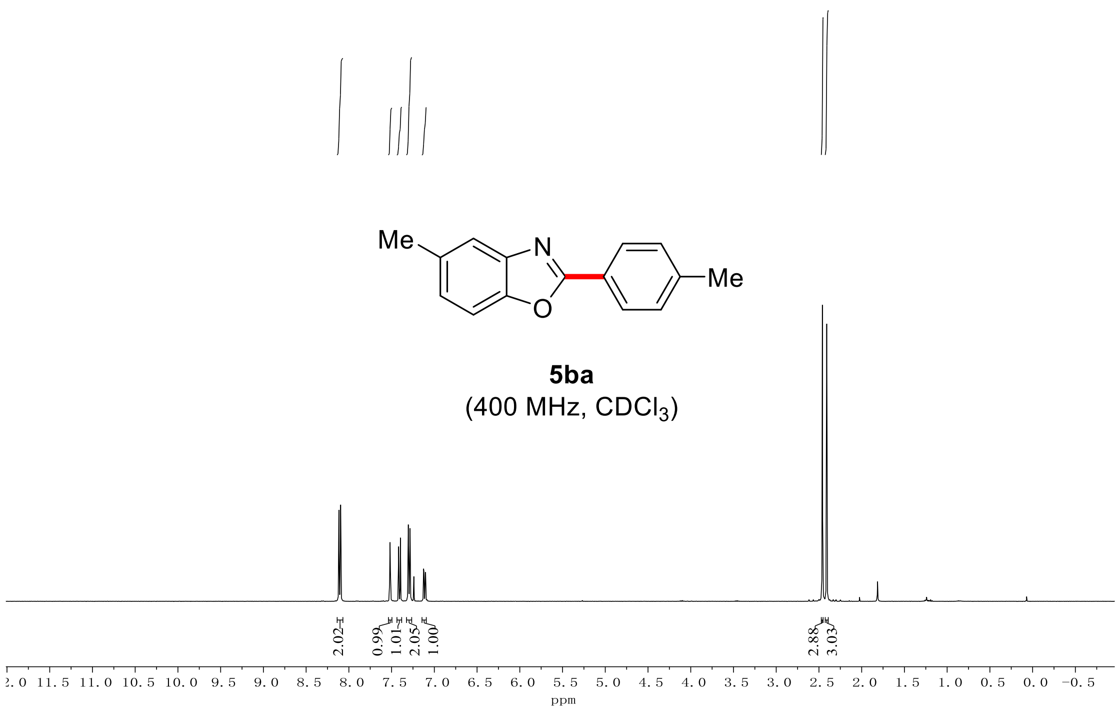
Me
N
O
Me
5ba
(400 MHz, CDCl3)
2.02
0.99
1.01
2.05
1.00
2.88
3.03
ppm

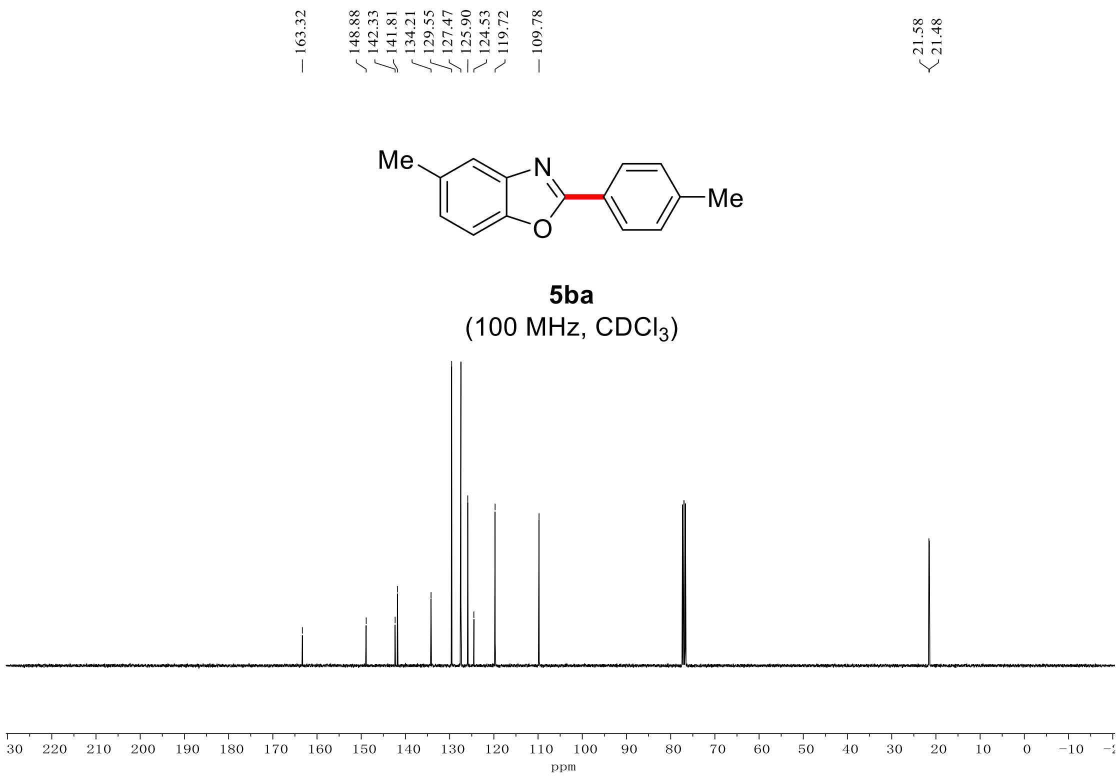
163.32
148.88
142.33
141.81
134.21
129.55
127.47
125.90
124.53
119.72
109.78
21.58
21.48
Me
N
O
Me
5ba
(100 MHz, CDCl3)
ppm

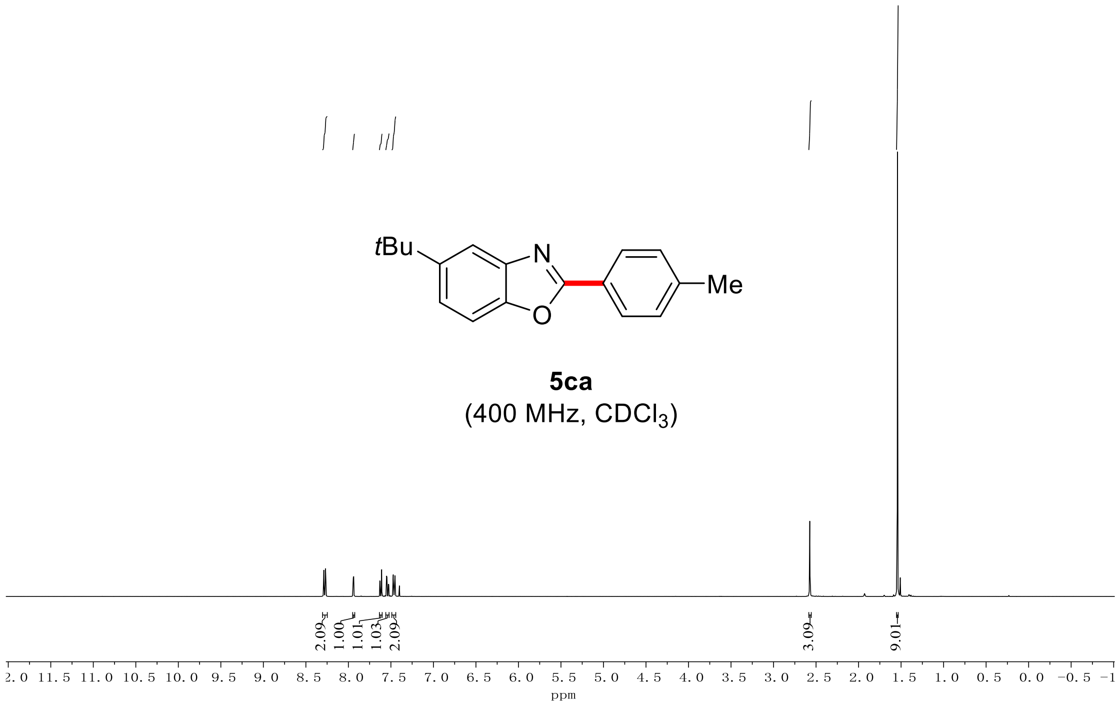
tBu
N
O
Me
5ca
(400 MHz, CDCl3)
2.09
1.00
1.01
1.03
2.09
3.09
9.01
ppm

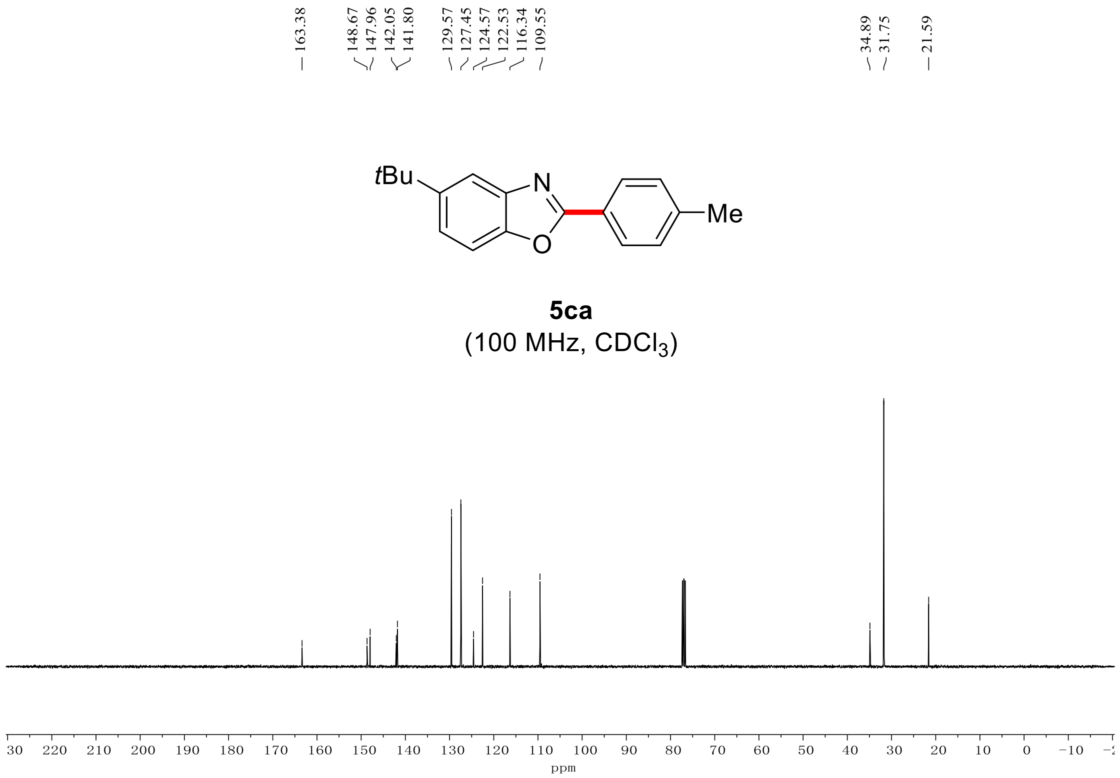
163.38
148.67
147.96
142.05
141.80
129.57
127.45
124.57
122.53
116.34
109.55
34.89
31.75
21.59
tBu
N
O
Me
5ca
(100 MHz, CDCl3)
ppm

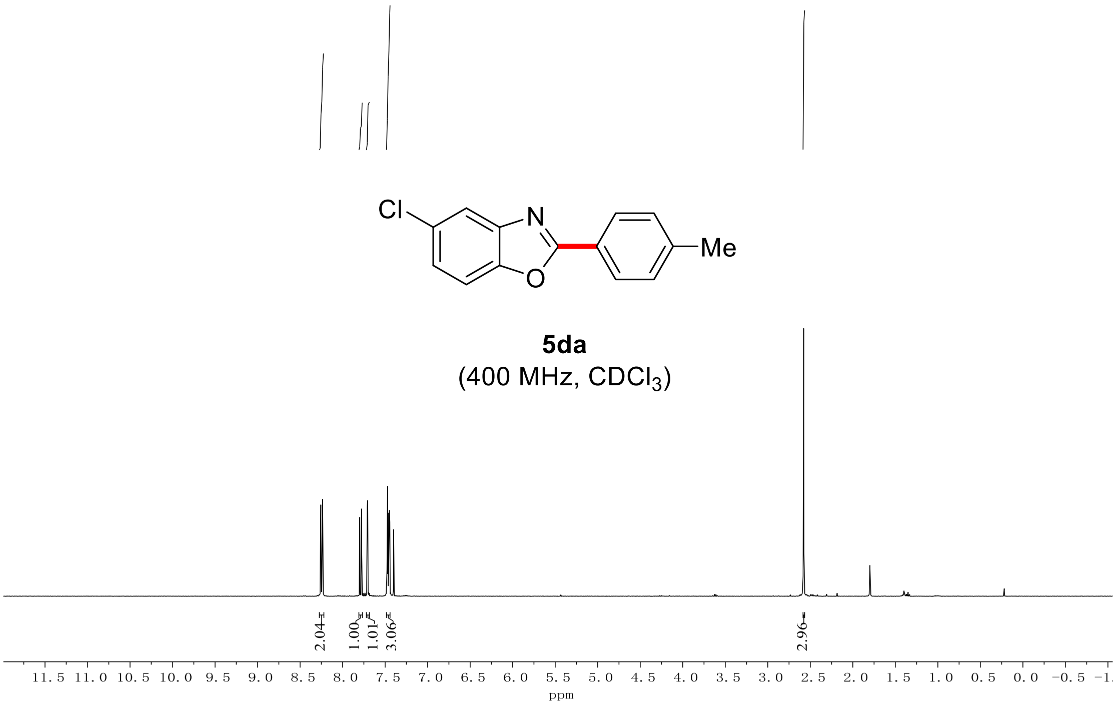

5da
(400 MHz, $CDCl_3$)

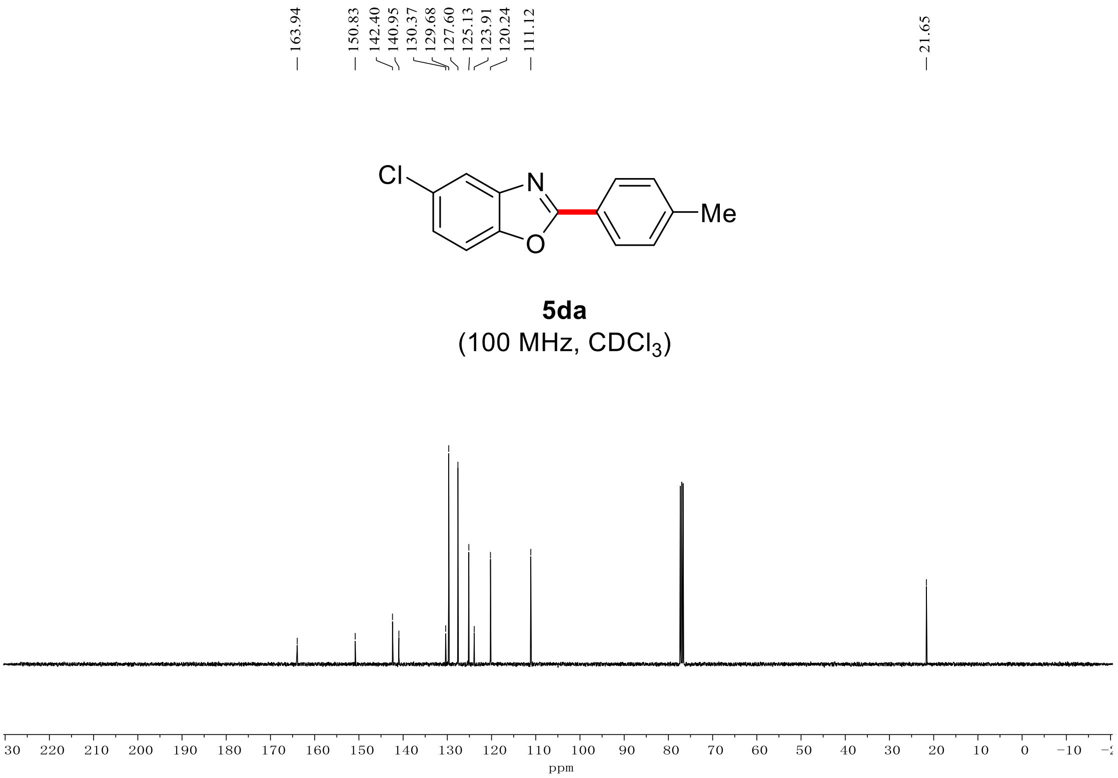

5da
(100 MHz, $CDCl_3$)

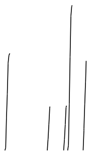

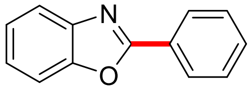

5ab
(400 MHz, $CDCl_3$)

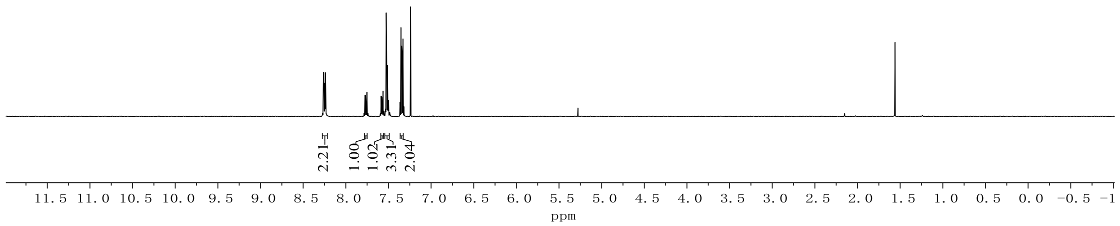

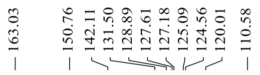

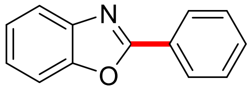

5ab
(100 MHz, $CDCl_3$)

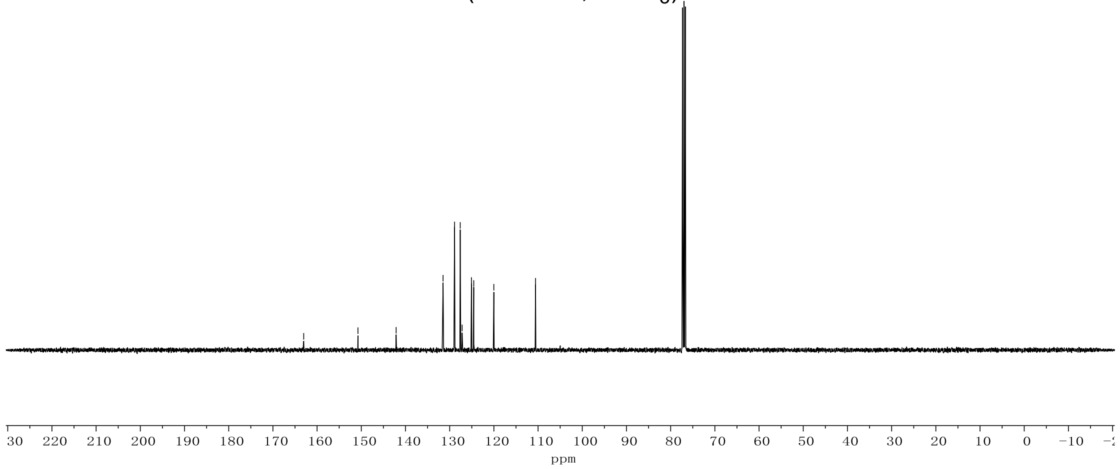

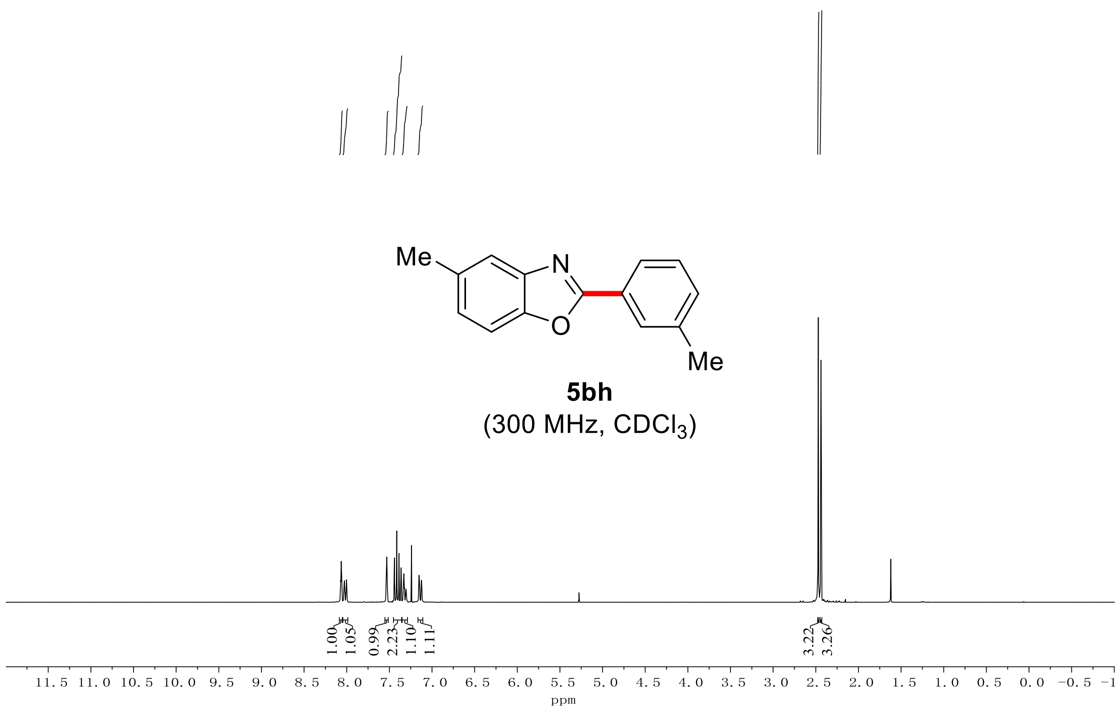
Me
N
O
Me
5bh
(300 MHz, $CDCl_3$)
1.00
1.05
0.99
2.23
1.10
1.11
3.22
3.26
11.5 11.0 10.5 10.0 9.5 9.0 8.5 8.0 7.5 7.0 6.5 6.0 5.5 5.0 4.5 4.0 3.5 3.0 2.5 2.0 1.5 1.0 0.5 0.0 −0.5 −1
ppm

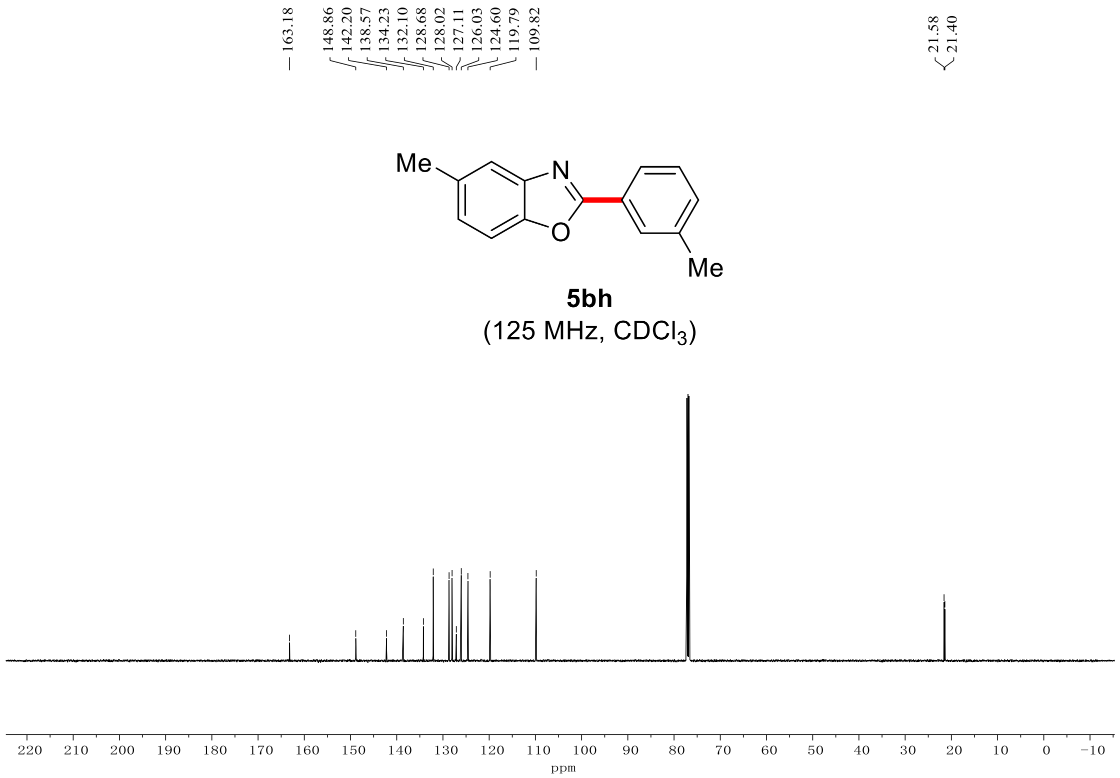
163.18
148.86
142.20
138.57
134.23
132.10
128.68
128.02
127.11
126.03
124.60
119.79
109.82
21.58
21.40
Me
N
O
Me
5bh
(125 MHz, $CDCl_3$)
220 210 200 190 180 170 160 150 140 130 120 110 100 90 80 70 60 50 40 30 20 10 0 −10
ppm

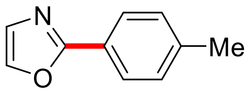

5ea
(400 MHz, $CDCl_3$)

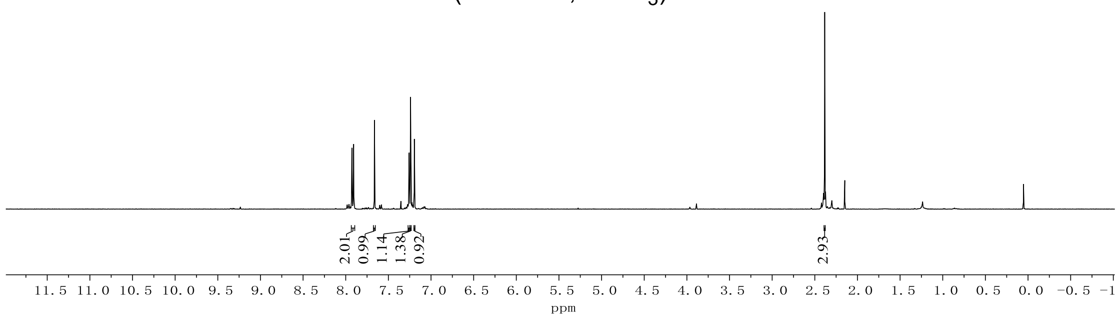

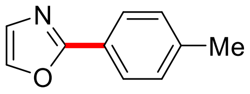

5ea
(100 MHz, $CDCl_3$)

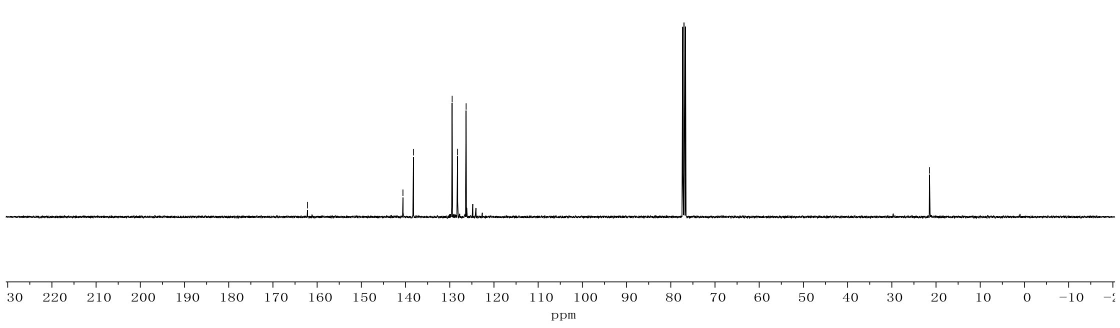

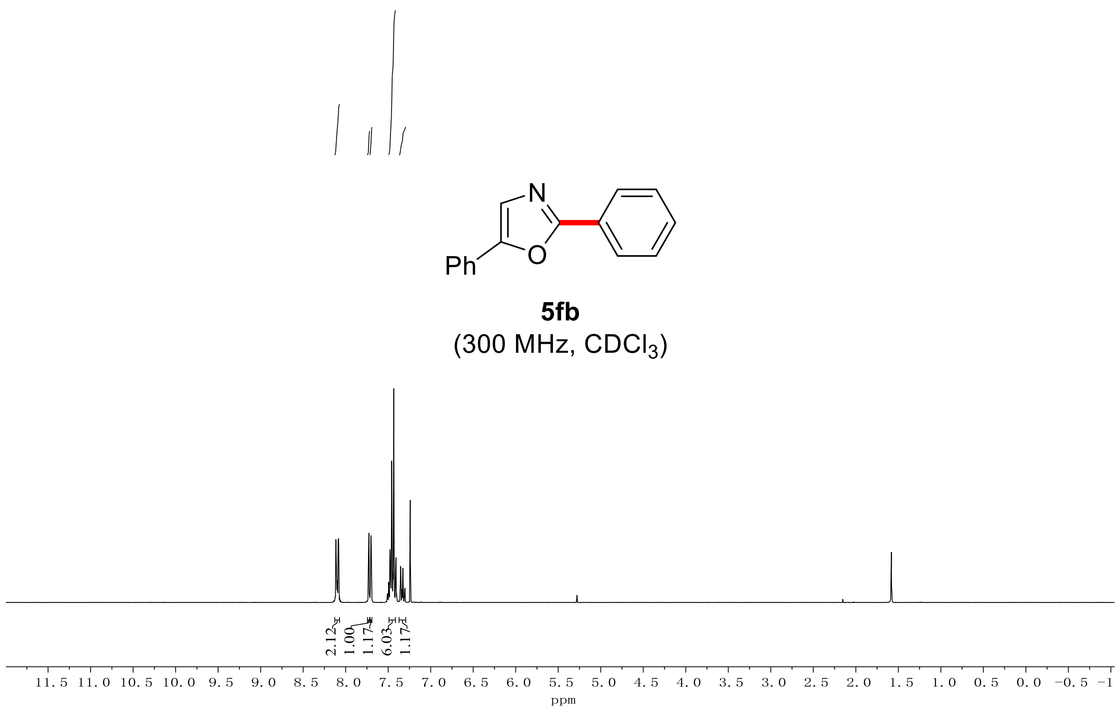
N
O
Ph
5fb
(300 MHz, CDCl$_3$)
2.12
1.00
1.17
6.03
1.17
11.5 11.0 10.5 10.0 9.5 9.0 8.5 8.0 7.5 7.0 6.5 6.0 5.5 5.0 4.5 4.0 3.5 3.0 2.5 2.0 1.5 1.0 0.5 0.0 −0.5 −1
ppm

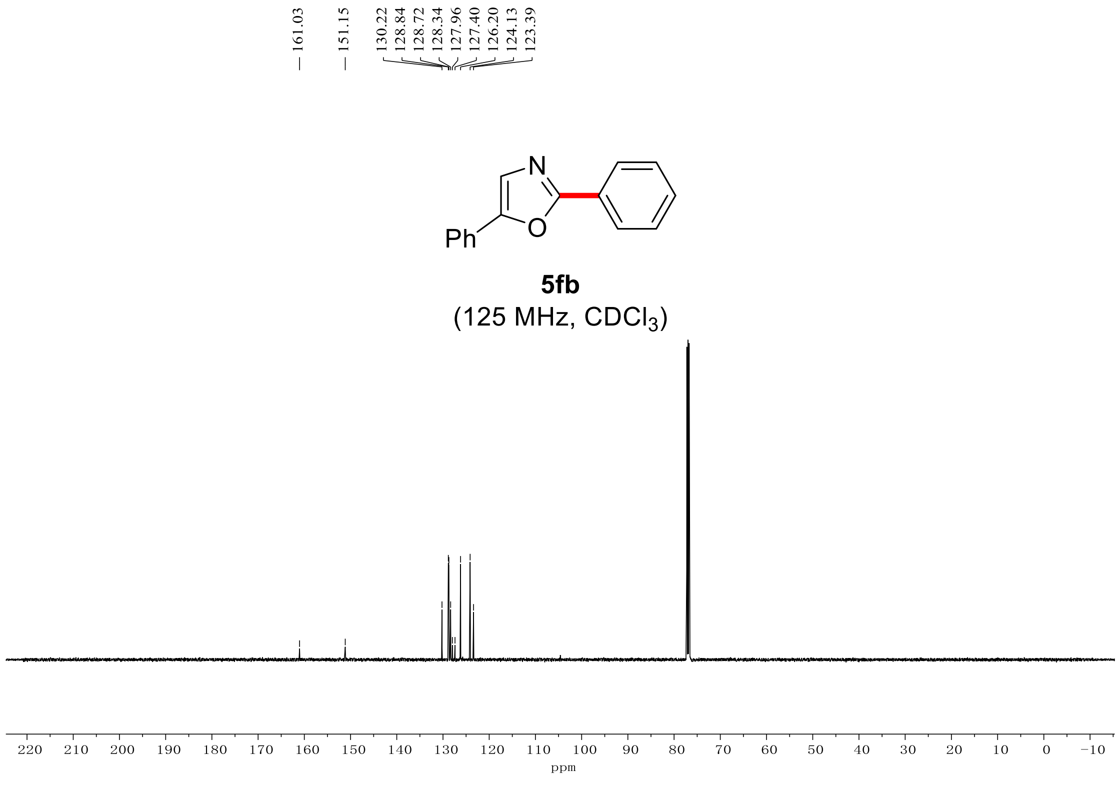
161.03
151.15
130.22
128.84
128.72
128.34
127.96
127.40
126.20
124.13
123.39
N
O
Ph
5fb
(125 MHz, CDCl$_3$)
220 210 200 190 180 170 160 150 140 130 120 110 100 90 80 70 60 50 40 30 20 10 0 −10
ppm

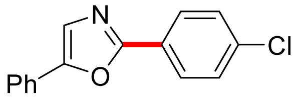

5ff
(400 MHz, $CDCl_3$)

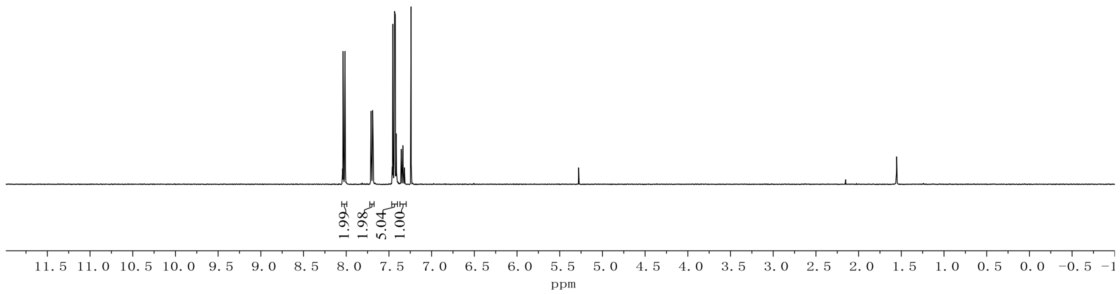

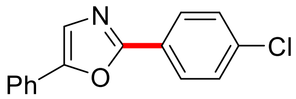

5ff
(100 MHz, $CDCl_3$)

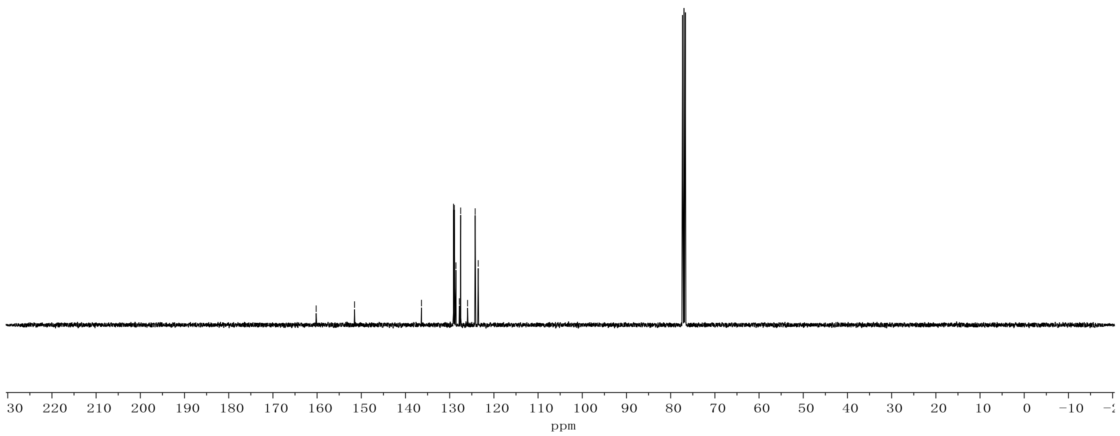

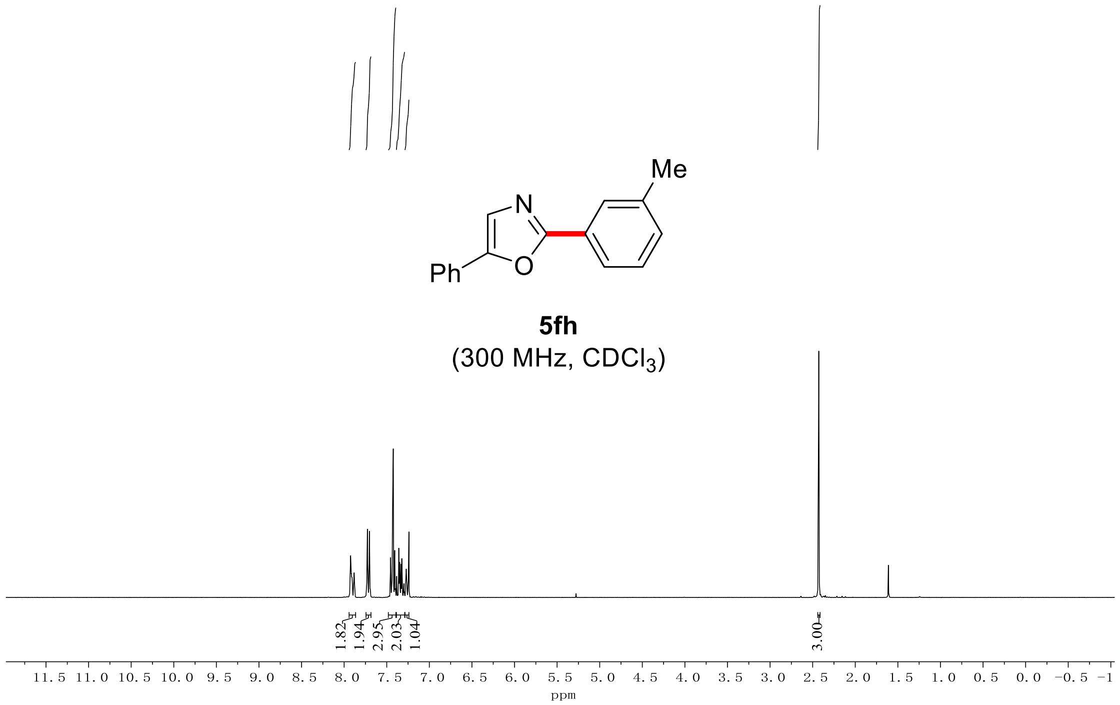

5fh
(300 MHz, $CDCl_3$)

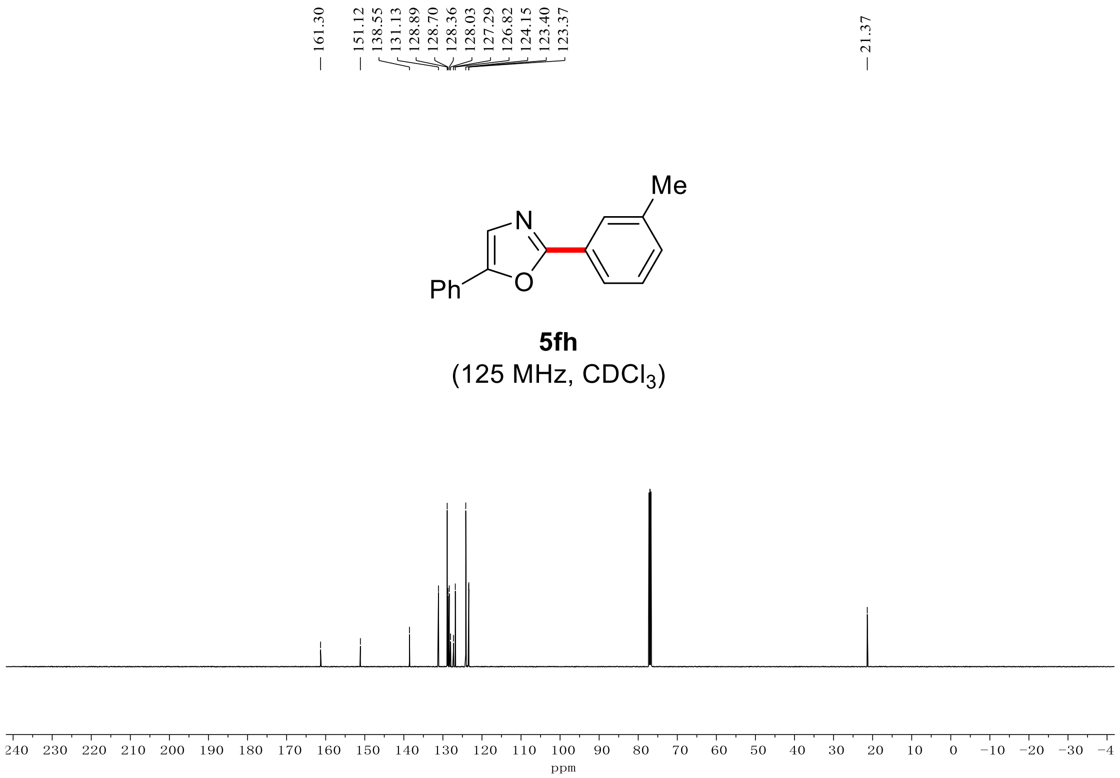

5fh
(125 MHz, $CDCl_3$)

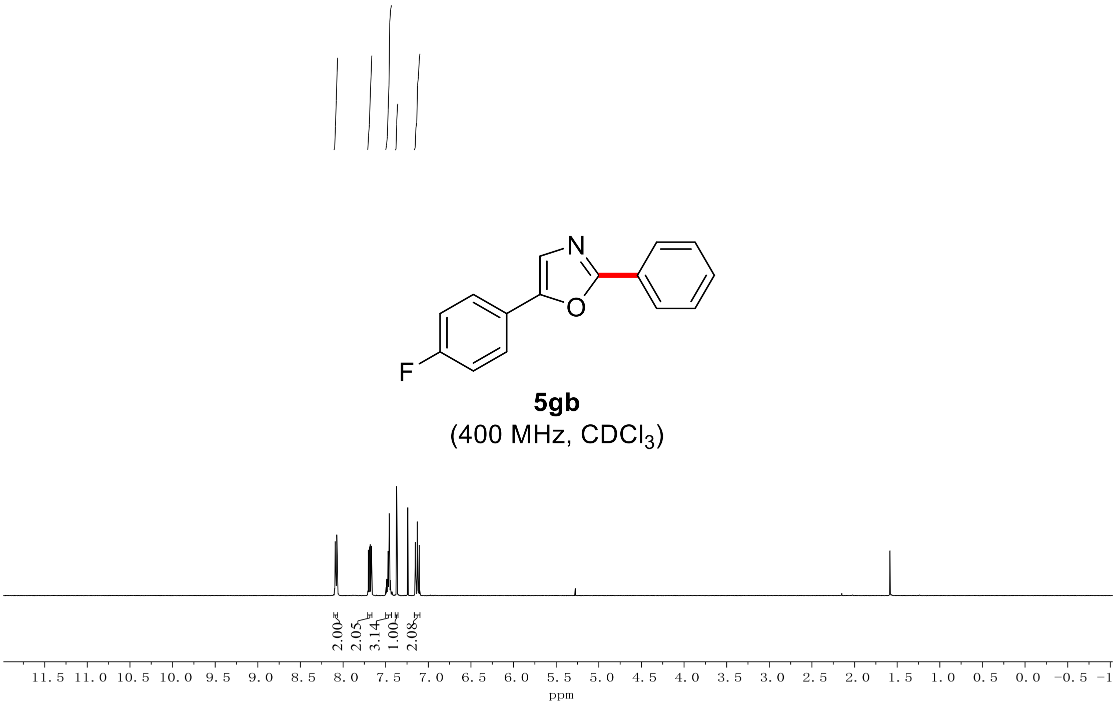
5gb
(400 MHz, CDCl3)
2.00
2.05
3.14
1.00
2.08
11.5 11.0 10.5 10.0 9.5 9.0 8.5 8.0 7.5 7.0 6.5 6.0 5.5 5.0 4.5 4.0 3.5 3.0 2.5 2.0 1.5 1.0 0.5 0.0 −0.5 −1
ppm

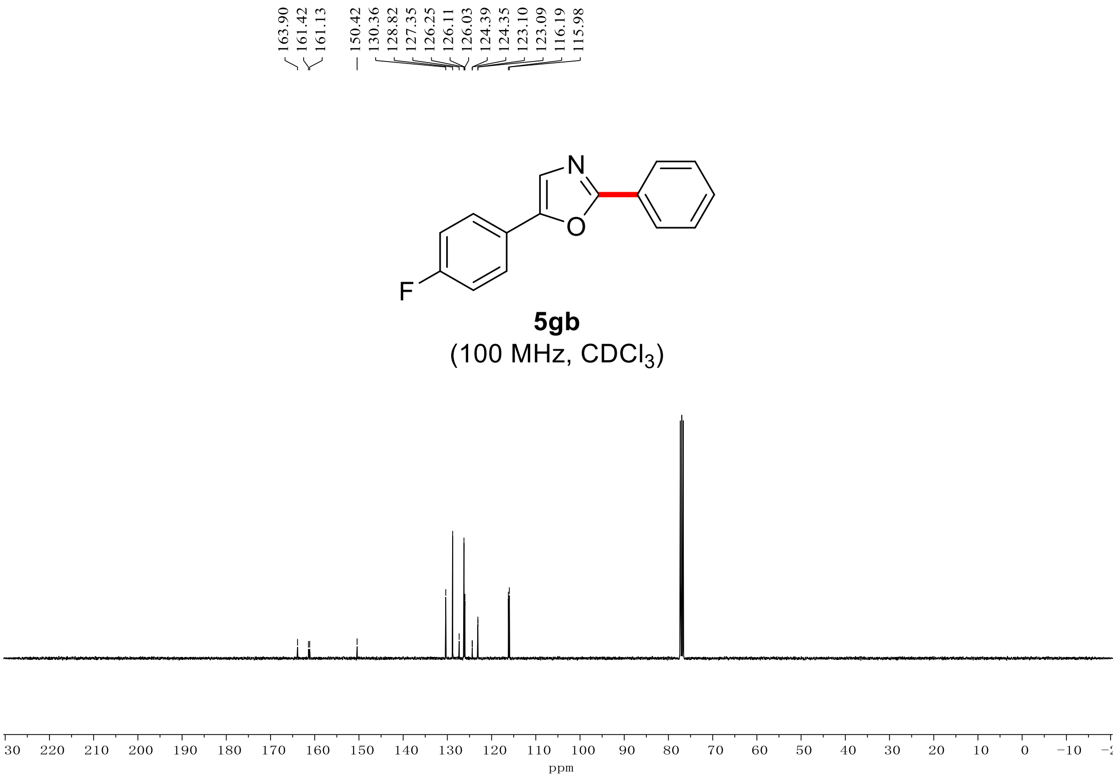
163.90
161.42
161.13
150.42
130.36
128.82
127.35
126.25
126.11
126.03
124.39
124.35
123.10
123.09
116.19
115.98
5gb
(100 MHz, CDCl3)
30 220 210 200 190 180 170 160 150 140 130 120 110 100 90 80 70 60 50 40 30 20 10 0 −10
ppm

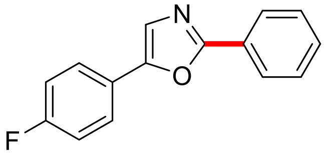

5gb
(282 MHz, $CDCl_3$)

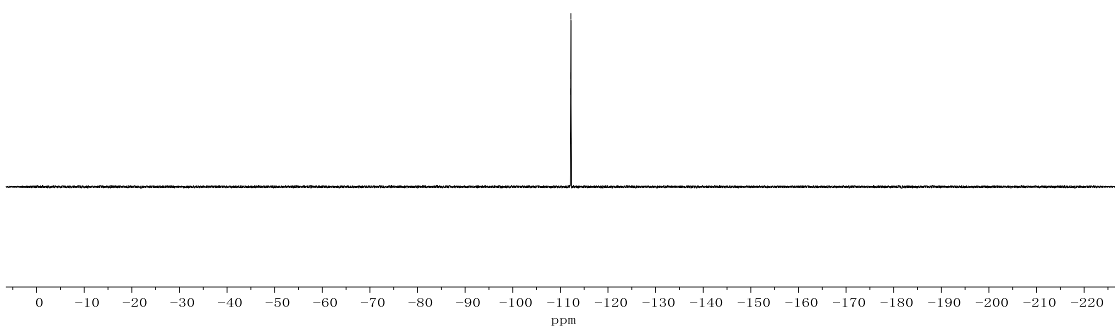

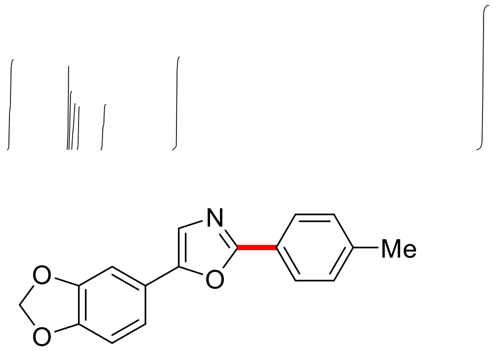

5ha
(400 MHz, $CDCl_3$)

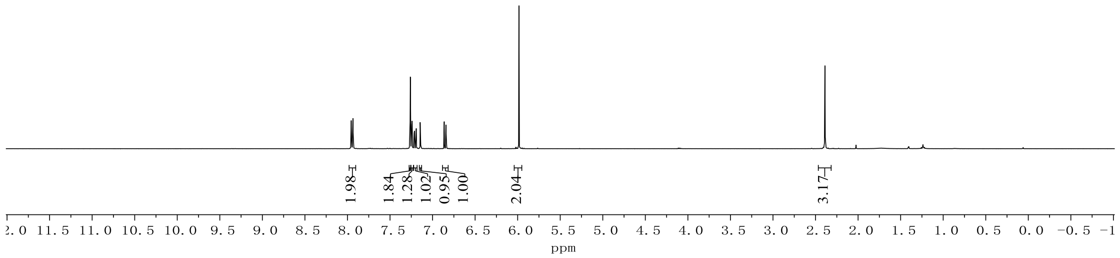

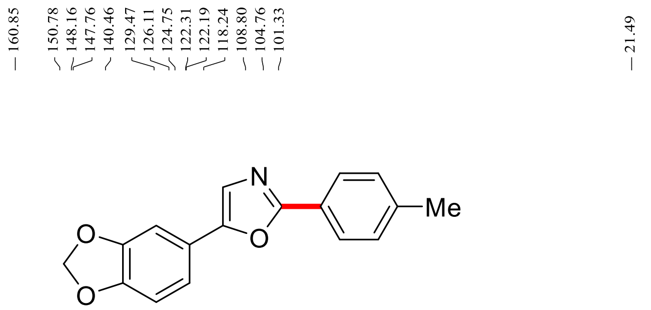

5ha
(100 MHz, $CDCl_3$)

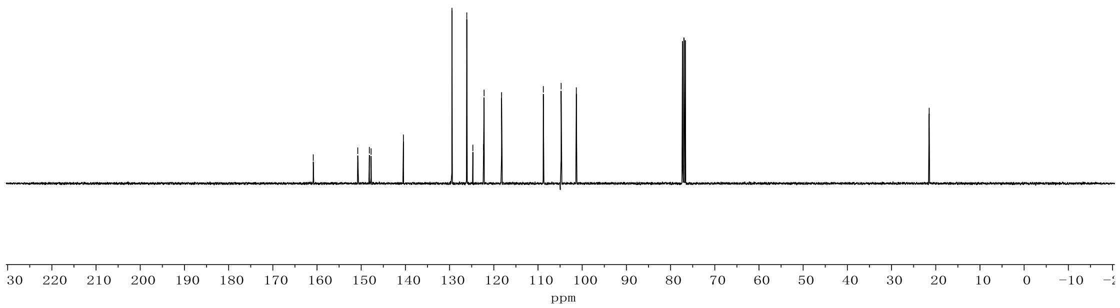

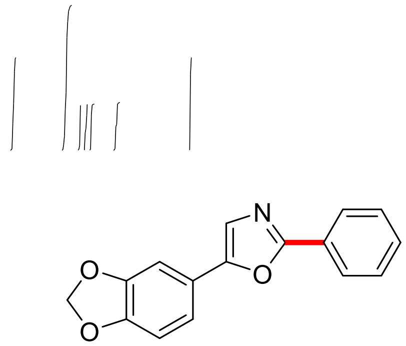

5hb
(400 MHz, $CDCl_3$)

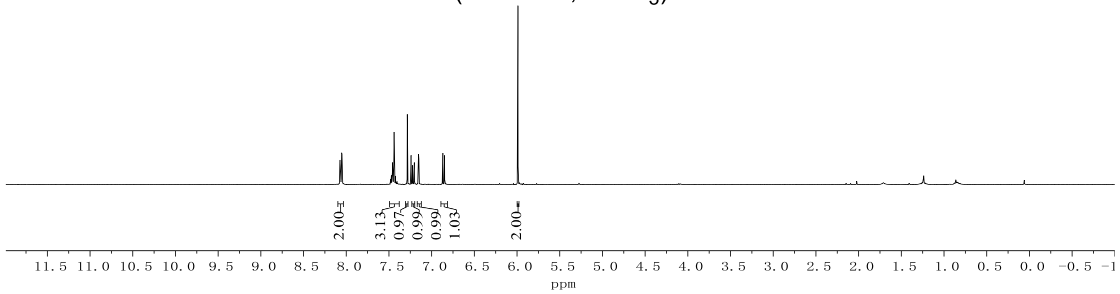

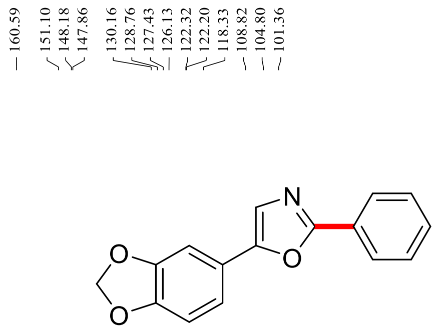

5hb
(100 MHz, $CDCl_3$)

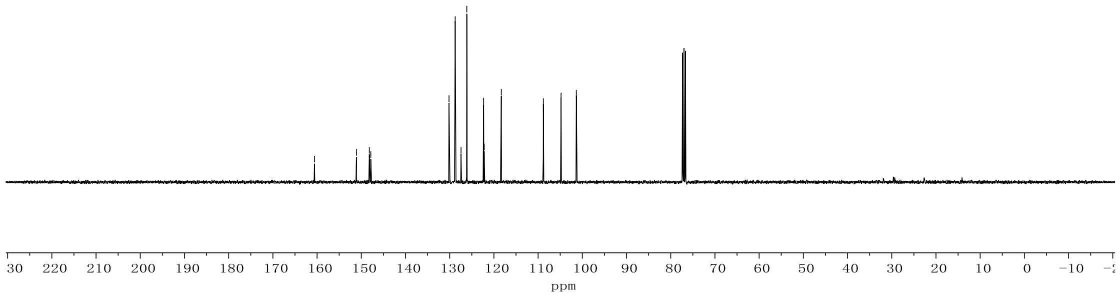

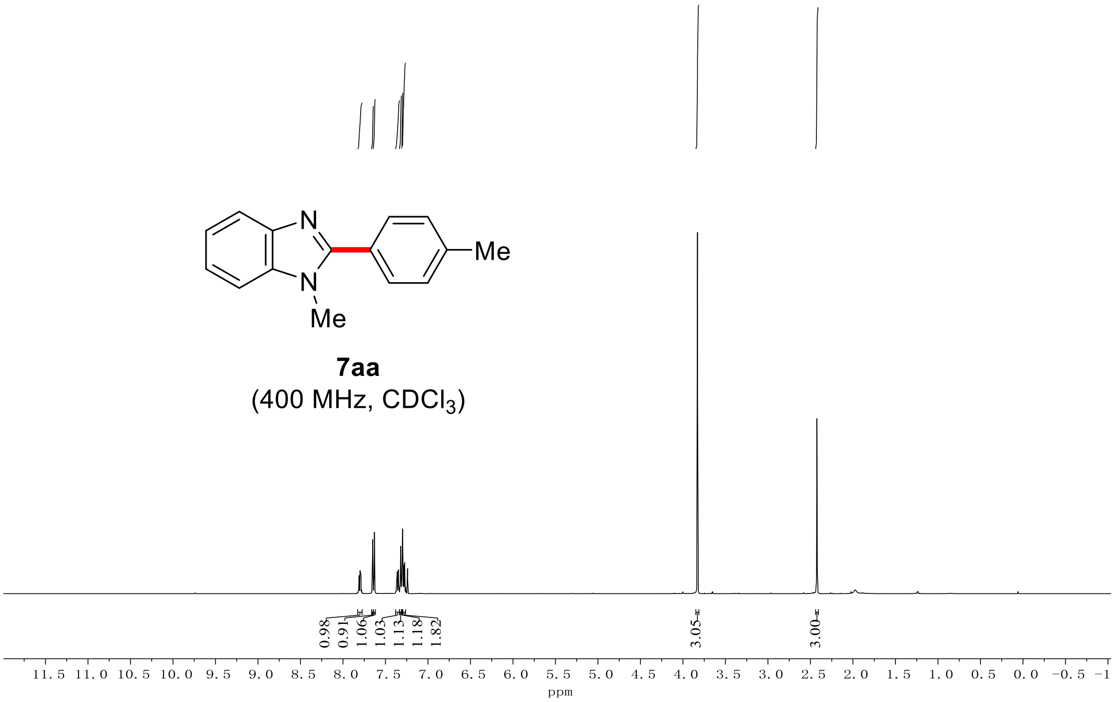
N
N
Me
Me
7aa
(400 MHz, CDCl3)
0.98
0.91
1.06
1.03
1.13
1.18
1.82
3.05
3.00
11.5 11.0 10.5 10.0 9.5 9.0 8.5 8.0 7.5 7.0 6.5 6.0 5.5 5.0 4.5 4.0 3.5 3.0 2.5 2.0 1.5 1.0 0.5 0.0 -0.5 -1
ppm

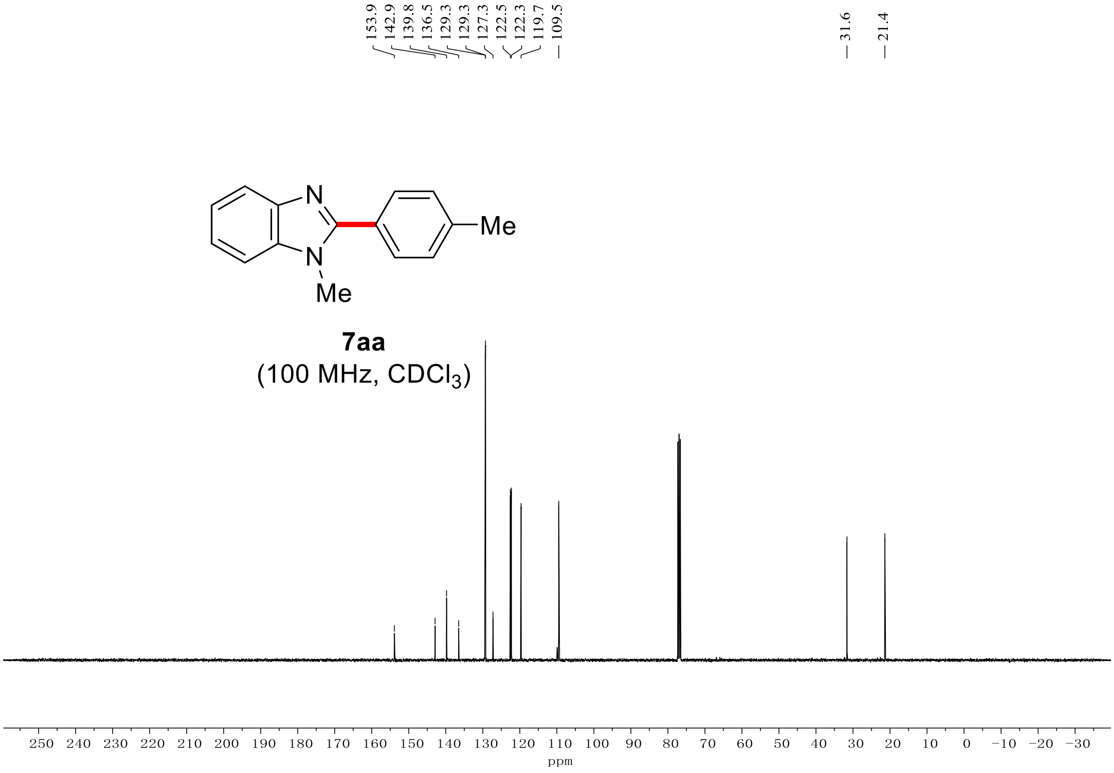
153.9
142.9
139.8
136.5
129.3
129.3
127.3
122.5
122.3
119.7
109.5
31.6
21.4
N
N
Me
Me
7aa
(100 MHz, CDCl3)
250 240 230 220 210 200 190 180 170 160 150 140 130 120 110 100 90 80 70 60 50 40 30 20 10 0 -10 -20 -30
ppm

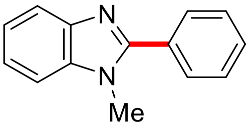

7ab
(400 MHz, $CDCl_3$)

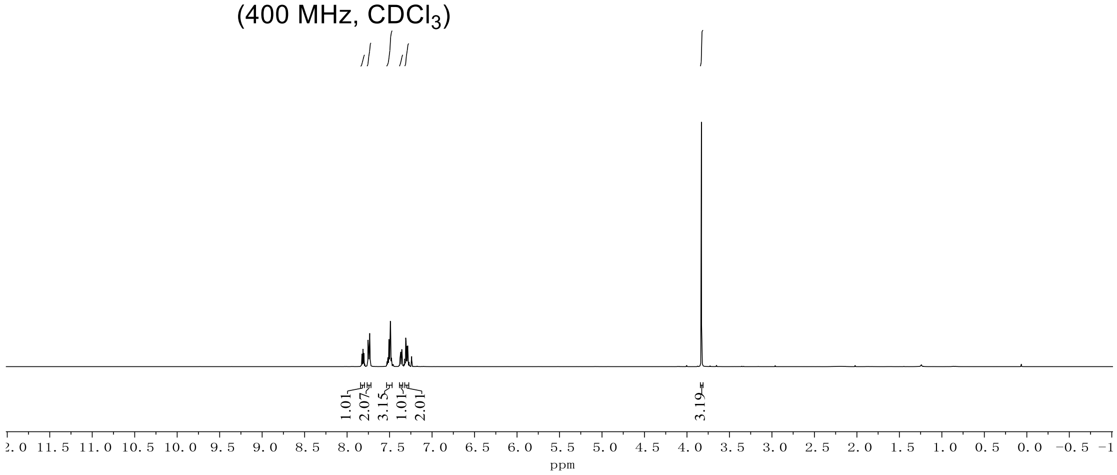

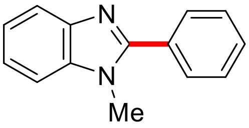

7ab
(100 MHz, $CDCl_3$)

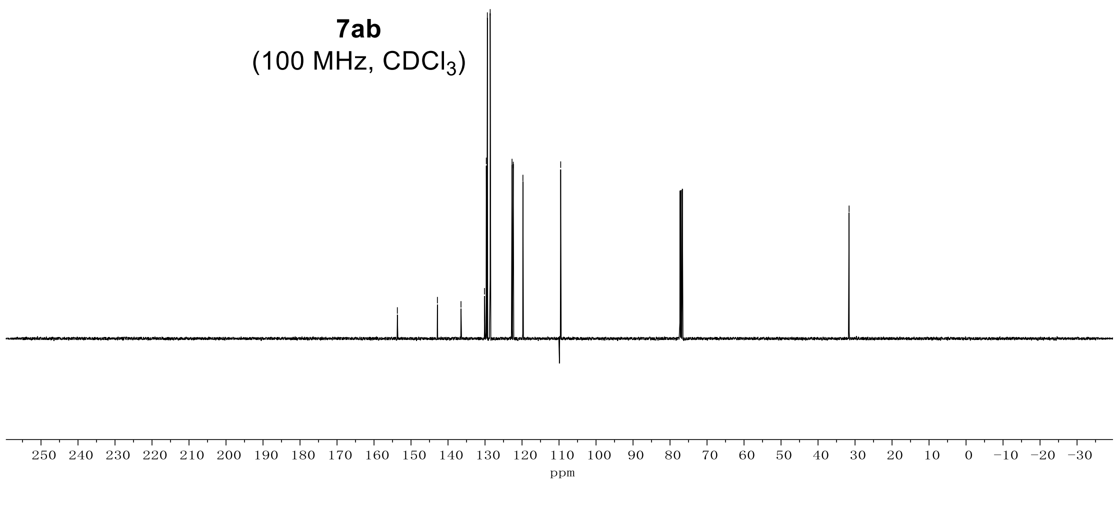

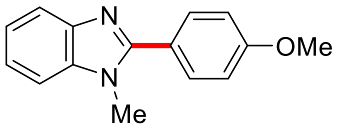

7ac
(400 MHz, $CDCl_3$)

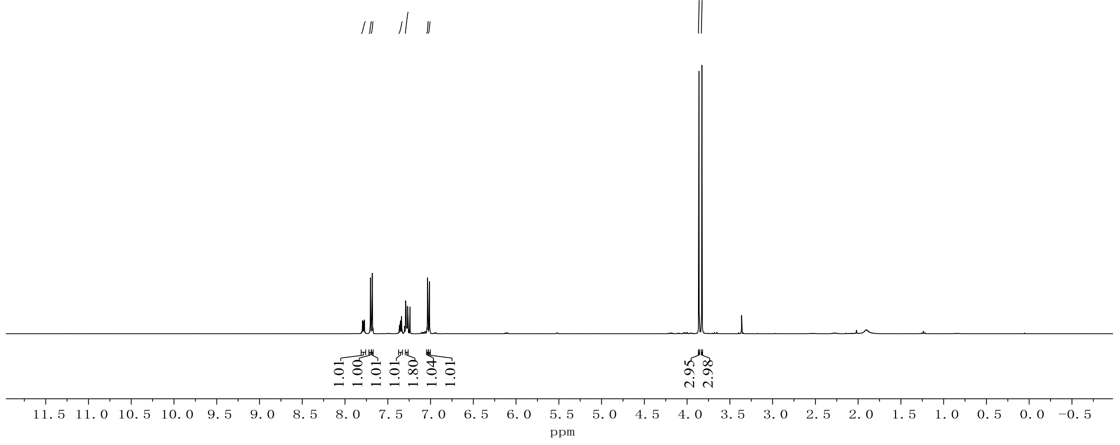

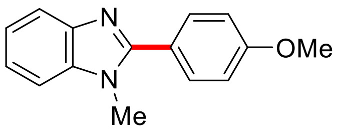

7ac
(100 MHz, $CDCl_3$)

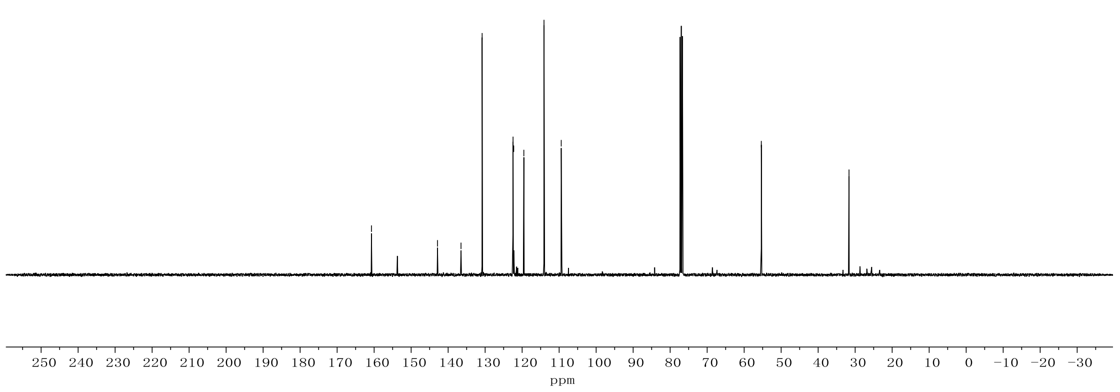

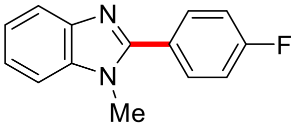

7ae
(400 MHz, $CDCl_3$)

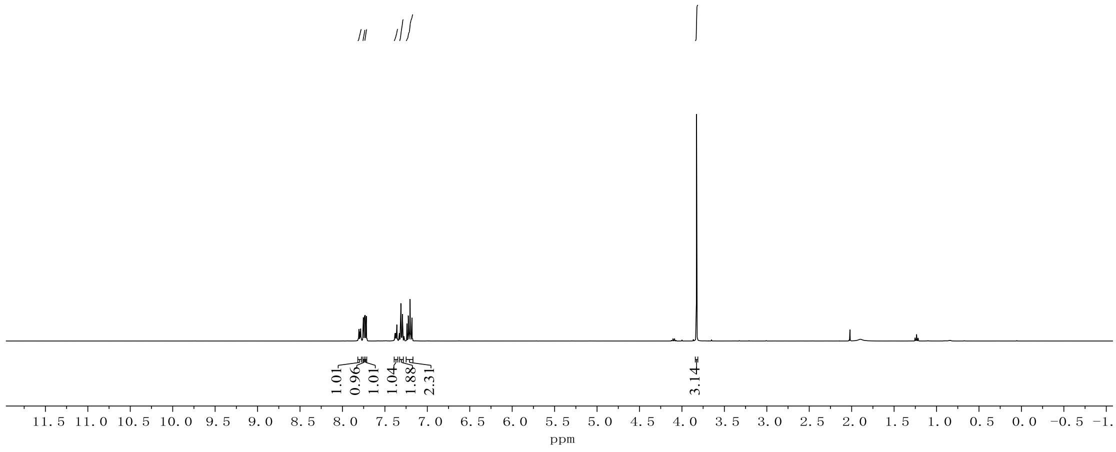

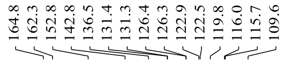

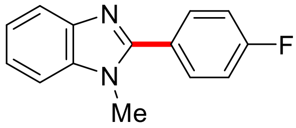

7ae
(100 MHz, $CDCl_3$)

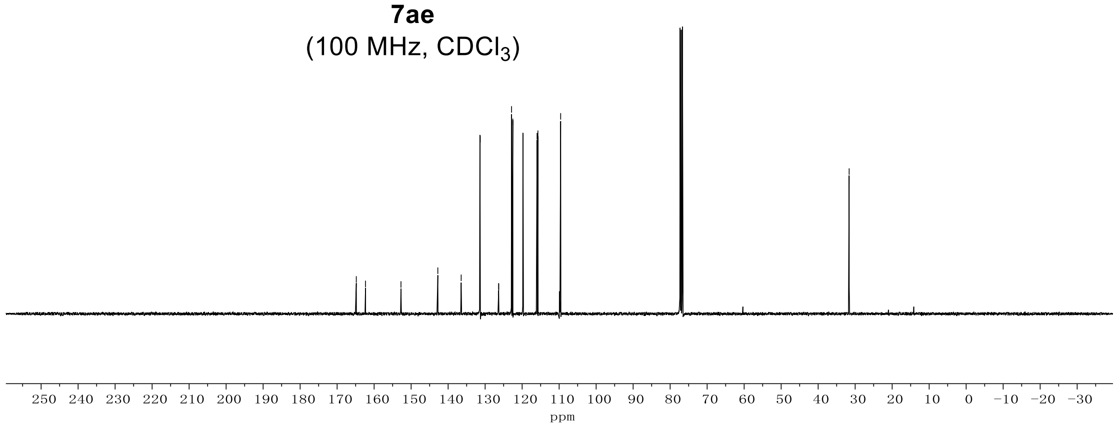

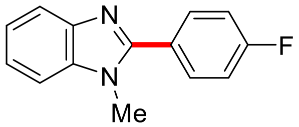

7ae
(282 MHz, $CDCl_3$)

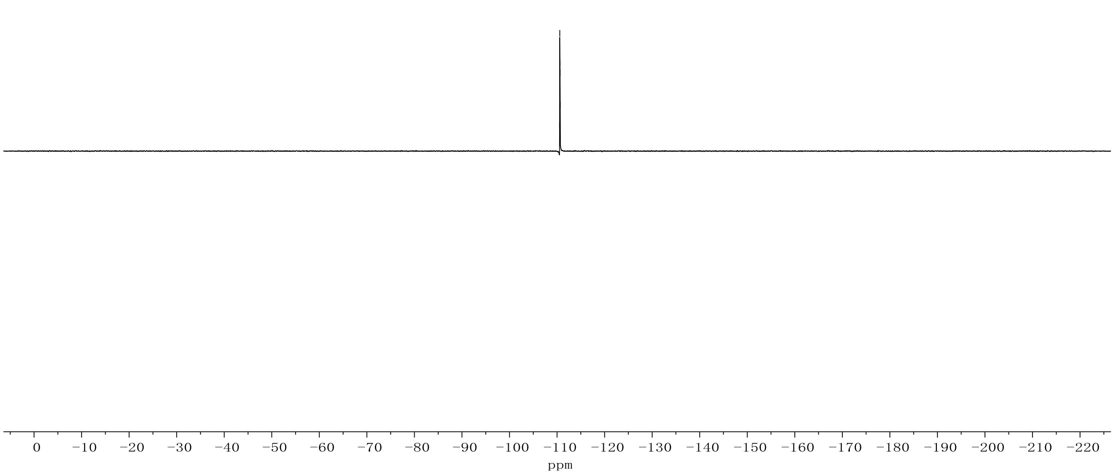

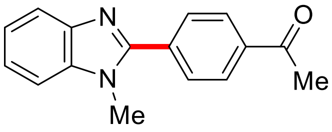

7ar
(400 MHz, $CDCl_3$)

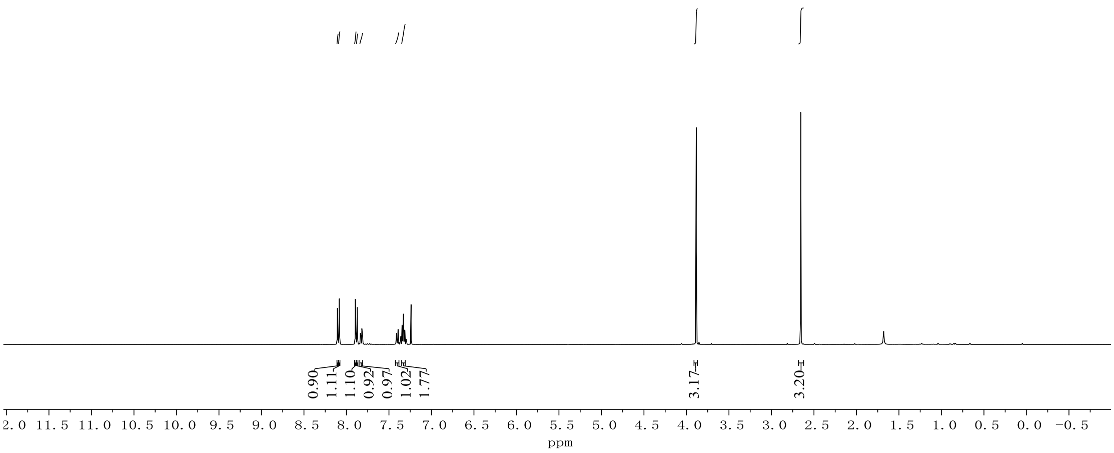

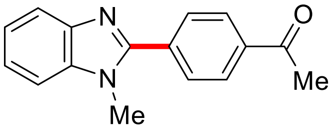

7ar
(100 MHz, $CDCl_3$)

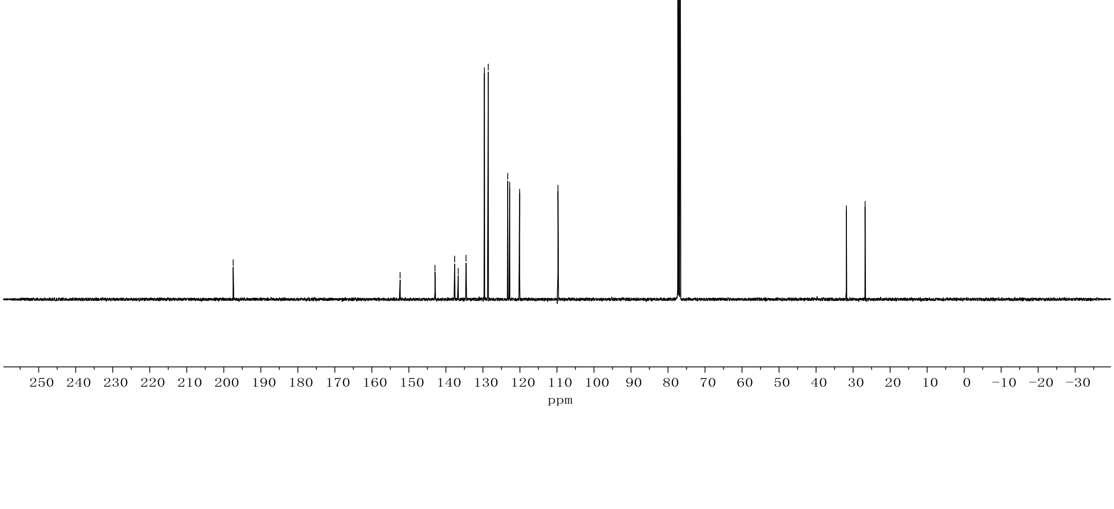

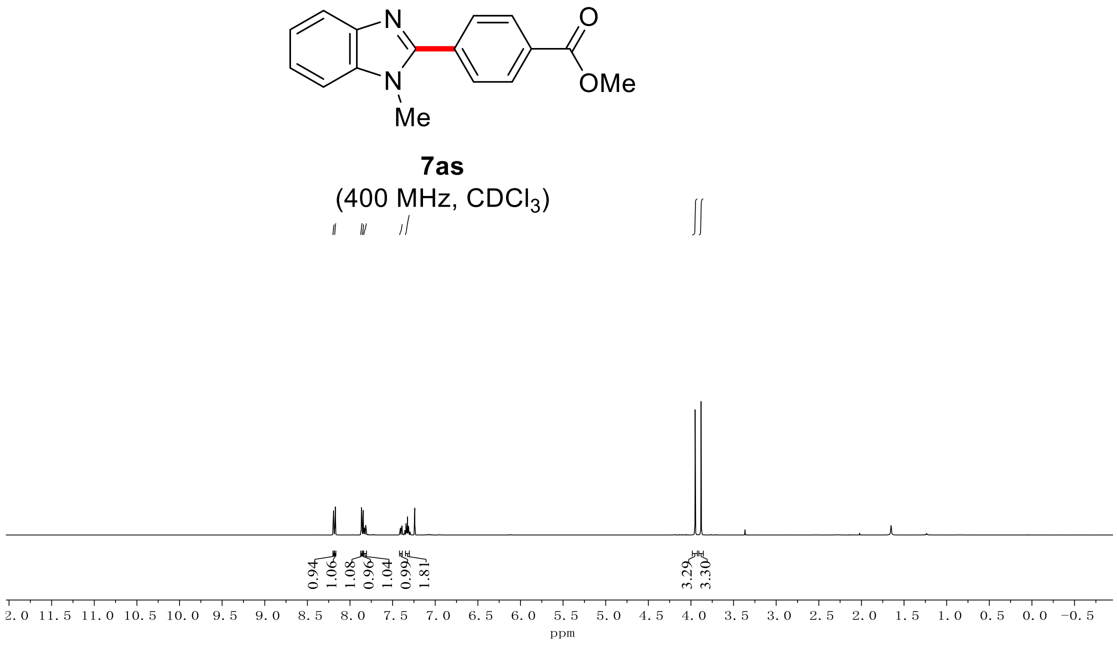

N
OMe
O
Me
7as
(400 MHz, CDCl3)
0.94
1.06
1.08
0.96
1.04
0.99
1.81
3.29
3.30
ppm

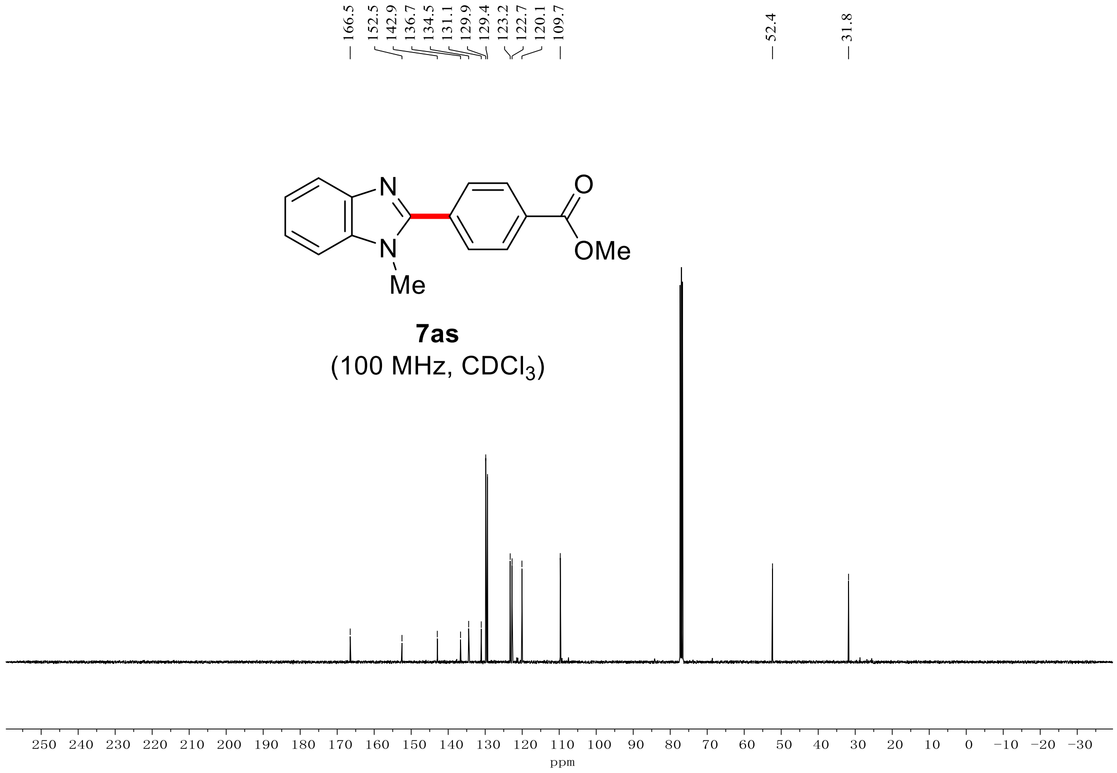

166.5
152.5
142.9
136.7
134.5
131.1
129.9
129.4
123.2
122.7
120.1
109.7
52.4
31.8
N
OMe
O
Me
7as
(100 MHz, CDCl3)
ppm

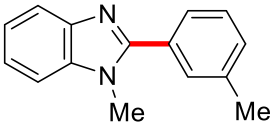

7ah
(400 MHz, $CDCl_3$)

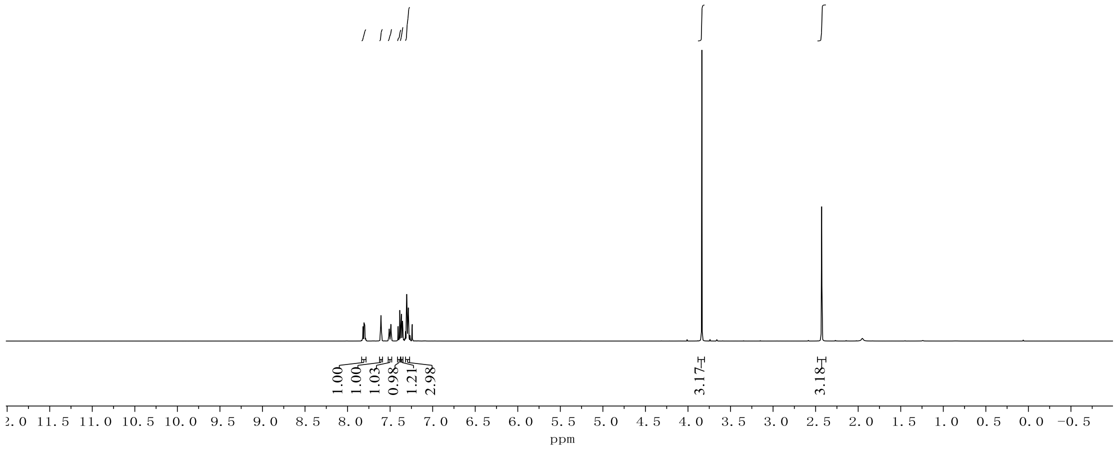

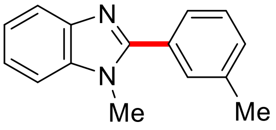

7ah
(100 MHz, $CDCl_3$)

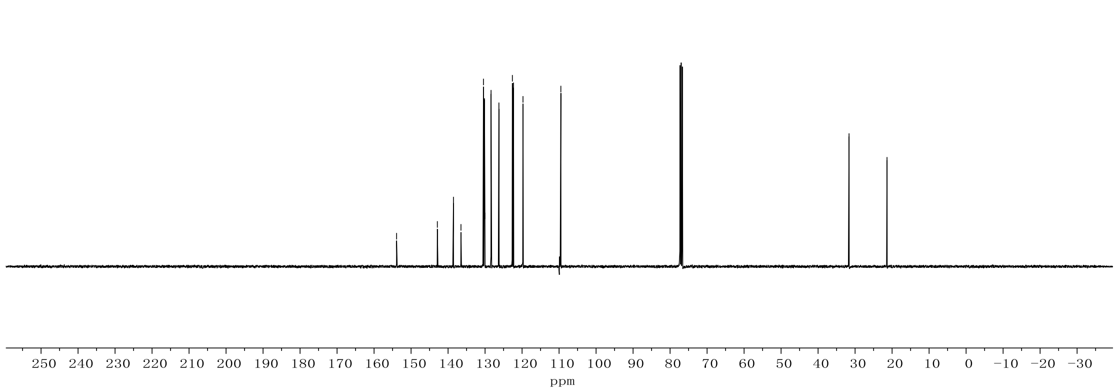

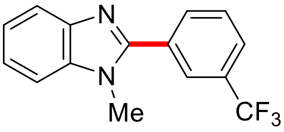

7aj
(400 MHz, $CDCl_3$)

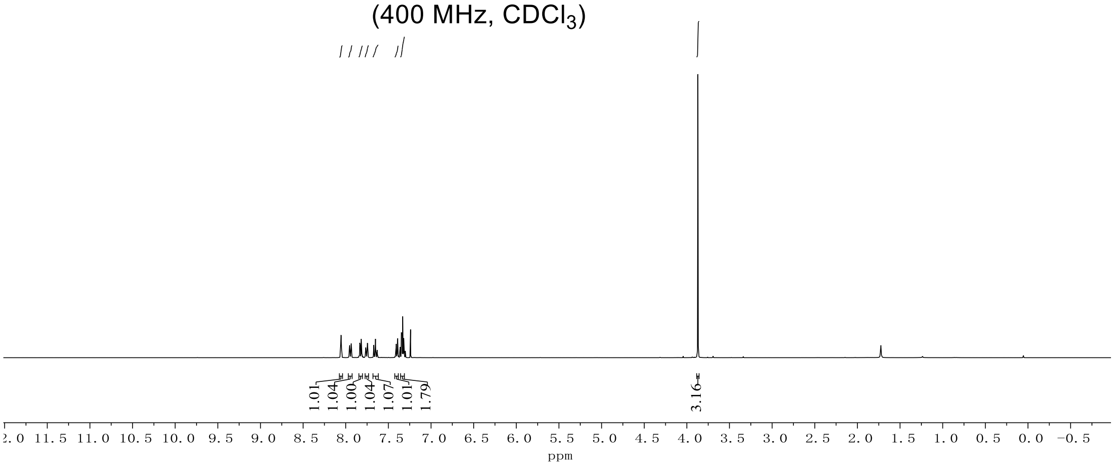

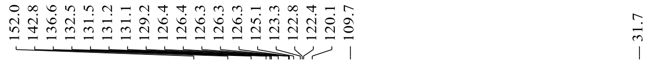

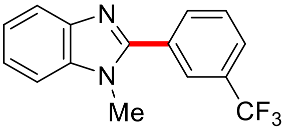

7aj
(100 MHz, $CDCl_3$)

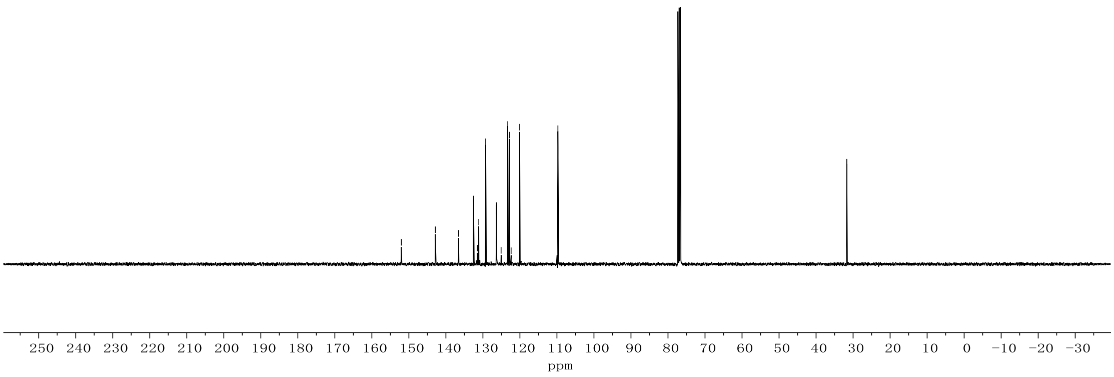

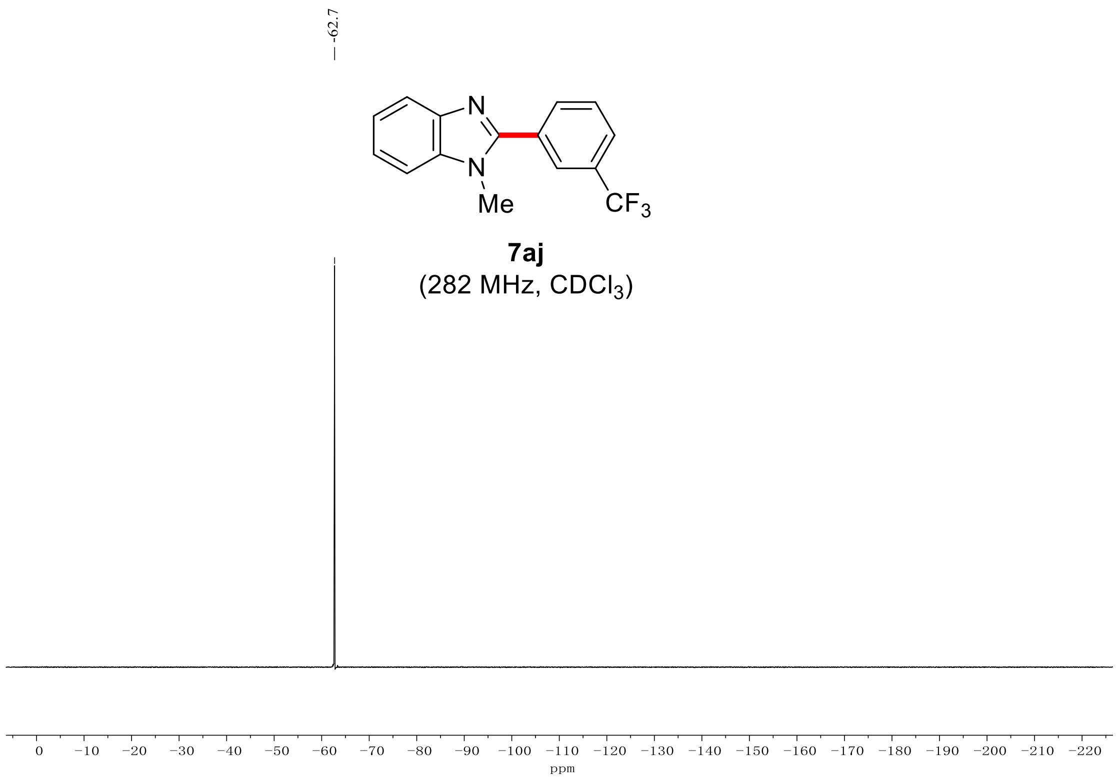
-62.7
N
N
Me
CF3
7aj
(282 MHz, CDCl3)
0 −10 −20 −30 −40 −50 −60 −70 −80 −90 −100 −110 −120 −130 −140 −150 −160 −170 −180 −190 −200 −210 −220
ppm

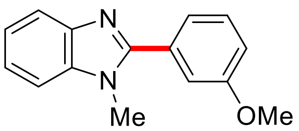

7ao

(400 MHz, $CDCl_3$)

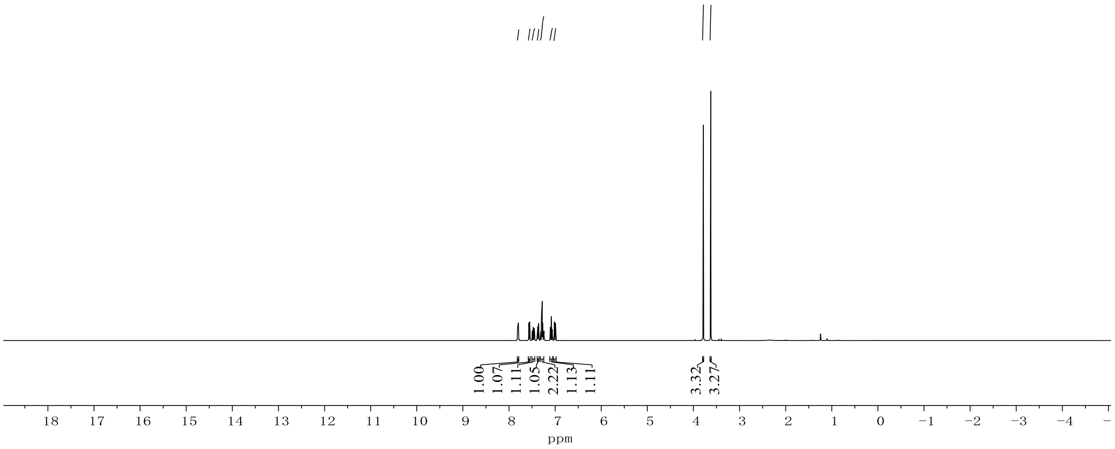

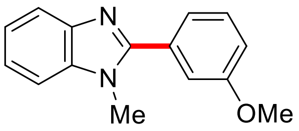

7ao

(100 MHz, $CDCl_3$)

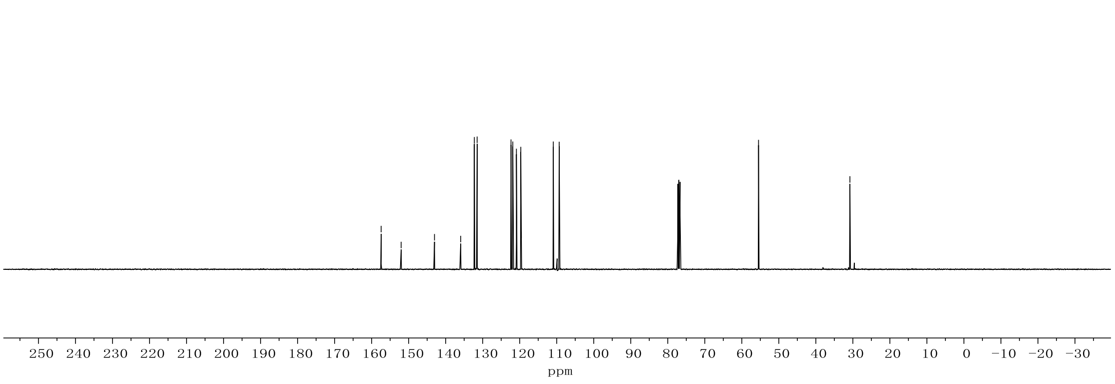

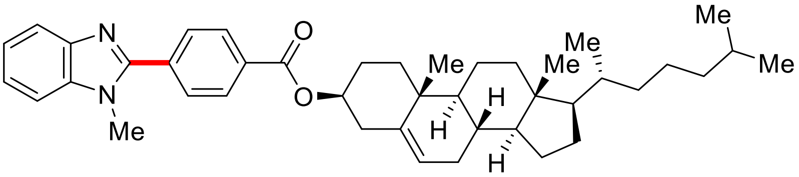

7at
(400 MHz, $CDCl_3$)

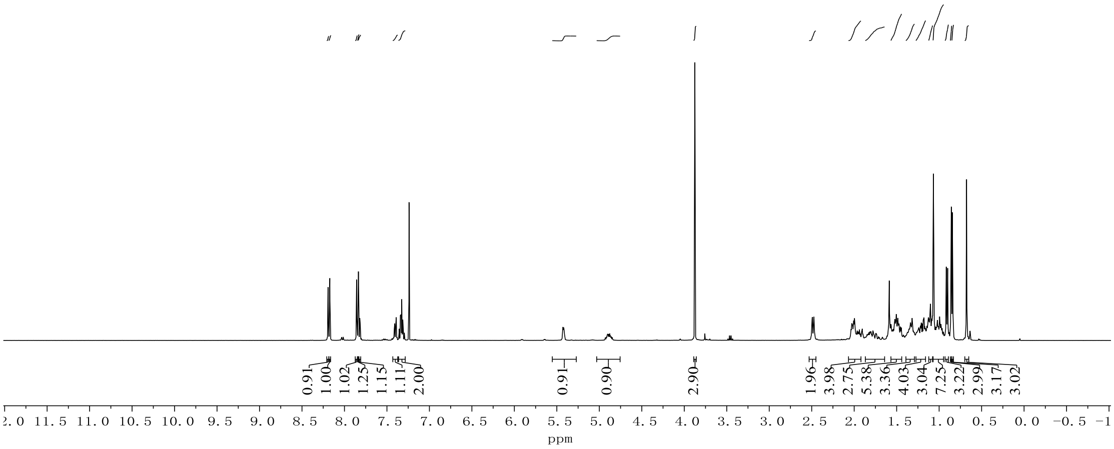

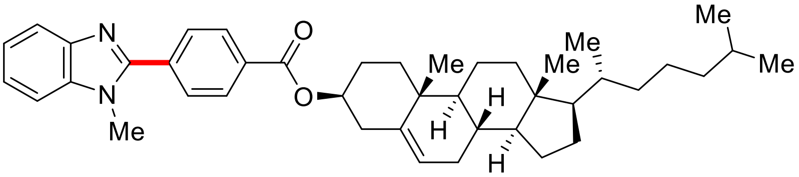

7at
(100 MHz, $CDCl_3$)

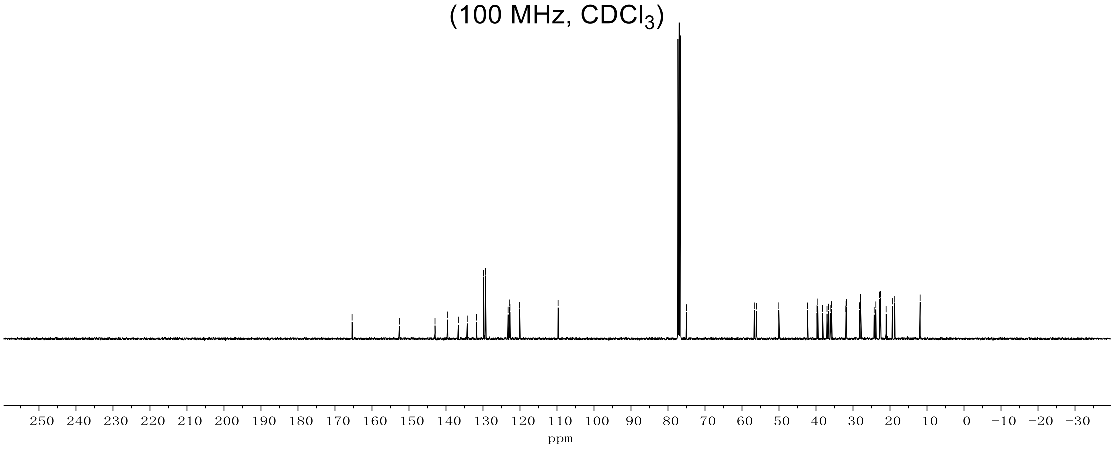

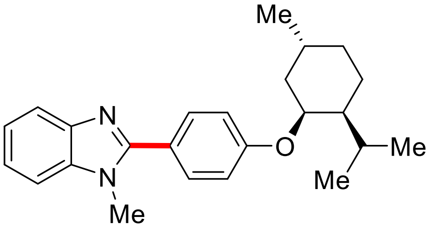

7au
(400 MHz, $CDCl_3$)

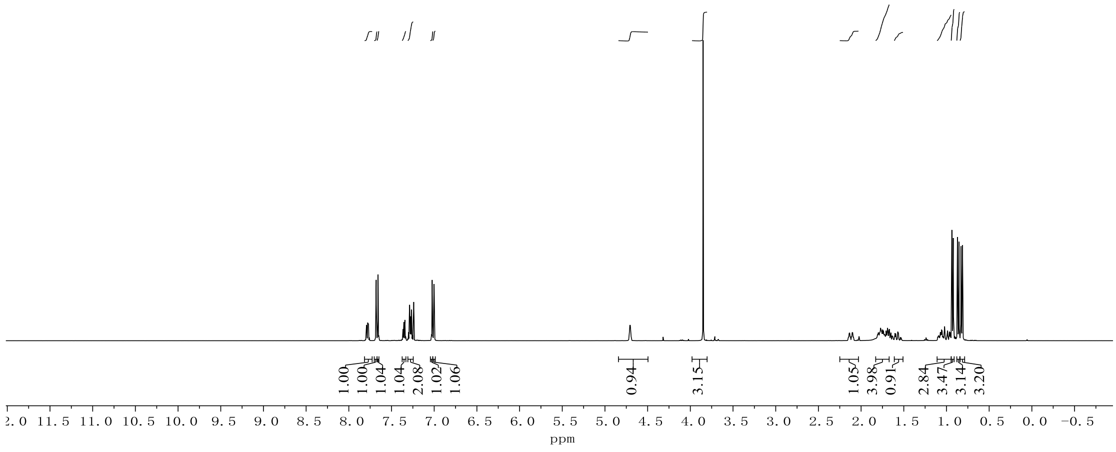

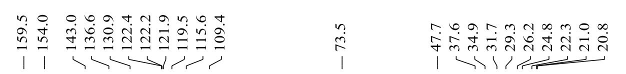

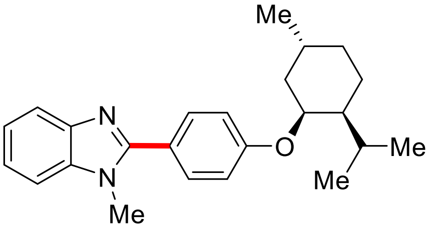

7ao
(100 MHz, $CDCl_3$)

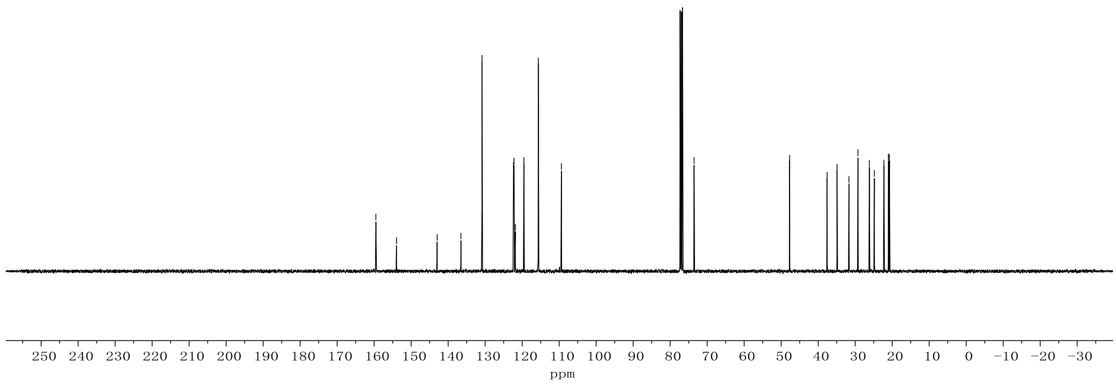

Supplement: Supplementary file 1 — Supplementary [file CHEM-26-3509-s001.pdf]
